# Targeting IL27RA Enhances Immunotherapy in Triple‐Negative Breast Cancer by Modulating Tumor Cells and the Tumor Microenvironment

**DOI:** 10.1002/advs.202516703

**Published:** 2026-01-04

**Authors:** Jiachi Xu, Qian Long, Meirong Zhou, Qitong Chen, Jing Peng, Qingchun Liang, Danhua Zhang, Hui Zhou, Wenjun Yi

**Affiliations:** ^1^ Department of General Surgery the Second Xiangya Hospital of Central South University Changsha China; ^2^ Department of Pathology the Second Xiangya Hospital of Central South University Changsha China

**Keywords:** IL27RA, breast cancer, immune microenvironment, immunotherapy, single‐cell sequencing

## Abstract

Immune checkpoint blockade (ICB) has improved outcomes for patients with triple‐negative breast cancer (TNBC), yet resistance remains widespread and its molecular basis is not fully understood. Through single‐cell RNA sequencing (scRNA‐seq) of paired pre‐ and post‐treatment tumor samples from patients who failed to achieve pathological complete response (non‐pCR) after neoadjuvant PD‐1 therapy, we identified a marked upregulation of interleukin‐27 receptor subunit alpha (IL27RA) in malignant epithelial cells within residual lesions. Integration with scRNA‐seq profiles from an independent cohort of three pCR patients showed that this *IL27RA* upregulation in malignant epithelium is largely restricted to non‐pCR residual tumors, and high IL27RA expression correlated with poor survival in TNBC cohorts. Mechanistically, IL27RA suppresses MHC‐I expression by activating the PI3K/AKT pathway—rather than the classical IL‐27/STAT axis—thereby impairing CD8⁺ T‐cell cytotoxic function. Inhibition of AKT reversed this phenotype and restored antigen‐specific killing. In orthotopic tumor models, mimicking systemic loss of *Il27ra* significantly reduced tumor growth and prolonged survival in immunocompetent mice, with single‐cell profiling indicating enhanced intratumoral T‐cell and NK‐cell effector activity. Collectively, our findings identify an epithelial‐intrinsic IL27RA–PI3K/AKT–MHC‐I axis as a central driver of immune evasion and ICB resistance in TNBC and support IL27RA as a promising therapeutic target for overcoming immunotherapy resistance.

## Graphical abstract

IL27RA upregulation drives immune evasion in TNBC by suppressing MHC‐I expression and reprogramming T/NK‐cell states, establishing an immune‐excluded tumor phenotype. Targeting this epithelial‐intrinsic IL27RA–PI3K/AKT axis offers a promising strategy to overcome immunotherapy resistance.

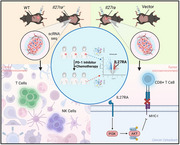

**Abbreviations**
APP: antigen‐presenting. pathway
BC: breast cancer
CCIs: cell‐cell interactions
CIs: confidence intervals
DAB: diaminobenzidine
DCs: dendritic cells
DEG: differential expression gene
ECL: enhanced chemiluminescence
EMR: electronic medical record
FFPE: formalin‐fixed paraffin‐embedded
GEO: Gene Expression Omnibus
GO: Gene Ontology
GZMB: granzyme B
HRP: horseradish peroxidase
HRs: hazard ratios
H&E: hematoxylin‐eosin
ICB: immune checkpoint blockade
ICP: inhibiting immune checkpoints
ICIs: immune checkpoint inhibitors
IDC: invasive ductal carcinoma
IFN‐γ: interferon‐γ
IHC: immunohistochemistry
ILCs: innate lymphoid cells
IL27RA: interleukin 27 receptor alpha
KEGG: Kyoto Encyclopedia of Genes and Genomes
KM: Kaplan‐Meier
KO: knockout
LDH: lactate dehydrogenase
MACS: magnetic‐activated cell sorting
MHC‐I: Major histocompatibility complex class I
NK: natural killer
NKT: natural killer T
OS: overall survival
PCA: principal component analysis
pCR: pathological complete response
PD‐L1: programmed cell death ligand‐1
PD‐1: programmed cell death protein 1
QC: quality control
RFS: recurrence‐free survival
RT‐qPCR: real‐time quantitative reverse transcription‐polymerase chain reaction
scRNA‐seq: single‐cell RNA sequencing
SD: standard deviation
SPF: specific pathogen‐free
ST: spatial transcriptomics
TCGA: The Cancer Genome Atlas
TF: transcription factor
TILs: tumor‐infiltrating lymphocytes
TMB: tumor mutation burden
TME: tumor microenvironment
TNBC: triple‐negative breast cancer
UMAP: uniform manifold approximation and projection
UMI: unique molecular identifier
WB: western blotting
WT: wild type

## 1 Background

Breast cancer (BC) is one of the most common malignant tumors in women [1], and there is an ongoing need to improve its treatment methods. Immunotherapy works by harnessing the body's own immune system, including the activation of both innate and adaptive immunity, to monitor and eliminate tumors [2]. The main approach of immunotherapy involves inhibiting immune checkpoints (ICP) on immune cells. Immune checkpoint inhibitors (ICIs) work by blocking the activity of ICP, thereby reactivating immune cells' response to tumor cells and improving the therapeutic effect on tumors [3, 4, 5, 6, 7]. Therefore, the abundance of immune components in the tumor microenvironment (TME) may directly influence the effectiveness of tumor immunotherapy [8, 9].

For a long time, BC has been considered a “cold” tumor due to its low lymphocyte infiltration and low tumor mutation burden (TMB) in the TME, making it unsuitable for immunotherapy [10, 11, 12]. However, triple‐negative breast cancer (TNBC) displays higher lymphocyte infiltration and relatively higher TMB, which establishes a favorable immune microenvironment for the application of immunotherapy and provides an antigenic foundation for immune cell recognition [13]. In fact, the emergence of immunotherapy has significantly improved the prognosis of TNBC [13, 14]. Unfortunately, not all TNBC patients benefit from this treatment. Some clinical studies have shown that although neoadjuvant immunotherapy combined with chemotherapy improves the pathological complete response rate by 10%–20% compared to chemotherapy alone, only 64.8% and 56.8% of patients, respectively, benefit from this treatment [14, 15]. This may be attributed to the heterogeneity of tumors among patients.

Currently, the understanding of tumors goes beyond the tumor cells themselves. The heterogeneity of the TME is increasingly recognized as a key factor influencing the development and treatment response of TNBC [16]. Components of the TME, including cancer epithelial cells, various infiltrating immune cells, and other essential stromal cells such as fibroblasts and vascular‐associated cells, continuously interact and communicate, leading to significant differences in the intrinsic tumor environments of cancer patients [17, 18]. Similarly, the majority of patients’ immune cells are continuously exposed to immune factors and various antigens within the microenvironment, which impairs their surveillance function, allowing tumor cells to escape immune detection [19, 20].

The advent of single‐cell RNA sequencing (scRNA‐seq) technology has filled the gap left by previous methods [21, 22]. In fact, many genes serve as markers for specific cell types, and simple global analyses are often insufficient to identify these differences. Furthermore, the impact of ICIs on the immune microenvironment is not limited to the target lymphocytes themselves. For example, programmed cell death protein 1 (PD‐1) is also expressed on the surface of cells other than T cells, such as B cells, natural killer (NK) cells, macrophages, dendritic cells (DCs), innate lymphoid cells (ILCs), neutrophils, and monocytes [23]. However, research on the effects of PD‐1 blockade therapy on these cells is still limited. To systematically reveal the changes in different cell populations under specific immunotherapies, single‐cell sequencing remains essential.

In this study, we conducted single‐cell transcriptomic and spatial transcriptomic (ST) analyses before and after treatment in TNBC patients who received neoadjuvant immunotherapy combined with chemotherapy but did not achieve pathological complete response (pCR). We identified Interleukin 27 receptor alpha (IL27RA) as a potential marker for immunotherapy resistance in breast cancer epithelial cells. We confirmed that IL27RA induces a decrease in the expression of major histocompatibility complex class I (MHC‐I) by activating the PI3K‐AKT pathway, leading to the efflux and functional decline of tumor‐infiltrating CD8^+^ T cells. This ultimately contributes to the mechanism of resistance to PD‐1 blockade therapy. Additionally, our study demonstrated that the ablation of IL27RA could enhance the efficacy of PD‐1 blockade therapy. Furthermore, we simulated the changes in the TME following IL27RA ablation therapy and used single‐cell transcriptomic sequencing to reveal the immune cell landscape after IL27RA ablation. We confirmed the impact of this therapy on immune cells and its potential to enhance immunotherapy effectiveness.

## 2 Materials and Methods

### 2.1 Study Cohort

This study enrolled six female patients with TNBC aged 44–64 years, all of whom were diagnosed by core needle biopsy in the Department of Pathology, the Second Xiangya Hospital of Central South University. All cases were invasive ductal carcinoma (IDC) with no radiologically confirmed distant metastasis. Patients received neoadjuvant therapy consisting of anthracycline/cyclophosphamide followed by paclitaxel combined with a PD‐1 inhibitor. According to imaging evaluation (RECIST v1.1) and pathological assessment (Miller‐Payne grading), three patients did not achieve pathological complete response (non‐pCR), while the other three achieved pCR. This study was conducted in accordance with the Declaration of Helsinki and was approved by the Ethics Committee of the Second Xiangya Hospital of Central South University (Approval No. LYF20230175). Written informed consent was obtained from all participants prior to data collection.

### 2.2 Mice

Female NOD/SCID mice (5–6 week old; Vital River, Beijing, China), C57BL/6J mice (GemPharmatech, Jiangsu, China), nude mice (BALB/c background; GemPharmatech), OT‐I mice (Cyagen, Jiangsu, China) and *Il27ra* knockout mice (C57BL/6J background; generated via CRISPR/Cas9 by GemPharmatech) were housed in a specific pathogen‐free (SPF) facility under controlled conditions: 12‐h light/dark cycle, 50 ± 5% humidity, and 25–27°C. All animals received autoclaved standard chow and sterile water ad libitum.

Animal experiments strictly followed the ARRIVE guidelines (Animal Research: Reporting of In Vivo Experiments) [24] and were approved by the Institutional Animal Care and Use Committee of the Second Xiangya Hospital of Central South University (Approval No.: 20240293).

### 2.3 Cell Culture

Human breast cancer cell lines MDA‐MB‐231, MDA‐MB‐468, and HCC1937 (purchased from Procell, Wuhan, China, in March 2022), the mouse breast cancer cell line EO771 (purchased from Procell, Wuhan, China, in March 2022), and human embryonic kidney cells HEK293T (purchased from Procell, Wuhan, China, in March 2022) were cultured in Dulbecco's Modified Eagle Medium (DMEM; Gibco, USA) supplemented with 10% fetal bovine serum (FBS; Gibco, USA) and 1% penicillin/streptomycin (Beyotime, Shanghai, China). Human normal breast epithelial cells MCF10A (purchased from Procell, Wuhan, China, in March 2022) were maintained in McCoy's 5A medium (Procell, China) with 10% FBS and 1% penicillin/streptomycin. Mouse breast cancer cells PY8119, kindly provided by Prof. Ceshi Chen (Kunming Medical University, China), were cultured in Ham's F12 medium (Gibco, USA) supplemented with 1 µg/mL hydrocortisone (Sigma‐Aldrich, USA), 50 µg/mL gentamicin (Sigma‐Aldrich, USA), 10 ng/mL epidermal growth factor (Peipo Biotech, China), 5 µg/mL bovine insulin (Solarbio, China), 10% FBS, and 1% penicillin/streptomycin. All cells were incubated at 37°C in a humidified 5% CO2 atmosphere, and mycoplasma contamination was routinely tested every 2 weeks using a PCR‐based detection kit (Beyotime, China). All cell lines were authenticated by STR profiling and confirmed to be mycoplasma‐free.

### 2.4 Single‐Cell and Spatial Transcriptomic Analysis

#### 2.4.1 Sample Collection and Sequencing

Formalin Fixed Paraffin Embedded (FFPE) biopsy tissues were collected from six early‐stage TNBC patients who received neoadjuvant chemo‐immunotherapy. Biopsies were obtained both before treatment and from residual lesions after treatment, resulting in a total of nine samples. Single‐cell RNA sequencing libraries were prepared by a certified service provider (OE Biotech Co., Shanghai, China) using the VITAcruizer Single‐Cell Preparation Instrument DP400 Library (E20000131, M20 Genomics, Hangzhou, China) & VITApilote High‐Throughput FFPE Single‐Cell Transcriptome Kit (R20123124, M20 Genomics), and sequencing was performed on an Illumina NovaSeq 6000 platform with paired‐end 150 bp reads. Spatial transcriptomic libraries were generated from adjacent FFPE sections using the 10× Genomics Visium Spatial Gene Expression solution and sequenced on the same platform. All downstream bioinformatic analyses were independently performed by the authors.

#### 2.4.2 ScRNA‐seq Data Processing and Analysis

Raw scRNA‐seq data were processed in R (v4.4.2) using the Seurat package (v5.0.0). Quality control was conducted to filter out low‐quality cells based on the following criteria: cells expressing fewer than 300 or more than 7000 genes, mitochondrial gene percentage (mt_percent) exceeding 10%, erythrocyte gene percentage (HB_percent) exceeding 3%, and unique molecular identifier (UMI) counts below 1000 or above the 97th percentile of nCount_RNA. Normalization was performed using Seurat's default LogNormalize method. Specifically, raw UMI counts per cell were normalized by the total expression, scaled by a factor of 10 000, and log‐transformed (log1p). Highly variable features were identified using FindVariableFeatures, and data were scaled with ScaleData while regressing out mt_percent before principal component analysis (PCA).

Dimensionality reduction and batch correction were carried out using the Harmony algorithm. Clustering was performed at multiple resolutions with the FindClusters function, and results were visualized in two dimensions using Uniform Manifold Approximation and Projection (UMAP). Differentially expressed genes were identified with the FindMarkers function (log2FC > 0.25, adjusted p < 0.05, and expression in ≥10% of cells). Functional enrichment analyses were performed using Gene Ontology (GO) [25] and Kyoto Encyclopedia of Genes and Genomes (KEGG) [26]. Cell–cell communication networks were inferred with the CellChat package.

### 2.5 Spatial Transcriptomic Data Processing and Analysis

For ST analysis, FFPE tissue sections from two biopsy samples taken before and after treatment from one patient were processed. Library construction and sequencing were outsourced to the same service provider, utilizing the 10× Genomics Visium Spatial Gene Expression Solution, and were performed strictly following the manufacturer's protocol. Sequencing was conducted on the DNBSEQ‐T7 (BGI, China) with paired‐end 150 bp reads. Subsequent data preprocessing, deconvolution, and spatial analysis were performed by the authors' team.

Raw spatial transcriptomic data, including gene expression matrices (filtered_feature_bc_matrix) and spatial imaging data (spatial folder), were imported into R using the Seurat package. A Seurat object was created from the raw count matrix, and spatial coordinates were aligned with corresponding histological images. Data normalization was performed using Seurat's SCTransform method, regressing out the mitochondrial gene percentage (percent_mito). PCA was applied for dimensionality reduction, and the first 30 principal components were used for clustering. UMAP was used for visualization of clustering results. Spatially variable features were identified using the FindSpatiallyVariableFeatures function, selecting the top 50 most variable features. To predict cell types in the spatial transcriptomic data, we utilized a pre‐annotated scRNA‐seq reference dataset (scRNA_harmony) and performed cell type transfer using the FindTransferAnchors and TransferData functions. Predicted cell type labels were visualized on spatial feature plots. The processed SPATA2 object was saved as an .rds file to facilitate reproducibility and further analysis. All analyses were conducted in R (v4.4.2) using Seurat (v5) and SPATA2 (v2.1).

### 2.6 Real‐Time Quantitative Reverse Transcription‐Polymerase Chain Reaction (RT‐qPCR)

cDNA was synthesized from the extracted RNA using the NovoScript Plus kit (E047, Novoprotein, China). Gene expression was detected using the NovoStart SYBR qPCR SuperMix Plus (E096, Novoprotein). PCR conditions: initial denaturation at 95°C for 1 min, followed by 35–45 cycles of denaturation at 95°C for 20 s and annealing at 60°C for 1 min.

### 2.7 Western Blotting (WB)

Cellular proteins were extracted using RIPA lysis buffer (89901, Thermo Fisher Scientific, USA). Protein concentration was determined by the BCA assay (P0012, Beyotime, China). Protein samples were separated by SDS‐PAGE and transferred onto PVDF membranes. The membranes were incubated overnight at 4°C with primary antibodies, including ACTIN (MA5‐11869, Thermo Fisher Scientific; 1:400 dilution), IL27RA (PA5‐96963, Thermo Fisher Scientific, 1:500 dilution), GAPDH (10494‐1‐AP, ProteinTech; 1:20 000 dilution), MHC‐I (15240‐1‐AP, ProteinTech; 1:1000 dilution), AKT (4691, Cell Signaling Technology; 1:500 dilution), P‐AKT (Ser473) (4060, Cell Signaling Technology; 1:500 dilution), INPP4B (MA5‐52508, Thermo Fisher Scientific; 1:1000 dilution), P‐STAT3 (MA5‐15193, Thermo Fisher Scientific, 1:1000 dilution), STAT3 (MA1‐13042, Thermo Fisher Scientific, 1:5000 dilution), Histone H3 (PA5‐16183, Thermo Fisher Scientific, 1:1000 dilution), and PD‐L1 (14‐5982‐82, eBioscience, 1:500 dilution). After incubation, membranes were washed three times with TBST. Horseradish peroxidase (HRP)‐conjugated secondary antibodies (SA00001‐2, Proteintech, China) were applied for 1 h at room temperature. Protein bands were detected using an enhanced chemiluminescence (ECL) kit (K‐12045‐D20, Advansta, USA) and visualized on a ChemiDoc imaging system (Bio‐Rad, USA). Band intensity was quantified via densitometric analysis using ImageJ software (v1.53, NIH, USA).

### 2.8 Hematoxylin‐Eosin (H&E) Staining and mIHC

Paraffin‐embedded tissue sections were deparaffinized, rehydrated, and stained using an H&E Staining Kit (Solarbio). For mIHC, the processing was outsourced to Aifang Biotechnology Co., Ltd. (Changsha, China). The sections were incubated overnight at 4°C with anti‐IL27RA primary antibody (PA5‐19984, Thermo Fisher Scientific), anti‐CD3 primary antibody (AF20162, Aifang), anti‐CD8 primary antibody (AF20211, Aifang), anti‐αSMA primary antibody (AFMM0002, Aifang), anti‐CK14 primary antibody (AFRM0091, Aifang), anti‐MHC‐I primary antibody (AWA13676, Abiowell) and anti‐PanCK primary antibody (AF20164, Aifang). Subsequently, they were incubated for 60 min with HRP‐conjugated secondary antibody (Proteintech, China). Fluorescent dye was applied for chromogenic labeling, and nuclei were counterstained with 4',6‐diamidino‐2‐phenylindole (DAPI).

### 2.9 Plasmid Construction and Generation of Stable Cell Line

Plasmid construction for shRNA and overexpression vectors was performed by Abiowell Biotechnology Co., Ltd. (Changsha, China). Knockdown plasmids were cloned into the pLKO.1‐puro vector, while overexpression plasmids utilized the pCDH‐puro vector as the backbone (primer sequences are listed in Table S2). Lentiviral particles were produced using these plasmids to infect target cells.

To generate stable *IL27RA* knockdown and overexpression cell lines, the corresponding shRNA or full‐length IL27RA cDNA was cloned into lentiviral expression backbones. Cells were infected at 30%–40% confluence with lentiviral particles at a multiplicity of infection (MOI) of 5 in the presence of 8 µg/mL polybrene (Sigma‐Aldrich, USA). 48 h after infection, puromycin (2 µg/mL) was added and maintained for 7–10 days to obtain stably transduced cell populations. IL27RA protein levels were examined by Western blotting to confirm efficient knockdown or overexpression. Control cell lines were generated in parallel by infection with lentivirus carrying a non‐targeting shRNA or an empty vector.

### 2.10 Colony Formation and Transwell Migration Assays

For colony formation assays, cells were seeded in 6–well plates (500 cells/well) and cultured for 14 days. Colonies were fixed with methanol, stained with 0.1% crystal violet, and counted (colonies >50 cells). Colony formation rate was calculated as:

$$\begin{array}{ccc} \mathrm{Colony}\;\mathrm{formation}\;\mathrm{rate}\left(\%\right) & = & \left(\mathrm{Number}\;\mathrm{of}\;\mathrm{colonies}\right.\\ & & \left./\mathrm{Number}\;\mathrm{of}\;\mathrm{seeded}\;\mathrm{cells}\right)\times 100 \end{array}$$

For Transwell migration assays, 1 × 10^5^ cells in serum‐free medium were seeded into the upper chamber (Corning, USA), while the lower chamber contained complete medium with 10% FBS. After 24 h, migrated cells on the membrane were fixed, stained, and quantified by counting five random fields using ImageJ software.

### 2.11 Orthotopic Tumor Model

Cancer cells were orthotopically injected into the fourth mammary fat pad of 6–week–old female mice under isoflurane (RWD Life Science, China) anesthesia. Tumor volume was monitored regularly using calipers and calculated as:

$$\mathrm{Tumor}\;\mathrm{volume}\;\left(mm^{3}\right)=0.52\times \left(\mathrm{Length}\;\times \;\mathrm{Widt}h^{2}\right)$$

### 2.12 Mouse Lung Tumorigenesis Experiment

The target cells were prepared into cell suspensions of the same concentration according to the requirements of the orthotopic tumorigenesis experiment and kept on ice. After local disinfection of the mouse tail, an appropriate amount of cancer cell suspension was slowly injected into the tail vein according to the experimental purpose. 2 weeks later, the tumorigenesis in the mouse lungs was checked, or the survival status of the mice was recorded according to the experimental needs.

### 2.13 Isolation of Tumor‐Infiltrating Lymphocytes (TILs)

A digestion buffer was prepared by supplementing *RPMI‐1640* basal medium (100 mL; Gibco) with 20 µL DNase I (Thermo Fisher Scientific) and 100 mg collagenase IV (Sangon, Shanghai, China), followed by pH adjustment to 7.2–7.4. Subcutaneous tumors were aseptically excised from tumor‐bearing mice, minced into 1–2 mm^3^ fragments, and transferred to 15 mL conical tubes. Tissue fragments were enzymatically digested in the prepared buffer (10 mL/g tissue) at 37°C with 200 rpm orbital shaking for 60 min. The digested suspension was filtered through a 70 µm cell strainer, washed with PBS, and centrifuged at 500 × g for 10 min. Erythrocytes were lysed using RBC lysis buffer (Bioshark, Beijing, China), followed by PBS washing and centrifugation. For lymphocyte enrichment, the cell pellet was resuspended in 3 mL 40% Percoll solution (GE, USA), gently overlaid with 70% Percoll, and centrifuged at 1260 × g for 30 min (acceleration/deceleration rates: 2/1). The intermediate leukocyte layer was collected, diluted threefold with PBS, and centrifuged at 500 × g for 10 min. The final pellet containing TILs was resuspended in *RPMI‐1640* complete medium for downstream assays.

### 2.14 Flow Cytometry

For surface marker staining, cells (1 × 10^6^/sample) were washed with PBS and incubated with fluorochrome‐conjugated antibodies (0.5 µL/sample; antibody panel in Table S3) in 100 µL PBS for 30 min at 4°C in the dark. Unbound antibodies were removed by centrifugation (1200 × g, 6 min), and cells were resuspended in 200 µL PBS for analysis. For intracellular marker staining, after surface staining, cells were fixed with 200 µL fixation buffer (15 min at 4°C; Thermo Fisher Scientific) and permeabilized with 2 mL 1× permeabilization buffer (Thermo Fisher Scientific). Antibodies (0.5 µL/sample) were added in 200 µL PBS, incubated for 30 min at 4°C, and washed twice with PBS. Viability was assessed using Zombie Aqua Fixable Viability Kit (Biolegend). Data were acquired on a CytoFLEX flow cytometer (Beckman Coulter, USA) and analyzed using FlowJo software (vX.0.7, BD Biosciences, USA).

### 2.15 In Vivo CD8^+^ T Cell Depletion

Mice were intraperitoneally injected with 100 µg anti‐CD8b monoclonal antibody (BE0223, Bio X Cell, USA) twice weekly, starting 3 days post tumor cell inoculation. Isotype control groups received equivalent doses of rat IgG1 (BE0083, Bio X Cell). Depletion efficiency was validated by flow cytometry.

### 2.16 Isolation of Immune Cells From Mouse Spleen

Mice were euthanized and surface‐sterilized by immersion in 75% ethanol for 5 min. Under aseptic conditions, the spleen was excised through a left subcostal incision, rinsed in ice‐cold PBS, and placed on a 70 µm cell strainer. Splenic tissue was mechanically dissociated using a syringe plunger while continuously flushing with PBS until complete homogenization. The cell suspension was centrifuged at 300 × g for 10 min, and erythrocytes were lysed with 2 mL RBC lysis buffer (10 min at RT). After PBS washing and centrifugation (300 × g, 5 min), the pellet was resuspended in PBS to obtain a single‐cell suspension of splenic immune cells.

### 2.17 Magnetic‐Activated Cell Sorting (MACS) of CD8^+^ T Cells

Cell suspensions were adjusted to 1 × 10^7^ cells/mL in MACS buffer (Miltenyi Biotec, Germany). CD8^+^ T cells were labeled with anti‐CD8 microbeads (20 µL per 1 × 10^7^ cells; Miltenyi Biotec) for 20 min at 4°C in the dark. Labeled cells were washed with 3 mL MACS buffer, centrifuged (300 × g, 10 min), and resuspended in 1 mL MACS buffer. LS columns (Miltenyi Biotec) were pre‐wetted with MACS buffer, loaded with the cell suspension, and washed three times (3 mL/wash). CD8^+^ T cells were eluted in 5 mL MACS buffer after column removal from the magnetic stand, centrifuged (300 × g, 5 min), and resuspended in *RPMI‐1640* complete medium for subsequent assays.

### 2.18 In Vitro Activation of CD8^+^ T Cells

Sterile 6‐well plates were coated with anti‐CD3 antibody (2 µg/mL in PBS, 2 mL/well; Thermo Fisher Scientific) for 2 h at 37°C under 5% CO_2_. Unbound antibodies were aspirated, and CD8^+^ T cells (3 × 10^6^ cells/mL in *RPMI‐1640* medium supplemented with 5 µg/mL anti‐CD28 antibody; Thermo Fisher Scientific) were added (2 mL/well). Cells were cultured for 24–72 h to achieve full activation, as confirmed via flow cytometry.

### 2.19 Co‐Culture of CD8^+^ T Cells With Breast Cancer Cells

Breast cancer cells were seeded in 24‐well plates at a density of 5 × 10^4^ cells/well in *RPMI‐1640* complete medium and allowed to adhere for 4–6 h in a 5% CO_2_ incubator at 37°C. Activated CD8^+^ T cells (as described in Section 2.18) were added and co‐cultured for 48 h under standard conditions, followed by cytotoxicity assessment.

### 2.20 Lactate Dehydrogenase (LDH) Cytotoxicity Assay

After 24 h of co‐culture, 100 µL of supernatant was collected from each well and centrifuged (300 × g, 5 min) to remove cellular debris. LDH release was quantified using a LDH Cytotoxicity Assay Kit (C0016, Beyotime) according to the manufacturer's protocol.

### 2.21 CFSE/7‐AAD Staining for Cell Viability and Cytotoxicity

CFSE stock solution (5 mM) was prepared by dissolving lyophilized CFSE (423801, BioLegend) in 36 µL DMSO. Working solution (5 µM) was diluted in PBS (1:1000) immediately before use. Target cells (5 × 10^6^ cells/mL) were labeled with CFSE by incubation in 5 µM working solution for 20 min at 37°C in the dark, followed by quenching with 5 volumes of ice‐cold complete medium containing 10% FBS. After washing, labeled cells were co‐cultured with CD8^+^ T cells as described in Section 2.19.

To assess cell death, 7‐AAD (C1053S, Beyotime) was added to the co‐culture (50 µL/mL) 15 min prior to harvest. Cells were analyzed on a CytoFLEX flow cytometer, with CFSE^+^/7‐AAD^−^ cells representing viable target cells and CFSE^+^/7‐AAD^+^ cells indicating dead/dying targets. Data were processed using FlowJo software.

### 2.22 Antigen‐Specific CD8^+^ T Cell Cytotoxicity Assay

Breast cancer cells were pulsed with 2 µg/mL SIINFEKL peptide (OVA257‐264, HY‐P1489, MedChemExpress, USA) in serum‐free *RPMI‐1640* for 2 h at 37°C. Cells were washed three times with PBS to remove unbound peptides and seeded in 24‐well plates. Splenic CD8^+^ T cells isolated from OT‐I transgenic mice were activated as described in Section 2.18 and co‐cultured with peptide‐pulsed target cells. Cytotoxicity was quantified via LDH release (Section 2.20) and CFSE/7‐AAD flow cytometry (Section 2.21) after 24 h of co‐culture.

### 2.23 Transcriptomic Profiling

RNA sequencing was performed by Personalbio Biotechnology Co., Ltd. (Shanghai, China). Total RNA was extracted from PY8119 cells (three biological replicates per group) using TRIzol reagent (Thermo Fisher Scientific), followed by quality assessment (A260/A280 ≥1.8, A260/A230 ≥2.0; Nanodrop 2000, Thermo Fisher Scientific). Ribosomal RNA was depleted using the Ribo‐Zero Gold rRNA Removal Kit (Illumina, USA). Strand‐specific RNA‐seq libraries were prepared with the NEBNext Ultra II RNA Library Prep Kit (NEB, USA), including cDNA synthesis (random hexamer priming), end repair, A‐tailing, adapter ligation, and PCR enrichment (15 cycles). Libraries were quantified (Agilent 2100 Bioanalyzer) and sequenced on an Illumina NovaSeq 6000 platform (PE150, ≥20 million reads/sample).

### 2.24 Immunofluorescence Staining

For cell samples, sterile glass coverslips (12 mm diameter) were placed in 24‐well plates, Cells (1–5 × 10^4^/mL) were seeded and cultured for 12–24 h. Tissue sections (5 µm) from paraffin‐embedded samples were deparaffinized and rehydrated.

The staining protocol was as follows: Fixation was performed using 4% neutral‐buffered formaldehyde at room temperature for 15 min. For permeabilization of nuclear and cytoplasmic targets, cells were treated with 0.5% Triton X‐100 in TBS for 10 min at room temperature. Blocking was done using 5% BSA in TBS for 30 min at 37°C. Primary antibodies were diluted in blocking buffer (1:100–1:500) and incubated overnight at 4°C in a humidified chamber. Alexa Fluor‐conjugated secondary antibodies (1:500; Invitrogen) were incubated for 1 h at 37°C. Nuclei were counterstained with DAPI (1 µg/mL) for 5 min at room temperature. Coverslips were mounted with ProLong Gold Antifade Mountant (Thermo Fisher Scientific) and imaged using a Zeiss LSM 900 confocal microscope (Carl Zeiss, Germany). Z‐stack images were processed using ZEN 3.0 software.

### 2.25 Pharmacological Inhibition of AKT

MK‐2206 (AKT1/2/3 inhibitor, S1078, Selleckchem, USA) was dissolved in DMSO to prepare a 10 mM stock solution (aliquoted and stored at −80°C). For in vitro studies, cells seeded in 6‐well plates (70%–80% confluency) were treated with MK‐2206 (1–10 µM in complete medium), with control groups receiving equivalent DMSO (final concentration ≤0.1%) or medium alone. Cells were incubated under standard culture conditions (37°C, 5% CO_2_) for 72 h prior to downstream analyses.

### 2.26 In Vivo Anti‐PD‐1 Immunotherapy

Orthotopic breast tumors were established in 6‐week‐old female C57BL/6J mice by injecting *Il27ra*‐wildtype or knockout tumor cells into the fourth mammary fat pad, as detailed in Section 2.11. When tumors reached 50–100 mm^3^ (Day 7 post‐inoculation), mice were randomized into treatment groups (n = 5/group) and intraperitoneally administered either anti‐PD‐1 antibody (BE0146, Bio X Cell) or isotype control IgG (BE0094, Bio X Cell) at 200 µg/dose every 3 days. Mice were euthanized on Day 19 for tumor excision, with tissues processed for flow cytometry (Section 2.14) or immunohistochemistry (Section 2.8).

### 2.27 Genotyping of Transgenic Mice

A 2‐mm tail tip was collected under aseptic conditions after disinfecting with 75% ethanol. Genomic DNA was extracted by incubating tail tissue in 100 µL alkaline lysis buffer (25 mM NaOH, 0.2 mM EDTA) at 98°C for 30 min, followed by neutralization with 100 µL 40 mM Tris‐HCl (pH 5.5). PCR amplification was performed using gene‐specific primers (Table S4). Amplicons were resolved on 2% agarose gels, with wildtype and mutant alleles distinguished by expected band sizes.

### 2.28 Isolation of Tumor‐Infiltrating NK Cells

Tumor‐infiltrating cell suspensions were obtained using the method mentioned in section 2.13. Tumor‐infiltrating NK cells were enriched using the EasySep Mouse CD49b (DX5) Positive Selection Kit (18755, STEMCELL Technologies, Canada). Resuspend cells at 1 × 10^7^ cells/mL in EasySep Buffer, add 10 µL/mL CD49b Biotin Selection Cocktail, and incubate at 4°C for 10 min. Then, 20 µL/mL of EasySep streptavidin magnetic particles were added, mix gently and incubate at 4°C for 5 min. Finally, Place the tube into an EasySep Magnet for 3 min, discard supernatant, and repeat washing steps twice. Resuspend the magnetically retained CD49b^+^ NK cells in complete RPMI‐1640 medium.

### 2.29 Single‐cell RNA Sequencing of CD45⁺ Immune Cells From Orthotopic Tumors and Data Processing

Orthotopic tumors were established as described in Section 2.11. Tumors were dissected, and CD45^+^ immune cells were isolated for single‐cell RNA sequencing (10× Genomics Chromium Platform). Differential gene expression between *Il27ra* knockout and wild‐type mice was analyzed using Seurat (v5.1.0) with the following criteria: genes expressed in ≥50% of cells per group, with |log_2_(fold change)| > 0.5 and an adjusted *p*‐value < 0.05 (using the Wilcoxon rank‐sum test and Benjamini‐Hochberg correction). Volcano plots were generated using the ggplot2 package (v3.5.1). Transcription factor (TF) activity in NK cell subsets was inferred via DoRothEA (v1.18.0) [27], retaining only high‐confidence TF‐target interactions (A/B/C confidence levels). Pseudotime trajectories of NK cells were reconstructed using Monocle3 (v1.3.7). Cytotoxicity (*Gzma*, *Gzmb*, *Gzmm*, *Gzmk*, *Prf1*, *Ctsw*), inflammatory (*Ccl2*, *Ccl3*, *Ccl4*, *Ccl5*, *Cxcl9*, *Cxcl10*, *Il1b*, *Il6*, *Il7*, *Il15*, *Il18*), and stress response (*Bag3*, *Calu*, *Dnajb1*, *Dusp1*, *Egr1*, *Fos*, *Fosb*, *Hif1a*, *Hsp90aa1*, *Hsp90ab1*, *Hsp90b1*, *Hspa1a*, *Hspa1b*, *Hspb1*, *Hsph1*, *Ier2*, *Jun*, *Junb*, *Nfkbia*, *Nfkbiz*, *Rgs2*, *Slc2a3*, *Socs3*, *Ubc*, *Zfand2a*, *Zfp36*, *Zfp36l1*) gene modules were scored using the AddModuleScore function in Seurat.

### 2.30 Bioinformatics and Visualization

Kaplan‐Meier (KM) survival analyses were performed using the R survival package (v3.7‐0). Public survival data from the Kaplan‐Meier Plotter database (https://kmplot.com/analysis/, accessed November 2025) were analyzed as described [28]. Protein‐protein interaction networks for IL27RA were generated via STRING (v12.0, https://string‐db.org/). Bar plots and survival curves from experimental data were created using GraphPad Prism (v9.5, USA). Bioinformatics plots were generated in R (v4.4.2) with ggplot2.

### 2.31 Statistical Analysis

Data pre‐processing included quality‐control filtering, normalization, and transformation steps. For scRNA‐seq data, low‐quality cells were removed based on gene count, mitochondrial gene percentage, and UMI thresholds. Raw UMI counts were normalized by library size and log‐transformed (log1p). Spatial transcriptomic data were normalized using the SCTransform method. Technical covariates were regressed out during data scaling, and batch correction across samples was performed using the Harmony algorithm. Outliers were assessed using predefined quality‐control metrics and were excluded only when attributable to technical errors.

Continuous data are presented as mean ± standard deviation (SD). Sample sizes (n) for each experiment are indicated in the corresponding figure legends and refer to biological replicates unless otherwise specified. For comparisons of categorical variables, Chi‐square tests were used when expected frequencies were ≥5, and Fisher's exact test was applied when expected frequencies were <5. Kaplan–Meier survival curves were compared using the log‐rank test. For continuous variables, normality and variance homogeneity were evaluated using the Shapiro–Wilk and Levene tests. When assumptions were met, two‐sided Student's *t*‐tests were used; otherwise, non‐parametric tests such as the Mann–Whitney U test or Kruskal–Wallis test were applied. A significance level of α = 0.05 was considered statistically significant. Statistical significance is denoted as follows: ns (*p* > 0.05), *p < 0.05, **p < 0.01, ***p < 0.001, and ****p < 0.0001. All statistical analyses were performed using GraphPad Prism (version 9.5) or R software (version 4.4.2).

## 3 Results

### 3.1 ScRNA‐seq Delineates the TNBC Landscape Before and After PD‐1 Blockade and Identifies IL27RA as a Potential Target in Therapy‐Resistant Epithelial Cells

**FIGURE 1:**
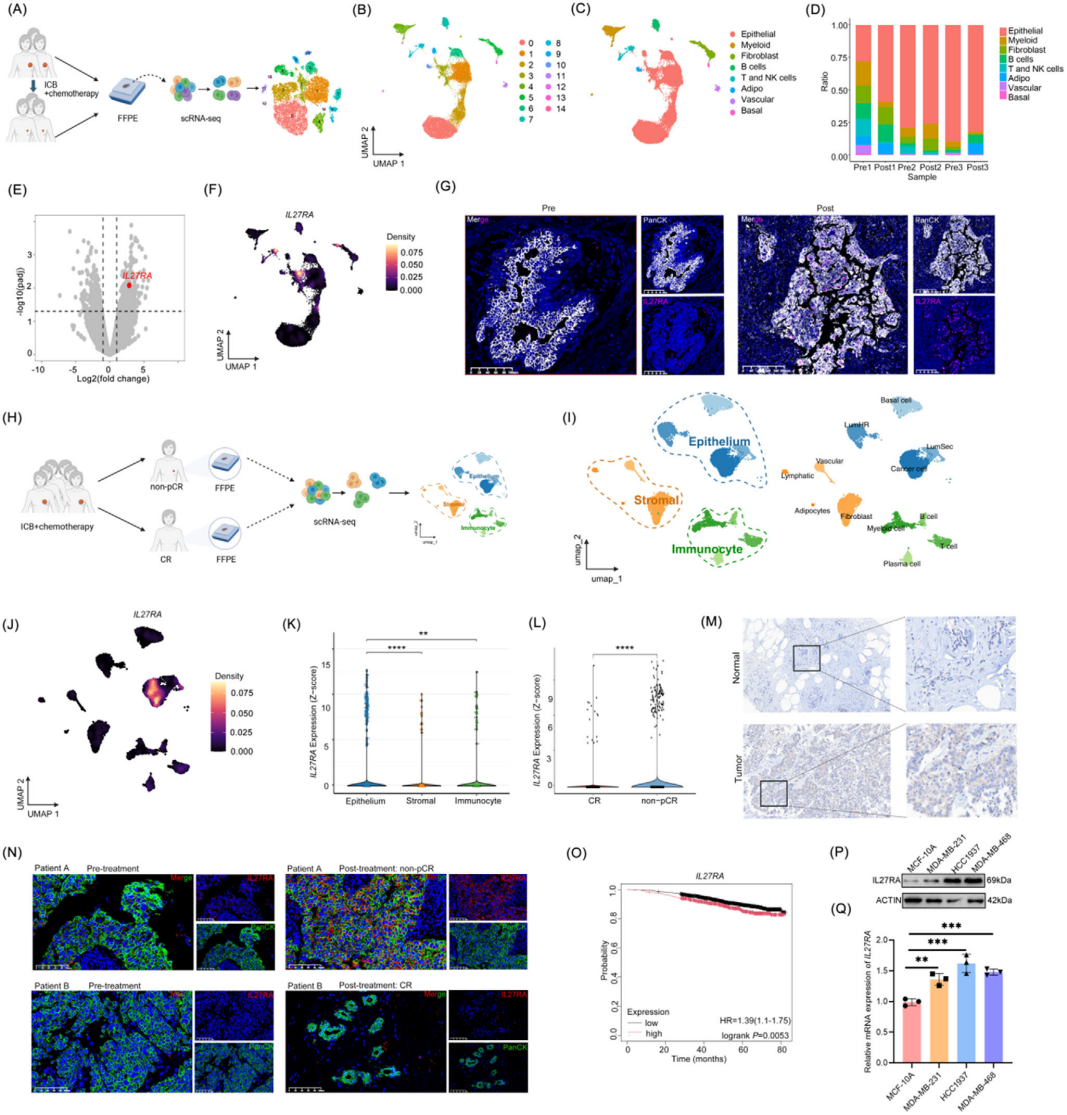
Integrated single‐cell transcriptomics and clinical analyses reveal IL27RA expression patterns and prognostic significance in TNBC receiving neoadjuvant ICB plus chemotherapy. (A) Schematic overview of the single‐cell RNA‐seq workflow using paired pre‐ and post‐treatment TNBC specimens (n = 3 pairs) from patients who failed to achieve pCR after neoadjuvant ICB plus chemotherapy. (B) Uniform manifold approximation and projection (UMAP) visualization showing the distribution of cell clusters after dimensionality reduction and unsupervised clustering. (C) UMAP plot displaying annotated cell types based on canonical marker genes. (D) Bar plot depicting changes in the proportions of major cell populations before and after treatment. (E) Volcano plot showing differentially expressed genes (DEGs) in epithelial cells between pre‐ and post‐treatment samples. (F) UMAP plot illustrating the expression density of *IL27RA* across cell populations. (G) Representative immunofluorescence images of PanCK and IL27RA staining in TNBC specimens before and after neoadjuvant therapy. (H) Workflow diagram for the single‐cell RNA‐seq analysis of CR (n = 3) and non‐pCR (n = 3) subgroups after ICB plus chemotherapy. (I) UMAP plots of breast tumor microenvironment cells, showing the major compartments (epithelial, stromal, immune; left) and the refined subclusters (right). (J) UMAP plot displaying *IL27RA* expression density across all cell types. (K) Violin plots showing Z‐score–normalized IL27RA expression in epithelial, stromal, and immune compartments. (L) Violin plots comparing Z‐score–normalized IL27RA expression in cancer cells between CR and non‐pCR subgroups. (M) Representative immunohistochemistry images of IL27RA staining in clinical TNBC samples, with boxed regions indicating magnified areas. (N) Representative immunofluorescence images of paired pre‐ and post‐treatment tumor samples from two TNBC patients (Patient A: non‐pCR; Patient B: CR). PanCK (green) marks tumor epithelial cells, IL27RA (red) indicates receptor expression, and DAPI (blue) stains nuclei. (O) Kaplan‐Meier survival curves for overall survival of TNBC patients with lower and higher IL27RA expression based on the KM Plotter database. (P and Q) qPCR and western blot analyses of IL27RA expression in major human TNBC cell lines compared with normal breast epithelial cells. Data are presented as mean ± SD. Statistical significance was determined by the Wilcoxon rank‐sum test (K and L), log‐rank test (O), and one‐way ANOVA (Q). *p < 0.05, **p < 0.01, ***p < 0.001, ****p < 0.0001. The schematic diagram was created using https://BioRender.com.

To investigate the mechanisms underlying the lack of pathological complete response in primary tumors from early‐stage TNBC patients treated with neoadjuvant chemo‐immunotherapy, we selected three patients who met the treatment criteria but failed to achieve pCR. The baseline characteristics of these patients are summarized in Table 1. FFPE biopsy samples were collected from the pretreatment lesions and the post‐treatment residual lesions, yielding a total of six samples. These samples were subjected to single‐cell RNA sequencing (Figure 1A). After filtering and quality control, 20 961 high‐quality cells were retained for downstream analysis. Batch correction, dimensionality reduction, and clustering identified 15 distinct cell clusters (labeled 0–14, Figure 1B). The distribution of these clusters in pretreatment (Pre) and post‐treatment (Post) samples is shown in Figure S1A. Based on canonical marker genes, the 15 clusters were annotated as epithelial cells (Epithelial), myeloid cells (Myeloid), fibroblasts (Fibroblast), B cells, T/NK cells, adipocytes (Adipo), vascular‐associated cells (Vascular), and basal cells (Basal) (Figure 1C, Figure S1B). In total, 17 224 cells were captured in the pretreatment samples and 3737 cells in the post‐treatment samples (Figure S1C). The proportional composition of cell types shifted after treatment, with most cell populations decreasing in abundance in the post‐treatment samples (Figure 1D).

**TABLE 1:** Clinicopathological Characteristics of Patients for Single‐Cell Sequencing.

Patient ID	Response Group	Age	TNM Stage (Pre‐treatment)	Miller & Payne Grade
P1	non‐pCR	44	T1N1M0	G2
P2	non‐pCR	47	T2N2M0	G2
P3	non‐pCR	50	T2N2M0	G1
P4	CR	44	T3N2M0	G5
P5	CR	52	T2N1M0	G5
P6	CR	64	T2N1M0	G5

To explore potential mechanisms of resistance to immunotherapy, we first performed a benign‐versus‐malignant assessment of all epithelial cells and confirmed that all epithelial clusters represented malignant breast cancer cells (Figure S1D). Differential expression analysis of epithelial cells before and after treatment identified 780 differentially expressed genes (DEGs), of which 442 were upregulated and 338 were downregulated in the Post group compared with the Pre group (Table S5). Notably, IL27RA was among the genes significantly upregulated after treatment (Figure 1E). In our previous work, a series of interleukin signaling pathways were implicated as key modulators of immunotherapy responses in breast cancer [29]. IL27RA has primarily been reported to function in immune cells, whereas its role in epithelial cells—particularly in breast cancer epithelium—remains poorly characterized [30]. Single‐cell analysis of IL27RA expression showed that IL27RA was highly expressed only in a subset of epithelial cells, T/NK cells, and a very small fraction of fibroblasts (Figure 1F). Consistently, mIHC demonstrated higher IL27RA expression in cancer epithelial cells from non‐responders after neoadjuvant therapy compared with pretreatment samples (Figure 1G). Further analysis revealed that in post‐treatment samples from non‐pCR patients, IL27RA expression in T cells was consistent with its previously characterized role in the immune compartment, whereas its expression in fibroblasts was only minimal. By contrast, IL27RA was markedly enriched in cancer epithelial cells, highlighting the importance of focusing on tumor cell–intrinsic IL27RA signaling (Figure S1E).

To further verify the association between high IL27RA expression in epithelial cells and resistance to immunotherapy, we additionally enrolled three early‐stage TNBC patients treated at our center who received the same neoadjuvant regimen as described above but achieved pCR (Figure 1H). The baseline characteristics of these patients are also presented in Table 1. These samples were used as a control cohort and jointly analyzed with the post‐treatment samples from the three non‐pCR patients. Cells were again classified into corresponding cell types (Figure 1I, Figure S1F,G). Strikingly, *IL27RA* remained highly expressed only in cancer epithelial cells from the non‐pCR group (Figure 1J–L). This difference was also evident when comparing TNBC tissues with normal breast tissues (Figure 1M). We further performed multiplex immunofluorescence staining on paired pre‐ and post‐treatment tumor specimens from additional TNBC patients treated with the same neoadjuvant regimen at our center. In non‐pCR patients, IL27RA expression was markedly increased in tumor cells (PanCK⁺) after treatment compared with baseline. By contrast, patients who achieved CR exhibited minimal residual tumor burden after therapy, and IL27RA expression remained nearly undetectable both before and after treatment (Figure 1N).

Survival analysis of breast cancer mRNA expression data from the Kaplan‐Meier Plotter database revealed that high *IL27RA* expression was significantly associated with worse overall survival (OS) (n = 2976, log‐rank *p* = 0.0053, Figure 1O). This adverse prognostic impact was further validated in an independent breast cancer cohort (Figure S1H). In addition, both IL27RA mRNA and protein levels were markedly higher in major human TNBC cell lines (MDA‐MB‐231, HCC1937, and MDA‐MB‐468) than in the normal breast epithelial cell line MCF10A (Figure 1P,Q). Collectively, these findings indicate that IL27RA represents a potential therapeutic target in breast cancer epithelial cells, particularly in the context of immunotherapy resistance.

### 3.2 IL27RA Does Not Independently Affect TNBC Cell Growth or Migration

After confirming the potential detrimental role of IL27RA in TNBC epithelial cells, we selected two TNBC cell lines—the murine PY8119 cell line and the human MDA‐MB‐231 cell line—for further investigation. Stable *IL27RA* (*Il27ra* in mice) knockdown and overexpression cell lines were generated via lentiviral transduction, and successful manipulation of IL27RA expression was validated by Western blotting (Figure S2A). Cell proliferation and migration assays showed that neither *IL27RA* overexpression nor knockdown altered the proliferative or migratory abilities of breast cancer cells (Figure S2B–I), which appeared inconsistent with the association between IL27RA expression and poor prognosis suggested by our single‐cell analysis in Section 1.

As a subunit of the IL‐27 receptor, IL27RA primarily functions through participation in the IL‐27 signaling pathway, which includes activation of downstream JAK–STAT signaling, particularly STAT3 (Figure S3A). To determine whether IL27RA may regulate breast cancer growth through this mechanism, we supplemented the *Il27ra*‐overexpressing PY8119 cell line with recombinant IL‐27 (rIL‐27) to mimic activation of the IL‐27 signaling axis in vitro. STAT3 phosphorylation was examined to confirm pathway activation (Figure S3B). Although rIL‐27 robustly induced STAT3 phosphorylation, it did not affect the proliferation of breast cancer cells (Figure S3C). We also considered the possibility that endogenous ligands within the tumor microenvironment might underlie the observed effects. Analysis of *IL27* expression in our single‐cell RNA‐sequencing dataset revealed that IL27 transcripts were detectable across multiple immune and stromal cell populations, including T cells, myeloid cells, and fibroblasts. In contrast, *IL27* expression was only sporadically observed in tumor epithelial cells, and no clearly defined *IL27*‐high epithelial subcluster was identified (Figure S3D). These results indicate that IL27RA‐mediated regulation of TNBC growth cannot be explained by direct, cell‐intrinsic effects of IL27RA or by simple activation of the IL‐27/IL27RA–JAK–STAT axis in tumor cells alone, and instead is likely to depend on additional components present within the tumor microenvironment.

To better recapitulate the in vivo context while minimizing potential influences from lymphocyte‐mediated immune surveillance, we performed orthotopic implantation of *IL27RA*‐overexpressing MDA‐MB‐231 cells into NOD/SCID mice. Given their profound immunodeficiency—characterized by the absence of functional T and B cells and defects in multiple innate immune compartments—NOD/SCID mice are considered an ideal model for assessing the intrinsic growth properties of malignant tumor cells [31]. Consistent with our in vitro findings, IL27RA overexpression did not alter tumor growth in vivo in this setting (Figure S3E–G), further supporting the notion that IL27RA does not intrinsically impact the growth or migration of TNBC cells. Together with our observations in immunocompetent mice described below, these data suggest that IL27RA does not primarily regulate tumor progression through direct modulation of cancer cell proliferation or migration. Instead, IL27RA likely exerts its protumor function by modulating immune cells within the tumor microenvironment, thereby shaping the balance between anti‐tumor and pro‐tumor immune responses and contributing to IL27RA‐associated immunotherapy resistance.

### 3.3 IL27RA Knockout‐mediated Tumor Suppression in Breast Cancer Depends on CD8⁺ T Cells

**FIGURE 2:**
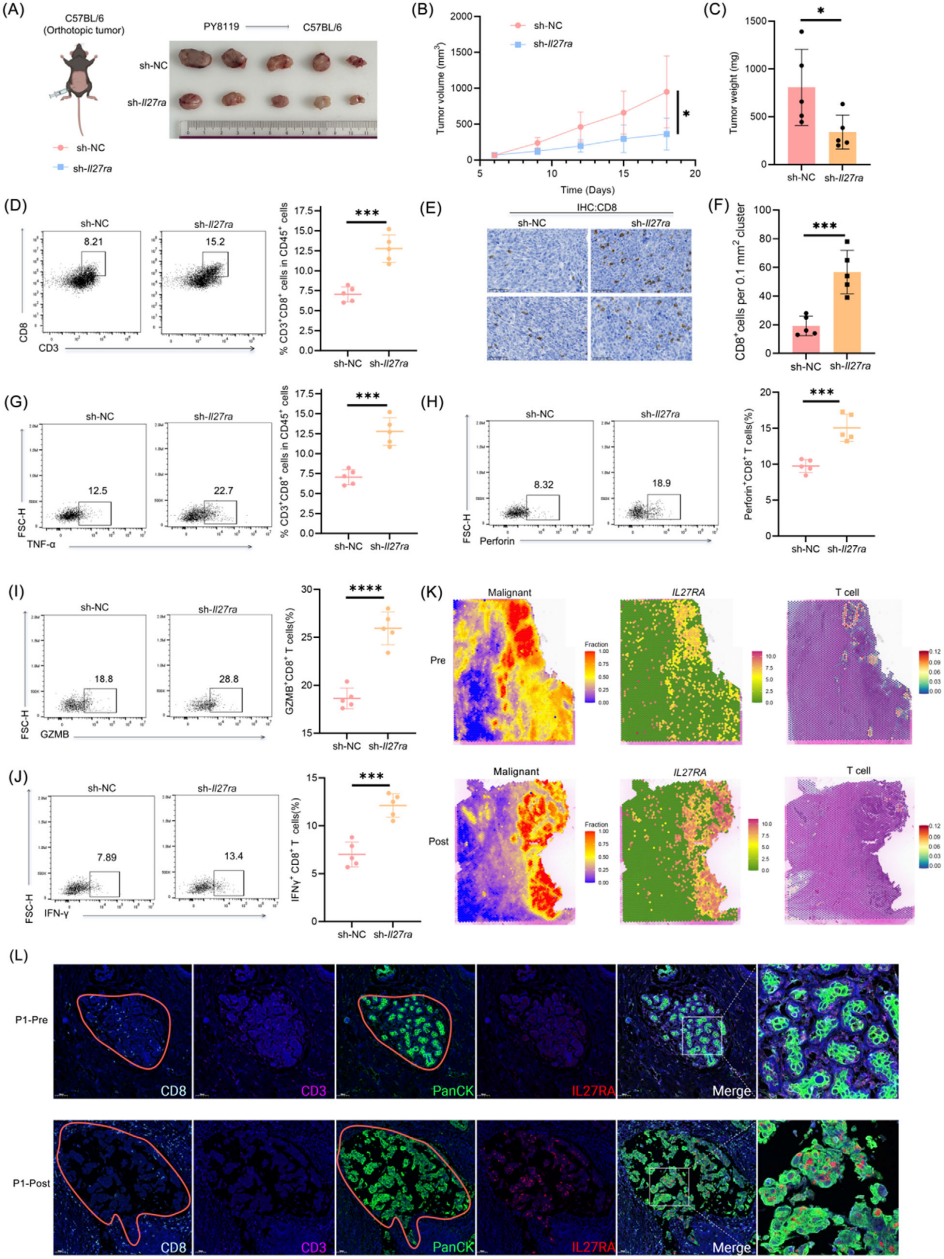
IL27RA knockout suppresses breast tumor growth and enhances CD8⁺ T cell responses. (A‐C) Study design and representative endpoint tumor images (A), tumor growth curves (B), and tumor weights at study termination (C) from orthotopic PY8119 models (n = 5). (D) Flow‐cytometry quantification of CD3⁺ CD8⁺ T cells Among CD45⁺ tumor‐infiltrating immune cells. Representative plots (left) and summary statistics (right) are shown (n = 5). (E and F) Representative IHC images of CD8⁺ T cell infiltration (E) and quantification of CD8⁺ T cell density normalized by tumor area (F), with each data point representing an independent tumor (n = 5). (G–J) Intracellular cytokine staining and flow cytometry analysis of TNF‐α (G), Perforin (H), GZMB (I), and IFN‐γ (J) production in CD8⁺ T cells isolated From PY8119.sh‐*Il27ra* or control tumors. Representative flow plots (left) and quantification (right) are shown (n = 5). (K) Spatial transcriptomics analysis of non‐pCR TNBC tissues pre‐ and post‐treatment, showing (From left to right) malignant‐region probability, *IL27RA* expression, and T cell spatial distribution. (L) Representative multiplex immunofluorescence images of CD8, CD3, PanCK, and IL27RA staining in pre‐ and post‐treatment samples, With outlined regions and magnified views shown on the right Data are shown as mean ± SD. Statistical significance was assessed by two‐way ANOVA With Sidak's multiple‐comparisons test for tumor growth curves (B) and by two‐tailed unpaired Student's *t*‐tests for tumor weights (C), CD8⁺ T cell frequencies (D), CD8⁺ T cell density (F), and cytokine production (G–J). *p < 0.05, **p < 0.01, ***p < 0.001. The schematic diagram was created using https://BioRender.com.

After confirming that IL27RA does not directly influence the growth or migration of breast cancer cells, we established orthotopic tumor models in C57BL/6J mice using PY8119.sh‐*Il27ra* stable cell lines. Knockout of Il27ra markedly suppressed excessive tumor growth (Figure 2A–C). We repeated the same experiment using EO771.sh‐*Il27ra* cells, and obtained consistent results (Figure S4A–D). These findings prompted us to further investigate how IL27RA expressed by breast cancer epithelial cells shapes the tumor microenvironment, particularly its impact on immune cell infiltration. Tumor‐infiltrating immune cells (CD45⁺) were isolated and analyzed via flow cytometry (Figure S4E,F). Compared with control tumors, *Il27ra*‐knockdown tumors exhibited a significantly increased proportion of infiltrating CD8⁺ T cells (Figure 2D), whereas the proportions of MDSCs, macrophages, NK cells, and CD4⁺ T cells remained unchanged (Figure S4G–L). Immunohistochemical analysis of tumor tissues further confirmed the enhanced CD8⁺ T cell infiltration associated with IL27RA loss (Figure 2E,F). Notably, these CD8⁺ T cells displayed significantly elevated expression of cytotoxic effector molecules, including TNF‐α, Perforin, Granzyme B (GZMB), and IFN‐γ (Figure 2G–J).

To determine whether the changes in CD8⁺ T cell infiltration and function are essential for the tumor‐suppressive effect of IL27RA knockout, we implanted MDA‐MB‐231.sh‐NC and sh‐*IL27RA* cells into T cell–deficient nude mice to generate both orthotopic tumors and lung metastasis models. In contrast to the striking differences observed in immunocompetent C57BL/6J mice, tumor volumes (Figure S5A–C) and survival in the lung metastasis model (Figure S5D,E) showed no significant differences between groups in nude mice. To further validate the role of CD8⁺ T cells in mediating the protective effects of Il27ra deficiency, we established a PY8119.sh‐*Il27ra* lung metastasis model in immunocompetent C57BL/6J mice and administered CD8⁺ T cell–neutralizing antibodies. To avoid off‐target effects of anti‐CD8a antibodies on natural killer T (NKT) cells, we used an anti‐CD8b antibody, with an IgG1 isotype antibody as control [32]. Neutralization of CD8⁺ T cells completely abolished the survival advantage conferred by *Il27ra*‐knockdown breast cancer cells (Figure S5F–I). Together, these results demonstrate that IL27RA knockout suppresses breast tumor progression in a CD8⁺ T cell–dependent manner.

To further delineate the spatial relationship between CD8⁺ T cells and IL27RA expression—particularly in the context of human TNBC immunotherapy—we performed spatial transcriptomic profiling on paired pre‐ and post‐treatment FFPE samples from one non‐pCR patient identified in Section 1, and obtained adjacent H&E‐stained sections (Figure S6A–F). As shown in Figure 2K, *IL27RA* expression was globally increased after neoadjuvant therapy and remained predominantly localized within malignant epithelial regions. Before treatment, T cells were readily detectable within tumor areas; however, in the post‐treatment sample, T cells were largely absent from regions with high *IL27RA* expression, and their overall distribution was markedly reduced across the tissue. Consistent with these findings, mIHC analysis showed markedly increased IL27RA expression along with prominent CD8⁺ T cell efflux in resistant FFPE samples (Figure 2L).

### 3.4 IL27RA Limits the Antigen‐Specific T Cell Anti‐Tumor Response

**FIGURE 3:**
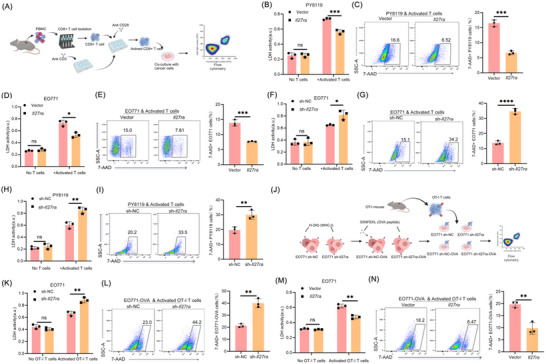
IL27RA restricts antigen‐specific T cell cytotoxicity. (A) Schematic of the in vitro CD8⁺ T cell activation workflow. (B–E) Co‐culture assays of PY8119 or EO771 cells transduced with vector or *Il27ra* overexpression constructs and activated CD8⁺ T cells. LDH release after 48 h (B, D) and flow‐cytometry quantification of 7‐AAD⁺ tumor cells (C, E) are shown. Wells without CD8⁺ T cells served as controls; a.u., arbitrary units. Left: Representative dot plots; right: Quantification. (F–I) Co‐culture assays using EO771 or PY8119 cells transduced with sh‐NC or sh‐*Il27ra*. LDH release (F, H) and 7‐AAD⁺ tumor‐cell frequencies (G, I) after 48 h are shown. (J) Schematic of the antigen‐specific OT‐I T cell cytotoxicity assay. (K–N) OT‐I mediated killing of EO771‐OVA cells transduced with sh‐NC or sh‐*Il27ra* (K, L) or vector or *Il27ra* overexpression constructs (M, N). LDH release (K, M) and 7‐AAD⁺ tumor‐cell frequencies (L, N) after 48 h co‐culture are shown. Wells without OT‐I T cells served as controls. All data are presented as mean ± SD, n = 3. Statistical significance was determined using two‐tailed unpaired Student's *t*‐tests. Ns, not significant; *p < 0.05, **p < 0.01, ***p < 0.001, ****p < 0.0001. The schematic diagram was created using https://BioRender.com.

To determine whether IL27RA enables cancer cells to evade CD8⁺ T cell–mediated cytotoxicity, we isolated CD8⁺ T cells from the spleens of C57BL/6J mice, activated them in vitro, and co‐cultured them with *Il27ra*‐knockout or *Il27ra*‐overexpressing mouse breast cancer stable cell lines. CD8⁺ T cell cytotoxicity was assessed by flow cytometry (Figure 3A). We observed that *Il27ra*‐overexpressing cancer cells were significantly more resistant to activated CD8⁺ T cell killing compared with control cells (Figure 3B–E). Conversely, *Il27ra*‐knockout cancer cells were more susceptible to CD8⁺ T cell–mediated cytotoxicity (Figure 3F–I).

To further verify whether CD8⁺ T cell killing of IL27RA‐manipulated tumor cells depends on antigen‐specific recognition, we pulsed the above‐mentioned stable cell lines with the OVA‐derived peptide 257–264 (SIINFEKL). This peptide binds to the H‐2K^b^ molecule (a murine MHC‐I allele) on EO771 cells, enabling them to present OVA antigens. CD8⁺ T cells were then isolated from the spleens of OT‐I transgenic mice, activated, and co‐cultured with the SIINFEKL‐loaded cancer cells to evaluate antigen‐specific cytotoxicity (Figure 3J). Both LDH release assays and flow cytometric analysis demonstrated a significantly increased proportion of dead tumor cells when *Il27ra* was knocked out (Figure 3K–N). These findings indicate that *Il27ra* overexpression restricts antigen‐specific CD8⁺ T cell antitumor activity.

### 3.5 IL27RA downregulates MHC‐I by Activating the PI3K/AKT Signaling Pathway in TNBC Cells

To investigate the mechanism through which IL27RA suppresses CD8⁺ T cell function in breast cancer, we first examined whether IL27RA regulates the expression of PD‐L1 (programmed death ligand‐1), which has been reported to participate in similar immunoregulatory processes in hepatocellular carcinoma [33]. Unexpectedly, neither knockdown nor overexpression of *IL27RA* altered PD‐L1 levels in TNBC cells (Figure S7A).

**FIGURE 4:**
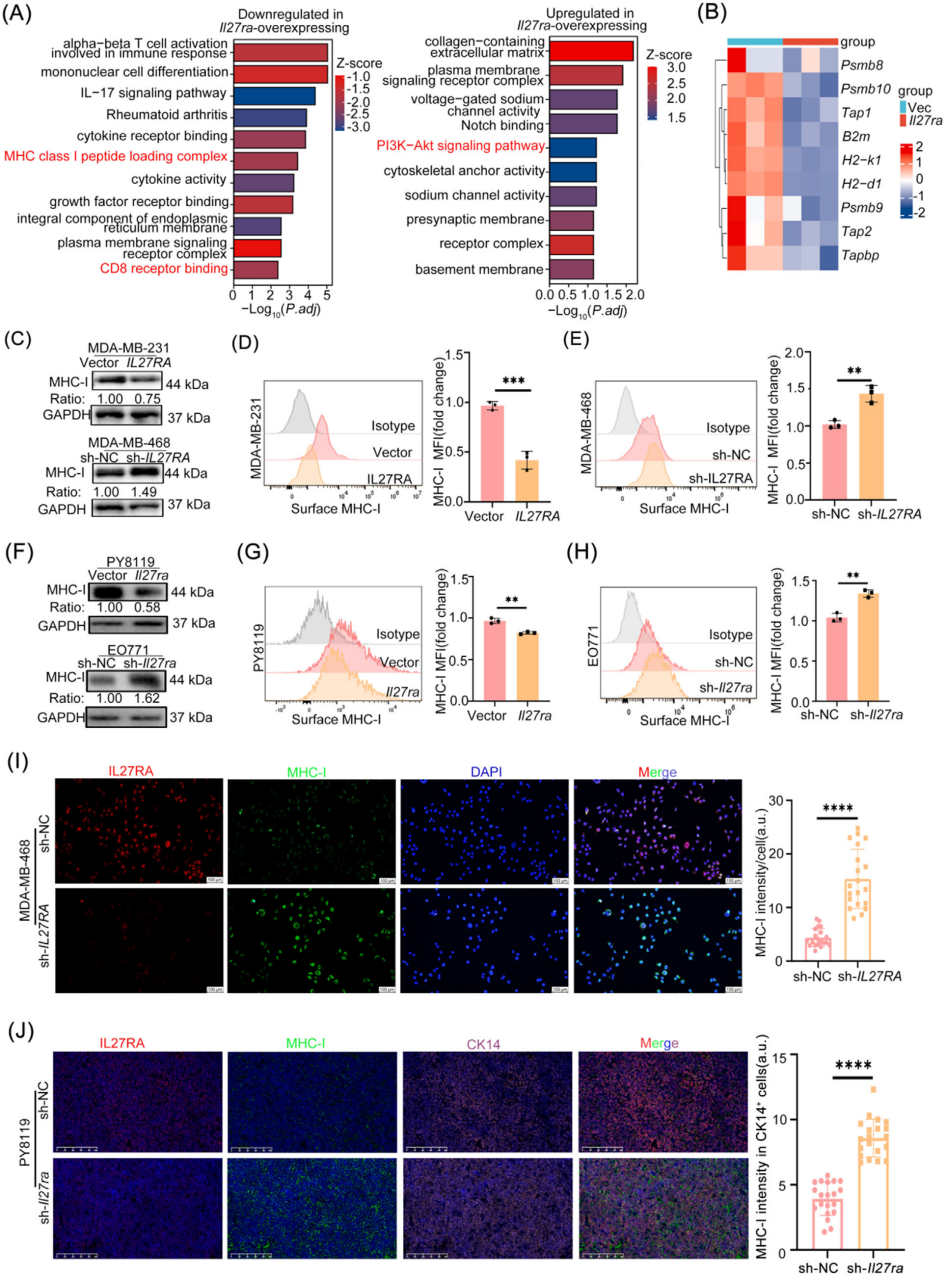
IL27RA suppresses MHC‐I expression in human and murine TNBC cells. (A) Top enriched pathways identified by combined GO/KEGG analysis of PY8119 cells overexpressing *Il27ra* compared with vector controls. (B) Heatmap showing the expression of antigen‐processing and presentation (APP)–related genes in *Il27ra*‐overexpressing PY8119 cells (red) versus control cells (blue). (C) Immunoblot analysis of MHC‐I and GAPDH (loading control, 37 kDa) in MDA‐MB‐231 cells overexpressing *IL27RA* (vector as control, n = 3) and MDA‐MB‐468 cells with *IL27RA* knockdown (sh‐NC as control, n = 3). (D) Flow‐cytometry analysis of surface MHC‐I mean fluorescence intensity (MFI) in MDA‐MB‐231 cells overexpressing *IL27RA* (n = 3). Representative histograms (left) and quantification (right; fold change relative to control) are shown. (E) Flow‐cytometry analysis of surface MHC‐I MFI in MDA‐MB‐468 cells with *IL27RA* knockdown (sh‐NC as control, n = 3). Representative histograms (left) and quantification (right) are shown. (F) Immunoblot analysis of MHC‐I and GAPDH in PY8119 and EO771 cells overexpressing Il27ra (vector as control, n = 3) or with *Il27ra* knockdown (sh‐NC as control, n = 3). (G) Flow‐cytometry quantification of surface MHC‐I MFI in PY8119 cells overexpressing *Il27ra* (n = 3). Representative histograms (left) and summary data (right) are shown. (H) Flow‐cytometry quantification of surface MHC‐I MFI in EO771 cells with *Il27ra* knockdown (sh‐NC as control, n = 3). Representative histograms (left) and summary data (right) are shown. (I) Immunofluorescence staining of IL27RA (red), MHC‐I (green), and DAPI (blue) in MDA‐MB‐468 cells with *IL27RA* knockdown (sh‐NC as control). The merged image is shown on the right, with quantification of MHC‐I fluorescence intensity per cell (a.u., arbitrary units). A total of 20 cells per group were analyzed across four independent experiments (5 cells per experiment). (J) Representative immunofluorescence images showing MHC‐I expression in CK14⁺ tumor cells and quantification of MHC‐I fluorescence intensity in CK14⁺ cells. A total of 20 CK14⁺ cells per group were analyzed across four independent experiments (5 cells per experiment); a.u., arbitrary units. Data are presented as mean ± SD. Statistical significance was determined using two‐tailed unpaired Student's *t‐*tests. **p < 0.01, ***p < 0.001, ****p < 0.0001. The schematic diagram was created using https://BioRender.com.

We then performed transcriptomic profiling of PY8119.Vector and PY8119.*Il27ra* cells. GO and KEGG enrichment analyses of differentially expressed genes revealed that *Il27ra* expression was closely associated with MHC‐I peptide loading complex formation, CD8 receptor binding, and the PI3K–AKT signaling pathway (Figure 4A). We further compared the expression of MHC‐I and several key antigen presentation pathway (APP) genes between the two groups. *Il27ra* overexpression significantly reduced MHC‐I and multiple essential APP gene transcripts in EO771 cells compared with vector controls (Figure 4B).

In many malignancies, high MHC‐I expression correlates with enhanced antigen presentation capacity and reduced immune evasion [34, 35]. In Section 3.4, we demonstrated that Il27ra affects the binding of murine MHC‐I to the OVA peptide and consequently impairs antigen‐specific antitumor immunity of OT‐I CD8⁺ T cells. These findings led us to hypothesize that IL27RA promotes tumor immune evasion by downregulating MHC‐I.

To test this hypothesis, we first assessed MHC‐I expression in TNBC overexpression cell lines. *IL27RA* overexpression resulted in decreased MHC‐I protein abundance, which was further confirmed by flow cytometry (Figure 4C–H). mIHC staining also supported the negative regulatory effect of IL27RA on MHC‐I expression (Figure 4I). Using PY8119‐derived tumors generated in vivo, we confirmed that IL27RA overexpression suppressed MHC‐I expression in tumor cells (Figure 4J).

To further elucidate the molecular mechanism underlying IL27RA‐mediated MHC‐I downregulation, we first examined the contribution of the IL‐27 ligand. Recombinant IL‐27 (rIL‐27) robustly induced STAT3 phosphorylation in IL27RA‐overexpressing PY8119 cells, confirming that the canonical IL‐27–IL27RA–JAK‐STAT signaling axis is functionally intact in this system. However, rIL‐27 stimulation did not reduce MHC‐I expression in the context of high IL27RA expression (Figure S7B,C). These findings suggest that IL27RA‐dependent suppression of MHC‐I is not mediated through the classical IL‐27–IL27RA–JAK‐STAT pathway, implicating alternative downstream signaling mechanisms.

**FIGURE 5:**
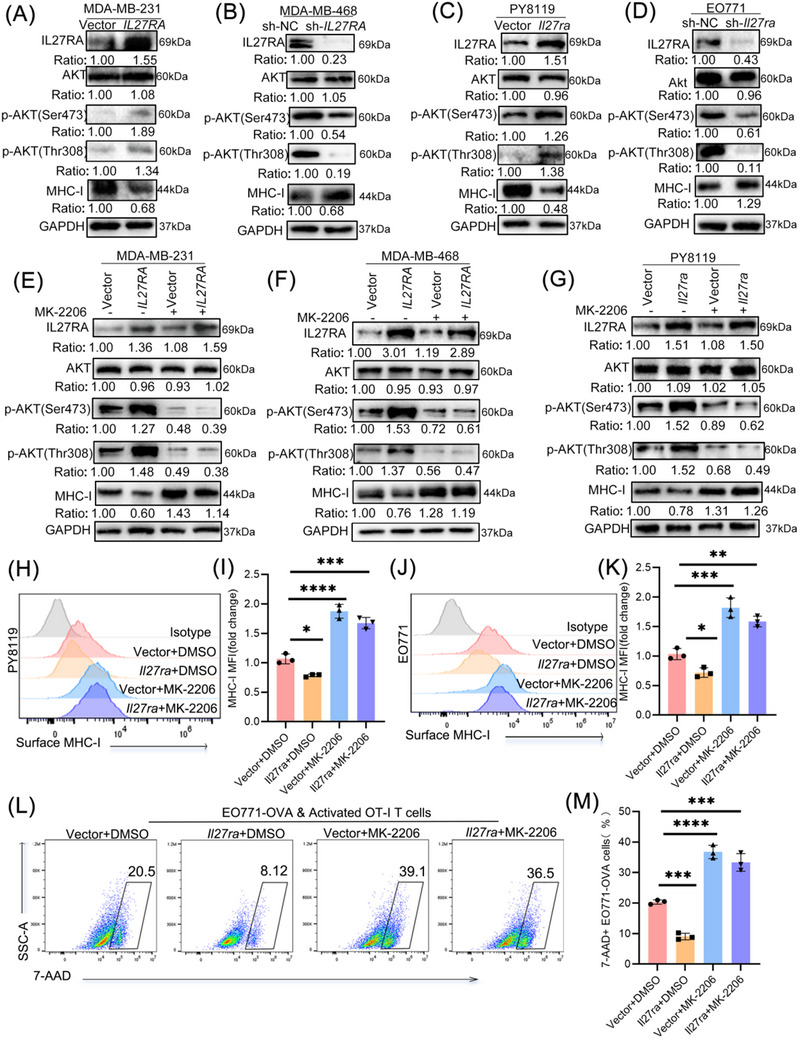
IL27RA downregulates MHC‐I in breast cancer cells through activation of the PI3K/AKT signaling pathway. (A, C) Immunoblot analysis of IL27RA, total AKT, p‐AKT (Thr308), p‐AKT (Ser473), and MHC‐I in MDA‐MB‐231 (A) and PY8119 (C) cells stably overexpressing *IL27RA* or *Il27ra*. GAPDH served as a loading control. (B and D) Immunoblot analysis of IL27RA, total AKT, p‐AKT (Thr308), p‐AKT (Ser473), and MHC‐I in MDA‐MB‐468 (B) and EO771 (D) cells with stable *IL27RA* or *Il27ra* knockdown (sh‐NC as control). (E–G) Immunoblot analysis of IL27RA, total AKT, p‐AKT (Thr308), p‐AKT (Ser473), and MHC‐I in MDA‐MB‐231 (E) and MDA‐MB‐468 (F) cells overexpressing *IL27RA*, and PY8119 (G) cells overexpressing *Il27ra*, cultured for 24 h with or without the AKT inhibitor MK‐2206. GAPDH served as a loading control. (H–K) Flow‐cytometry analysis of surface MHC‐I expression in PY8119 (H,I) and EO771 (J,K) cells following *Il27ra* overexpression or knockdown, with representative histograms (left) and quantification (right). (L,M) CFSE‐labeled EO771‐OVA cells (with or without *Il27ra* overexpression and with or without MK‐2206 treatment) were cocultured with activated OT‐I T cells for 24 h. Cytotoxicity was determined by quantifying the percentage of 7‐AAD⁺ cells within the CFSE⁺ population. Data are presented as mean ± SD, based on three independent biological replicates (n = 3). Statistical significance was determined using one‐way ANOVA with Tukey's multiple‐comparisons test. **p < 0.01, ***p < 0.001, ****p < 0.0001.

Building on this premise, and guided by transcriptomic enrichment analyses, we next evaluated activation of the PI3K/AKT pathway in MDA‐MB‐231 and PY8119 stable cell lines. IL27RA overexpression induced AKT phosphorylation at both Ser473 and Thr308, accompanied by a marked reduction in MHC‐I protein abundance (Figure 5A–D, Figure S7D–G). Pharmacologic inhibition of AKT with MK‐2206 effectively reversed IL27RA‐induced AKT activation and restored MHC‐I expression (Figure 5E–K), while also rescuing the antigen‐specific cytotoxic activity of OT‐I CD8^+^ T cells against breast cancer cells (Figure 5L,M). Together, these data establish PI3K/AKT signaling as a critical mediator of IL27RA‐driven immune evasion.

Collectively, these findings demonstrate that IL27RA downregulates MHC‐I expression through activation of the PI3K/AKT signaling pathway, thereby enabling breast cancer cells to evade CD8⁺ T cell–mediated immune surveillance.

### 3.6 Targeting IL27RA Exerts Dual Effects on Cancer Cells and the TME, Influencing the Development of TNBC

**FIGURE 6:**
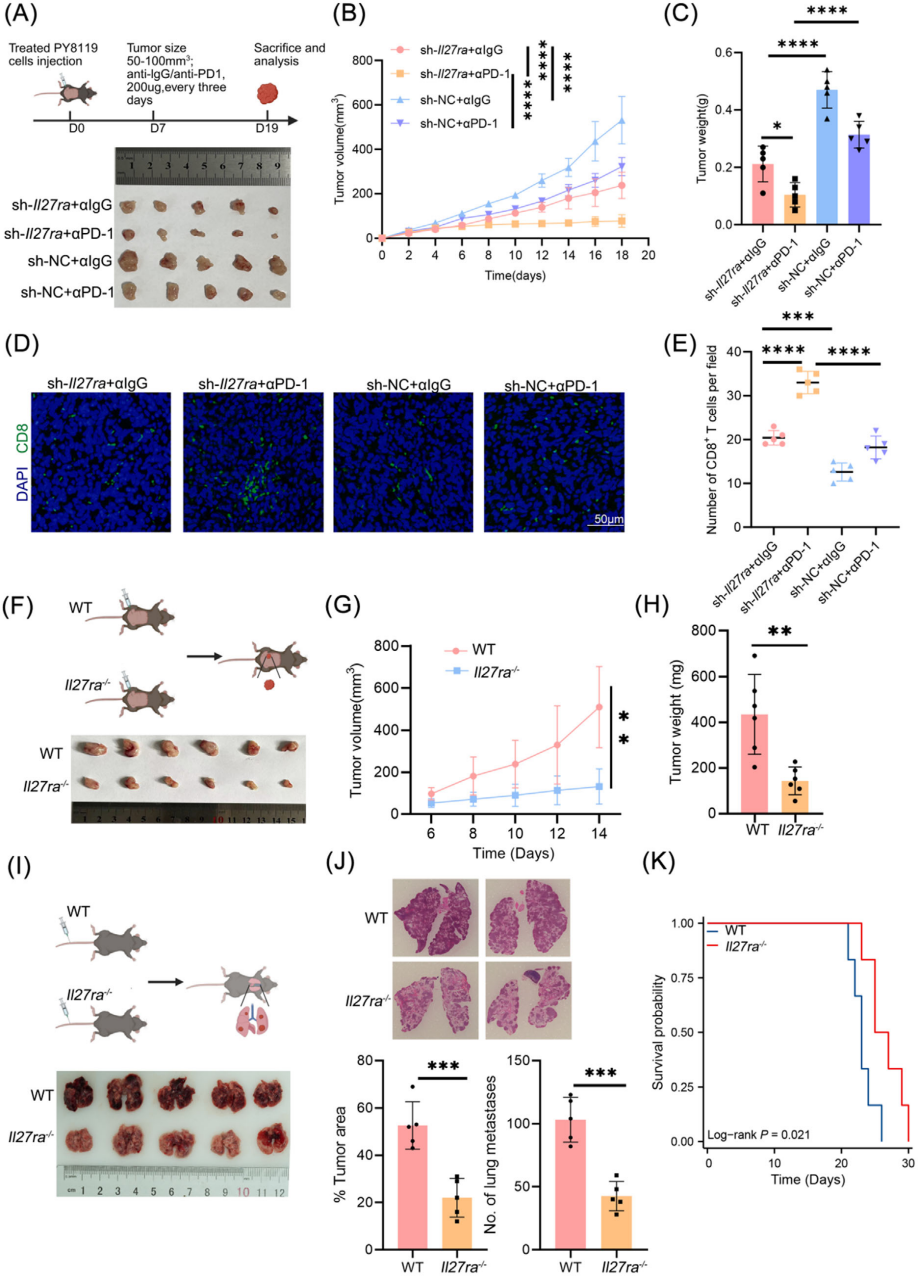
Targeting IL27RA exerts dual effects on tumor cells and the tumor microenvironment, suppressing TNBC progression. (A–C) Study design (A), tumor growth curves (B), and endpoint tumor weights (C) From orthotopic PY8119 models (n = 5). (D, E) Representative immunofluorescence images of CD8⁺ T cell infiltration (D, n = 5) and quantification of CD8⁺ T cell numbers per field (E). Scale bar, 50 µm. (F–H) Study design (F), subcutaneous tumor growth curves comparing wild‐type and *Il27ra^−/−^* mice (G), and corresponding endpoint tumor weights (H) (n = 6). (I) Experimental schematic and gross lung photographs showing metastatic burden (n = 5). (J) H&E‐stained lung sections and quantification of tumor area and metastatic lesion numbers (n = 5). (K) Kaplan‐Meier survival curves of wild‐type versus *Il27ra^−/−^* mice bearing PY8119 tumors (n = 6). Data are presented as mean ± SD. Statistical significance was determined by two‐way ANOVA (B,G), log‐rank test (K), two‐tailed unpaired Student's *t*‐test (H,J), and one‐way ANOVA (C,D). Ns, not significant; *p < 0.05; **p < 0.01; ***p < 0.001; ****p < 0.0001. The schematic diagram was created using https://BioRender.com.

To enhance the clinical applicability of IL27RA‐targeted therapy, we developed a combined treatment model that integrates *Il27ra* knockout with PD‐1 blockade. First, we simulated neoadjuvant immunotherapy for breast cancer using *Il27ra* knockout TNBC stable cell lines and established orthotopic tumors in immunocompetent C57BL/6J mice. Subsequently, the mice were treated with either αPD‐1 or IgG. The results demonstrated that *Il27ra* ablation effectively inhibited tumor growth and synergized with αPD‐1 therapy (Figure 6A–C). To explore changes in CD8⁺ T cells in the TME following this combined treatment, we prepared paraffin sections of tumor samples and performed immunofluorescence staining. The analysis confirmed that *Il27ra* deletion increased the infiltration of CD8⁺ T cells in the TME (Figure 6D,E).

However, generally speaking, checkpoint blockade therapy is not tumor‐specific and may impact the entire TME or even the systemic environment. Therefore, we aimed to analyze the broader changes in the TNBC microenvironment following *IL27RA* deletion and assess its potential effects on TNBC. Using CRISPR/Cas9 technology, we generated *Il27ra* knockout (KO) mice in a C57BL/6J background, with C57BL/6J wild‐type (WT) mice as controls. We then implanted orthotopic tumors in both groups to simulate TME alterations caused by *Il27ra* deletion. To our surprise, compared to WT mice, KO mice exhibited significantly restrained primary breast tumor development (Figure 6F–H). Furthermore, the lung metastasis model showed that KO mice had fewer lung metastases and longer survival times compared to WT mice (Figure 6I–K). These findings suggest that *Il27ra* deletion not only impacts the tumor cells but also alters the TME, thereby contributing to the suppression of breast cancer progression.

### 3.7 IL27RA Deficiency Drives T Cell Expansion and Enhanced Chemotactic Communication in Triple‐Negative Breast Cancer, Activating Anti‐Tumor Immune Responses

To explore why the TME lacking IL27RA suppresses TNBC growth, we revisited previous single‐cell RNA sequencing data. These results revealed that, apart from some epithelial cells, a subset of NK/T cells also exhibited high IL27RA expression (Figure 1F). We also analyzed two additional publicly available human TNBC datasets, which showed a similar trend (Figure S8A–D). Notably, IL27RA expression was primarily concentrated in specific lymphocytes, consistent with previous literature reports [30].

**FIGURE 7:**
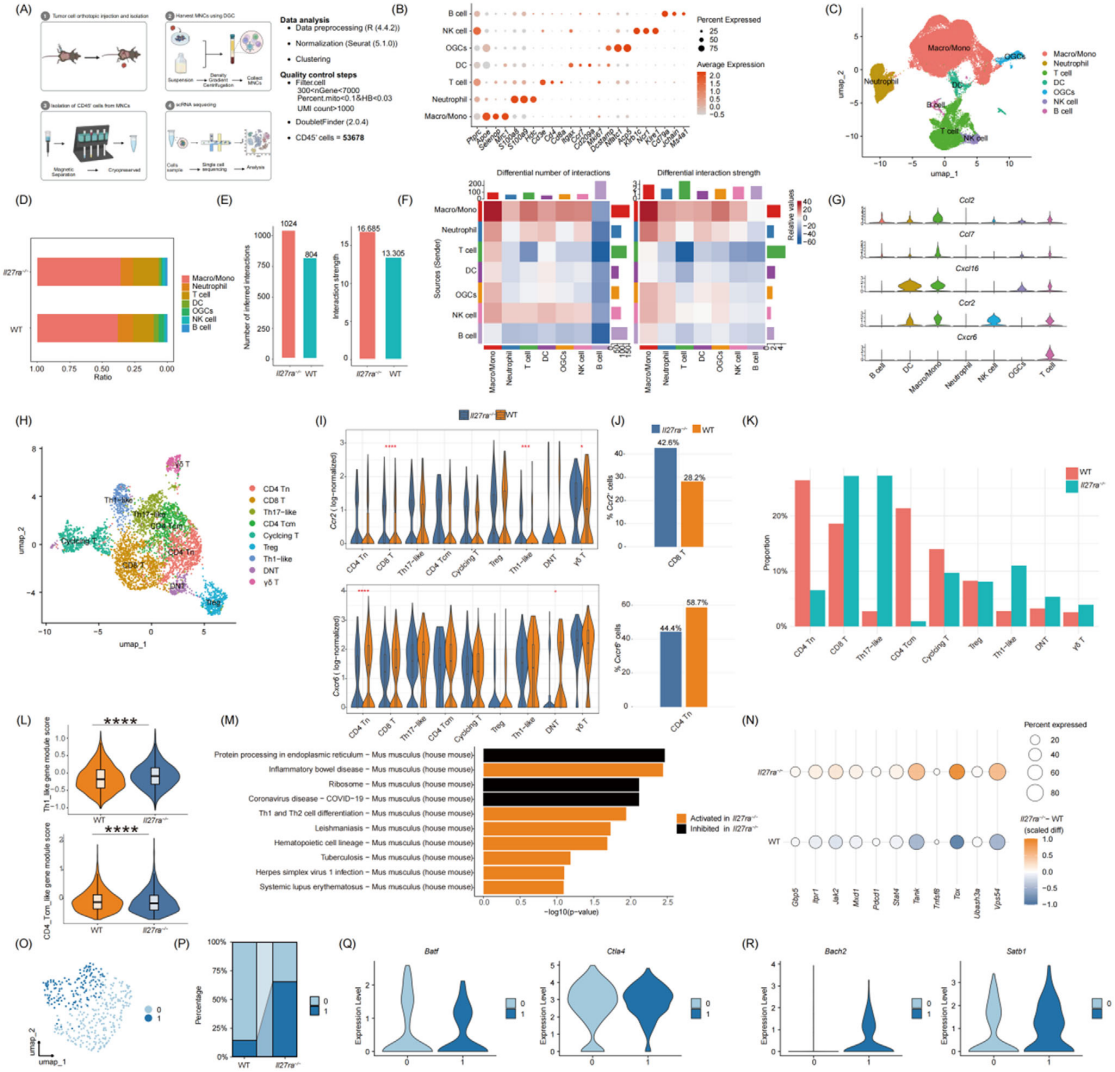
Single‐cell atlas reveals IL27RA deficiency reshapes the TNBC tumor microenvironment and drives T cell lineage reprogramming (A) Schematic overview of the single‐cell RNA‐seq workflow. (B) Bubble plot showing the expression patterns of selected genes across major immune cell populations, including B cells, NK cells, osteoclast‐like giant cells (OGCs), dendritic cells (DCs), T cells, neutrophils, and macrophage/monocyte populations (Macro/Mono). (C) UMAP visualization of annotated immune‐cell subclusters from WT and *Il27ra^−/−^* mice. (D) Bar plot displaying the proportions of major immune cell types in tumors from WT and *Il27ra^−/−^* mice. (E) Quantification of the number and overall strength of inferred cell‐cell interactions among immune populations in WT versus *Il27ra^−/−^* mice. (F) Heatmap summarizing differences in ligand‐receptor interaction counts and interaction strengths between annotated immune‐cell types in WT and *Il27ra^−/−^* mice. (G) Violin plots showing normalized expression of selected chemokines and their receptors across annotated immune‐cell subsets. (H) UMAP visualization of T cell subclusters annotated based on canonical marker genes. (I) Violin plots comparing normalized expression of Ccr2 (top) and Cxcr6 (bottom) across T cell subsets From *Il27ra^−/−^* and WT mice. (J) Bar plots showing the proportion of Ccr2⁺ CD8⁺ T cells (top) and Cxcr6⁺ CD4 Tn cells (bottom) in *Il27ra^−/−^* versus WT mice. (K) Bar plot showing the proportional distribution of major T cell subsets in WT and *Il27ra^−/−^* tumors. (L) Violin plots depicting Th1‐like gene module scores (top) and CD4 Tcm gene module scores (bottom) in WT versus *Il27ra^−/−^* mice. (M) KEGG pathway enrichment analysis of differentially expressed genes in T cell subsets from *Il27ra*‐deficient tumors. (N) Bubble plot showing relative expression levels of apoptosis‐related genes. (O) UMAP visualization of reclustered tumor‐infiltrating CD4⁺ Treg cells from WT and *Il27ra^−/−^* mice. (P) Stacked bar plot illustrating changes in proportions of Treg subclusters between WT and *Il27ra^−/−^* groups. (Q,R) Violin plots comparing normalized expression of key Treg‐regulatory genes (Ccr7, Batf, Bach2, Satb1) between WT and *Il27ra^−/−^* mice. Statistical significance (*p < 0.05; **p < 0.01; ***p < 0.001; ****p < 0.0001) was determined using the Wilcoxon rank‐sum test (I and L). The schematic diagram was created using https://BioRender.com.

To model the impact of IL27RA deletion on the immune microenvironment of breast cancer, we implanted PY8119 cells into *Il27ra* knockout and wild‐type mice for orthotopic tumor formation experiments. Tumor samples were collected from three different mice per group, and infiltrating immune cells (CD45⁺) were isolated for 10× single‐cell RNA sequencing (Figure 7A). Following the data filtering and processing workflow outlined in Section 3.1, dimensionality reduction and clustering analysis revealed 15 distinct cell subclusters (labeled 0–14, Figure S9A). The integration across different samples within the same group was robust, while large differences were observed between groups (Figure S9B,C). We identified marker genes for each cell type, categorizing the major immune cell types, including macrophages/monocytes, osteoclast‐like giant cells, neutrophils, T cells, dendritic cells, natural killer (NK) cells, and B cells (Figure 7B,C, Figure S9D). Among these, myeloid macrophages were the most abundant, followed by T cells (Figure 7D). Using the cell‐cell communication network from the scRNA‐seq data, we observed that the number and strength of cell interactions were significantly higher in the KO group compared to the WT group (Figure 7E), suggesting that *Il27ra* deletion enhances the overall signaling network among CD45⁺ immune cells, highlighting its important role in maintaining immune cell communication and anti‐tumor immune activity.

Given that T cells and NK cells are the primary populations expressing IL27RA, we focused on analyzing the changes in these cell types within the immune network following *Il27ra* deletion. The results showed that *Il27ra* deletion increased the proportions of T cells and NK cells in the TME (Figure S9E). Additionally, we observed that in the KO group, only macrophages/monocytes showed enhanced ligand‐receptor communication strength with T cells and NK cells (Figure 7F), suggesting that these cells play a critical bridging role in the immune regulatory network.

To further investigate the molecular basis driving the enhanced communication, we constructed a chemokine ligand‐receptor network (Figure S9F–H). We found that *Ccl2*, *Ccl7*, and *Cxcl16* were highly expressed in macrophages/monocytes, while their corresponding receptors, *Ccr2* and *Cxcr6*, were predominantly expressed in NK cells and T cells (Figure 7G). To identify the specific T cell subpopulations affected by *Il27ra* deletion, we performed sub‐clustering analysis of T cells and annotated them based on known marker genes (Figure 7H, Figure S9I–L). The analysis revealed that in the KO TME, *Ccr2* expression was elevated in several T cell subpopulations, most notably in CD8⁺ T cells, while *Cxcr6* expression decreased in CD4⁺ naïve T cells (Figure 7I,J). Consistent with this, we observed an increase in CD8⁺ T cells and a decrease in CD4⁺ naïve T cells (Figure 7K). These results suggest that IL27RA signaling plays a multi‐directional regulatory role in modulating T cell subpopulation chemotaxis and tissue homing, thereby reshaping the TME T cell subset composition.

To analyze the specific changes in T cells following *Il27ra* deletion, we re‐examined the proportions of T cell subpopulations and found that in the KO group, the proportion of CD4 Th1/17‐like cells increased, while CD4 Tcm (central memory) cells decreased (Figure 7K). Additionally, the KO group exhibited higher Th1‐like scores and lower CD4 Tcm scores (Figure 7L). KEGG pathway analysis showed that in Th1‐like CD4⁺ T cells, the KO group was enriched in several inflammatory and immune‐related KEGG pathways, including those promoting Th1/Th2 differentiation (Figure 7M). These pathways were enriched with typical Th1 and IFN‐γ modules, suggesting that Th1‐like cells in the KO group exhibited enhanced effector differentiation and immune activation. Meanwhile, pathways related to cellular stress, such as “Protein processing in endoplasmic reticulum” and “Ribosome,” were significantly suppressed in the KO group, indicating that IL27RA deletion may reduce the metabolic and folding stress in Th1‐like cells, helping them maintain a more favorable immune effector state. This differentiation activation promotes the mobilization of the CD4 Tcm pool to support downstream effector arms and tumor‐specific responses. Additionally, in the KO group, the expression of apoptosis‐related genes in CD4 Tcm cells was significantly decreased (Figure 7N). We also observed a slight expansion of a subpopulation of Tregs, designated Treg_1, in the KO group (Figure 7O,P), with a shift from stable to unstable anti‐tumor immune suppression (Figure 7Q,R). In conclusion, *Il27ra* deletion significantly enhances T cell expansion and chemotactic communication, activating the anti‐tumor immune response by remodeling the TME of TNBC.

### 3.8 *Il27ra* Deletion Enhances NK Cell Cytotoxic Maturation and Favors Tumor Tissue Residency in Triple‐Negative Breast Cancer

**FIGURE 8:**
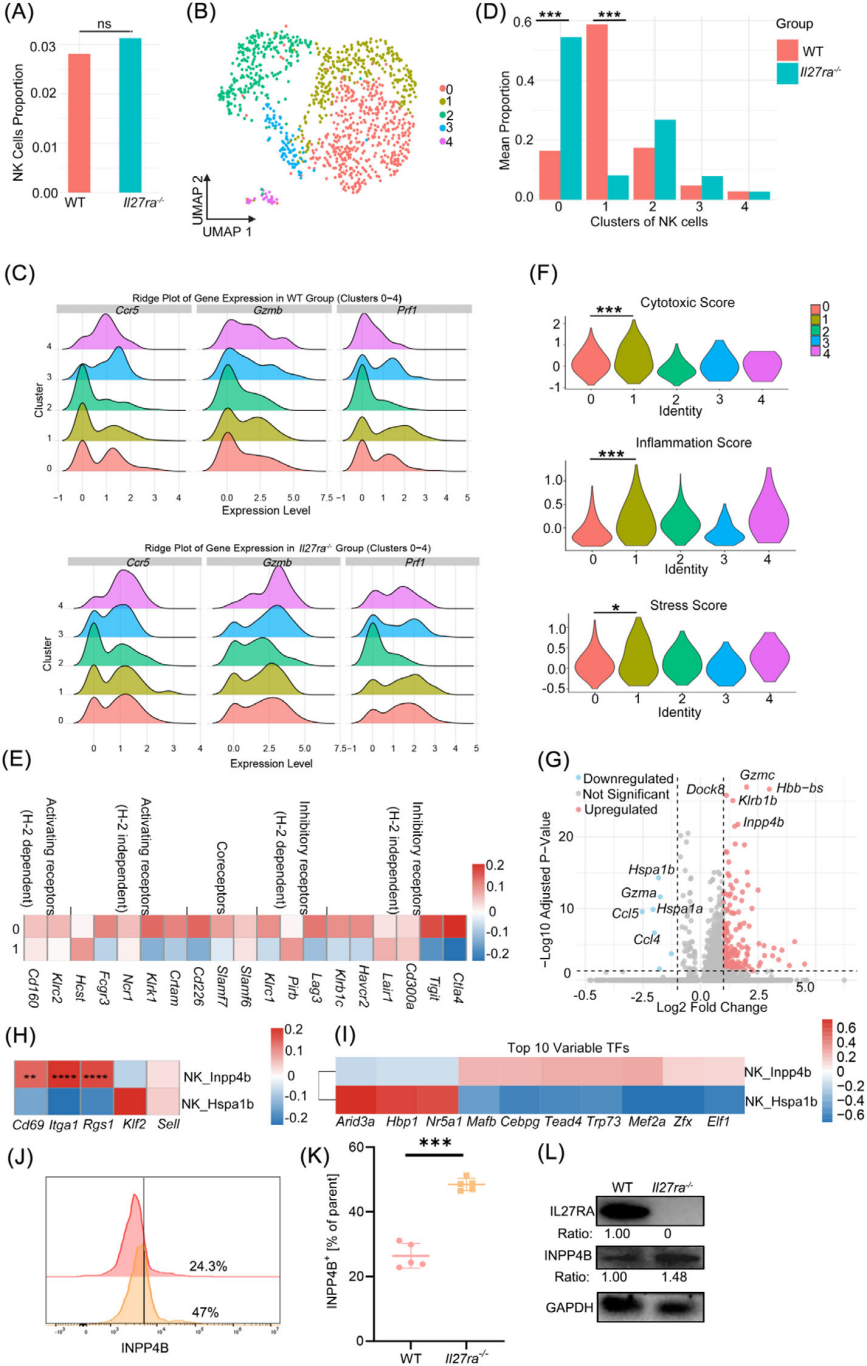
IL27RA deficiency reshapes the phenotype and functional maturation of tumor‐infiltrating NK cells in TNBC. (A) Bar plot showing the proportion of NK cells in tumors From *Il27ra^−/−^* and WT mice. (B) UMAP visualization of NK‐cell subclusters following reclustering and dimensionality reduction. (C) Ridge plots comparing the expression of key cytotoxicity‐ and recruitment‐related genes in NK cells From WT (top) and KO (bottom) tumors. (D) Bar plot showing the distribution of NK‐cell subsets in WT versus *Il27ra^−/−^* mice. (E) Heatmap displaying the expression of activating and inhibitory receptors across major NK‐cell subsets. (F) Violin plots comparing cytotoxicity, inflammation, and stress module scores between NK subset 0 and subset 1. (G) Volcano plot showing differentially expressed genes between NK subset 0 and subset 1, with the top five upregulated genes in each direction highlighted. (H) Heatmap comparing the expression of tissue‐residency‐associated genes between NK subset 0 and subset 1. (I) Heatmap showing inferred key transcription factors distinguishing NK subset 0 from subset 1. (J–L) Western blot analysis of INPP4B protein expression in tumor‐infiltrating NK cells isolated from WT and *Il27ra^−/−^* mice. Data for bar plots and dot plots represent biological replicates, with each mouse counted as one replicate, and are presented as mean ± SD. Group comparisons were performed using two‐tailed unpaired Student's *t*‐tests. For single‐cell RNA‐seq analyses, gene‐expression comparisons and module score comparisons Between NK subsets were assessed using two‐sided Wilcoxon rank‐sum tests. Differentially expressed genes were identified using Seurat's Wilcoxon method With Benjamini‐Hochberg correction. Statistical significance: Ns, not significant; *p < 0.05; **p < 0.01; ***p < 0.001; ****p < 0.0001.

We further assessed the impact of *Il27ra* deletion on tumor‐infiltrating NK cells. There were no significant differences in the overall NK cell population between the two groups (Figure 8A). We then subdivided the NK cells into five subpopulations, with functional and maturation status among the subgroups (Figure 8B, Figure S10A–C). In the KO group, representative cytotoxicity‐associated genes such as *Gzmb*, *Prf1*, and the chemokine receptor *Ccr5* were upregulated. This upregulation was widespread, with significant shifts to higher expression of these molecules across all subpopulations, especially *Gzmb* (Figure 8C). These results indicate that tumor‐infiltrating NK cells in KO mice exhibit significant functional differences compared to those in WT mice.

We further observed notable differences in the distribution of NK cell subpopulations between samples, with Subpopulation 0 being significantly enriched in the KO group and Subpopulation 1 being enriched in the WT group (Figure 8D). Therefore, we focused our analysis on these two subpopulations, as they represent the most characteristic features of the two groups. Interestingly, there were no significant differences in NK cell maturity between Subpopulations 0 and 1 (Figure S10C). Analysis of the expression patterns of activation and inhibitory receptors revealed that NK_0 cells exhibited a paradoxical activation profile, with a high expression of both activating receptors and inhibitory receptors (Figure 8E). We designed three gene combinations (Section 2.28) to assess cytotoxicity, inflammation, and stress levels in these subpopulations. The analysis showed that, compared to NK_0 cells, NK_1 cells exhibited higher toxicity, inflammation, and stress scores (Figure 8F). This difference is likely due to stronger immune signaling in NK_1 cells, leading to more intense activation of cytotoxic markers and changes in inflammation and stress.

Further differential gene expression analysis between the two groups identified 166 genes significantly upregulated in NK_0 cells and 7 genes significantly upregulated in NK_1 cells. The top five genes significantly expressed in each subgroup are shown in Figure 8G. Based on these gene characteristics, we designated NK_0 cells as NK_Inpp4b and NK_1 cells as NK_Hspa1b. *Inpp4b* is known to promote NK cell tissue residence [33]. Therefore, we further examined other known residence‐associated genes highly expressed in NK_Inpp4b cells and found that *Cd69*, *Itga1*, and *Rgs1* were significantly more expressed, although they were not included in the list of significant genes due to not meeting the log2FC inclusion criterion. In contrast, migration‐associated factors such as *Klf2* and *Sell* were more highly expressed in NK_Hspa1b cells (Figure 8H). This difference suggests that after *Il27ra* depletion, NK cells exhibit a stronger tendency for tumor residence. Transcription factor prediction results also indicated that NK_Hspa1b cells were more aligned with stress response and inflammation‐related transcriptional regulation, while NK_Inpp4b cells exhibited stronger tissue residency and maturation‐related transcriptional features (Figure 8I).

Pseudotime trajectory analysis identified two potential differentiation pathways for NK cells. Both trajectories started from the NK_Hspa1b subpopulation and progressed toward a more mature state (Figure S10D). Importantly, in the KO mice tumors, the initial recruitment of NK cells was reduced, leading to an increase in the progression of NK cells to more mature subpopulations. Pseudotime ordering showed that NK cells indeed differentiated toward more mature cytotoxic effector cells, characterized by upregulated cytotoxic genes (*Gzmb*, *Prf1*, and *Gzmc*) and downregulated inhibitory receptors (*Klrc1*, *Klrc2*, *Klre1*) (Figure S10E). Notably, the expression of *Gzmb* and *Gzmc* peaked later in pseudotime, suggesting their key roles in NK cell differentiation. Collectively, these data indicate that *Il27ra* deletion enhances NK cell activation, cytotoxic function, and terminal differentiation—highlighting its critical regulatory role in NK cell biology within the tumor microenvironment.

Finally, we further experimentally analyzed NK cells in the orthotopic breast tumors of the two mouse groups. Although there was no statistically significant difference in NK cell infiltration between the two groups (Figure S10F), functional analysis showed that NK cells from KO mice tumors exhibited stronger effector functions. Specifically, cytotoxic‐related molecules, such as the degranulation marker CD107a and Perforin, were significantly upregulated (Figure S10F–H). Furthermore, we observed a significant increase in INPP4B expression, indicating its role in regulating NK cell activation (Figure 8J–L).

In conclusion, this study provides detailed transcriptomic insights into tumor‐infiltrating NK cells following *Il27ra* knockout therapy. We analyzed NK cell subpopulations at a finer resolution, rather than treating them as a single group. We examined the molecular differences between NK subpopulations, hypothesized potential functional differences, and clarified the impact of *Il27ra* depletion on immune responses.

## 4 Discussion

Immunotherapy resistance has emerged as a major contributor to poor prognosis in breast cancer, particularly in patients with TNBC, yet its underlying molecular mechanisms remain incompletely understood [34, 35]. Increasing evidence suggests that bidirectional interactions between tumor cells and immune or stromal components of the TME play a decisive role in shaping therapeutic response and the development of resistance [36, 37]. In this study, by integrating single‐cell RNA sequencing of residual TNBC lesions before and after neoadjuvant chemotherapy combined with PD‐1 blockade, together with spatial transcriptomics, functional assays, and treatment simulation models, we systematically delineated the cellular landscape and molecular pathways associated with immunotherapy resistance. We identified IL27RA as a key driver of therapy‐resistant cancer epithelial cells.

The roles of interleukins and their receptors in tumor immunity are highly context dependent. On the one hand, the IL‐27/IL27RA axis has been reported to exert antitumor activity in several cancers by enhancing the effector functions of T cells and NK cells; on the other hand, emerging evidence suggests that IL‐27 may promote immune evasion under certain conditions [38]. A recent study by Chen et al., based on bulk transcriptomic data of breast cancer, reported that high IL27RA expression correlated with favorable prognosis, suggesting that IL27RA may serve as a protective factor in breast cancer [39]. These findings appear to contradict our observation that high IL27RA expression in TNBC cancer epithelial cells indicates immune escape and poor clinical outcome. We believe that this discrepancy can be largely attributed to several factors. First, IL27RA signals detected in bulk datasets predominantly originate from immune cells, making it difficult to disentangle the contribution from tumor epithelium. The prognostic association in bulk data likely reflects the antitumor roles of IL27RA⁺ T cells, NK cells, and dendritic cells. Second, immunosuppressive factors such as IL‐10 and TGF‐β, as well as varying levels of immune infiltration, may reshape IL‐27/IL27RA‐mediated regulation of HLA/MHC molecules; thus, the direction of IL27RA–HLA correlations may differ across immune contexts. Third, our cohort primarily consists of TNBC, a subtype characterized by a more immunosuppressive microenvironment, whereas the cohort analyzed by Chen et al. included multiple molecular subtypes. Differences in subtype composition and disease stage likely influence the prognostic significance of IL27RA. Taken together, IL27RA exhibits substantial context‐dependent functions across distinct cellular populations and immune niches. Our study complements and extends previous findings by uncovering a tumor‐intrinsic role of IL27RA in promoting immune evasion within cancer epithelial cells.

At the mechanistic level, we demonstrate from multiple angles that IL27RA acts as a central nexus linking immunotherapy resistance in breast cancer to defects in antigen presentation. On the one hand, *IL27RA* overexpression does not affect TNBC cell proliferation, migration, or PD‐L1 expression in vitro, yet it activates the PI3K/AKT signaling pathway, leading to downregulation of MHC‐I and multiple genes involved in antigen processing and presentation. In many malignancies, high MHC‐I expression is associated with enhanced antigen‐presenting capacity and reduced immune evasion [40, 41]. *IL27RA* overexpression induces AKT phosphorylation at Ser473 and Thr308, suppressing MHC‐I expression. The AKT inhibitor MK‐2206 reverses these changes, restoring tumor‐cell susceptibility to OT‐I antigen‐specific CD8^+^ T cell killing. Furthermore, TNBC cells lacking *IL27RA* exhibit pronounced tumor inhibition and reduced lung metastasis only in immunocompetent mice, with no significant differences observed in T cell‐deficient nude mice; neutralization of CD8⁺ T cells completely abolishes the survival advantage conferred by *Il27ra* deletion. Spatial transcriptomics and multiplex immunostaining further reveal that *IL27RA* is highly expressed in epithelial cells of resistant lesions and establishes a spatial exclusion pattern against CD8⁺ T cells, resulting in near‐complete loss of T cell infiltration. Together, these findings demonstrate that the IL27RA–PI3K/AKT–MHC‐I axis represents a critical pathway driving immune evasion in TNBC and promotes the development of an “immune‐excluded” tumor phenotype.

Beyond tumor cells, IL27RA expression in immune populations also profoundly shapes the composition and function of the TNBC tumor microenvironment. Using single‐cell transcriptomic profiling of tumor‐infiltrating CD45⁺ cells from *Il27ra* knockout mice, we found that *Il27ra* deficiency markedly enhanced ligand–receptor interactions among immune cells, particularly along chemokine axes connecting macrophage/monocyte populations with T and NK cells. Systemic loss of *Il27ra* increased the proportion of CD8⁺ T cells within the TME, accompanied by widespread upregulation of CCR2, whereas CXCR6 expression decreased in CD4⁺ naïve T cells, which also declined in frequency. These observations align with previous findings showing: (i) IL‐27 receptor signaling via STAT1/3 can restrict CCR5 expression, thereby suppressing CCR5‐dependent chemotactic responses and limiting the migration of CD4⁺ T cells into inflammatory sites [42, 43]; and (ii) CXCR6 is widely recognized as a key chemokine receptor defining tissue‐resident memory T cells, closely associated with tissue retention and long‐term immune memory formation [44, 45, 46, 47]. Building on this knowledge, our single‐cell data suggest that *IL27RA* deficiency may remodel CCR2‐ and CXCR6‐associated chemotactic axes to drive redistribution and functional reprogramming of T cell subsets in TNBC, indicating a bidirectional regulatory role of IL27RA in shaping T cell migration patterns and lineage differentiation trajectories. Further analysis revealed that the KO group exhibited increased proportions and functional scores of Th1‐like CD4⁺ T cells, with enrichment of multiple inflammatory and immune‐related pathways. In contrast, CD4 Tcm cells showed reduced proportions and decreased functional scores, while certain Treg subsets displayed a modest numerical increase but signs of functional instability. Collectively, *IL27RA* deletion enhances CD8⁺ T cell inflammatory chemotaxis and Th1‐like responses, while alleviating metabolic and apoptotic stress in effector populations—thereby supporting the establishment of a more durable antitumor T cell response. These immune‐cell findings support the enhanced PD‐1 blockade sensitivity observed with IL27RA inhibition.

At the level of NK cells, our single‐cell analysis delineated how *IL27RA* deficiency reshapes NK‐cell subset composition and functional states. Although overall NK‐cell infiltration was comparable between KO and WT mice, loss of *Il27ra* markedly promoted the differentiation of NK cells toward more mature and cytotoxic lineages. We classified the major NK subsets into NK_Inpp4b and NK_Hspa1b: the former was enriched in the KO group and characterized by elevated expression of cytotoxic and chemotactic molecules such as *Gzmb*, *Prf1*, and *Ccr5*, along with higher levels of tissue‐residency markers including *Cd69*, *Itga1*, and *Rgs1*. In contrast, NK_Hspa1b dominated in WT tumors and expressed higher levels of Hspa1a/b and migration‐associated factors such as *Klf2* and *Sell*. Pseudotime analysis indicated that NK cells progress along a trajectory from NK_Hspa1b toward NK_Inpp4b and other mature cytotoxic states, with cytotoxic molecules gradually increasing and inhibitory receptors decreasing. Notably, NK cells in the KO group were more enriched at the terminal mature stages, and functional assays confirmed enhanced degranulation and cytolytic activity of NK cells infiltrating KO tumors. In light of prior reports showing that *DOCK8* deficiency leads to NK‐cell dysfunction [48, 49], our findings suggest that *IL27RA* deletion may alleviate NK‐cell stress and functional constraints, thereby driving differentiation toward tissue‐resident, highly cytotoxic NK subsets. These NK populations likely act in concert with CD8⁺ T cells to mediate more effective immune control of TNBC.

Our study identifies IL27RA as both a key driver of immune evasion in TNBC and a promising biomarker and therapeutic target. On the one hand, patients with high IL27RA expression and low MHC‐I levels may represent a high‐risk subgroup that could particularly benefit from combined therapies involving IL27RA inhibition and PD‐1/PD‐L1 blockade. On the other hand, combining IL27RA blockade with PI3K/AKT inhibitors (e.g., Alpelisib) or immune‐potentiating agonists (e.g., CD40 antibodies) may synergize to enhance antitumor activity in TNBC. Moreover, our findings from *Il27ra* knockout mice suggest that systemic dampening of IL‐27 signaling may itself possess antitumor potential. However, this strategy requires careful consideration of the physiological role of IL‐27 in maintaining Th17/Treg balance and antimicrobial immunity [50]. Future efforts should therefore focus on developing precision‐targeted delivery approaches that restrict *IL27RA* inhibition to tumor cells or specific immune subsets—such as antibody–drug conjugates or conditional gene‐editing strategies—to maximize therapeutic efficacy while minimizing systemic immune perturbation.

This study has several limitations. First, our clinical and single‐cell cohorts primarily focused on TNBC, with a relatively small sample size. The single‐cell dataset included only three TNBC patients who failed to achieve pCR after neoadjuvant therapy and an additional three CR patients for validation. Given the substantial molecular and immunological heterogeneity within TNBC, the expression pattern of IL27RA and its impact on the TME may vary across different TNBC subtypes. Second, our current data are largely derived from TNBC, and we have not yet systematically compared IL27RA expression or its prognostic relevance across other breast cancer subtypes, such as ER⁺ and HER2⁺ disease, which limits the generalizability of our conclusions to the entire breast cancer spectrum. Third, in our in vivo models, we primarily used *Il27ra* knockout mice and *IL27RA* knockout/overexpression tumor cells, thereby demonstrating the role of IL27RA mainly from a loss‐of‐function perspective. A complementary gain‐of‐function model with *IL27RA* overexpression in vivo is still needed to provide reciprocal validation. Finally, although we revealed extensive remodeling of T‐ and NK‐cell subset composition and migratory programs upon *IL27RA* deficiency, the precise causal contributions of individual immune subpopulations to antitumor immunity remain to be determined and will require further investigation using patient‐derived xenografts (PDX) and larger clinical cohorts.

In summary, this study integrates single‐cell and spatial analyses to uncover the pivotal role of IL27RA in immunotherapy resistance in TNBC. We identify the IL27RA–PI3K/AKT–MHC‐I axis as a key pathway linking tumor‐cell immune evasion to downstream dysfunction of specific immune populations and delineate how IL27RA loss reshapes T cell expansion and chemotactic networks, NK‐cell cytotoxic maturation, and overall TME architecture. Our findings establish a mechanistic framework for PD‐1 blockade resistance and highlight IL27RA as a promising biomarker for stratification and a therapeutic target, advancing precision immunotherapy for TNBC.

## 5 Conclusion

Collectively, our single‐cell–resolved analyses uncover a central mechanism of immunotherapy resistance in TNBC, establishing IL27RA as a key orchestrator of tumor immune evasion through a non‐canonical PI3K/AKT–MHC‐I regulatory axis. By suppressing antigen presentation and functionally impairing the infiltration and cytotoxic capacity of CD8⁺ T lymphocytes, IL27RA fosters the development of an immune‐excluded, immunosuppressive niche. Therapeutic disruption of IL27RA reverses these effects, reshaping the TME, restoring antitumor immunity, and restraining malignant progression. These findings highlight IL27RA as a promising biomarker and actionable therapeutic target for improving the efficacy of PD‐1–based immunotherapy in TNBC.

## Author Contributions

Jiachi Xu completed the major experiments and manuscript writing; Danhua Zhang, Hui Zhou, and Wenjun Yi designed this study and guided the key experimental concepts; Qian Long, Meirong Zhou, Qitong Chen, Qingchun Liang and Jing Peng were responsible for the collection of clinical cases. All authors read and approved the final manuscript.

## Ethics Approval And Consent To Participate

Human specimen collection and animal experiments were reviewed and approved by the Ethics Committee of Xiangya Second Hospital, Central South University. The detailed ethical approval numbers are outlined in sections 2.1 and 2.2.

## Conflicts of Interest

The authors declare no conflicts of interest.

## Supporting information




**Supporting File**: advs73602‐sup‐0001‐SuppMat.docx.

## Data Availability

The data that support the findings of this study are available from the corresponding author upon reasonable request.

## References

[1] F. Bray , M. Laversanne , H. Sung , et al., “Global Cancer Statistics 2022: GLOBOCAN Estimates of Incidence and Mortality Worldwide for 36 Cancers in 185 Countries,” CA: A Cancer Journal for Clinicians 74 (2024): 229–263. 38572751 10.3322/caac.21834

[2] D. S. Chen and I. Mellman , “Oncology Meets Immunology: The Cancer‐immunity Cycle,” Immunity 39 (2013): 1–10. 23890059 10.1016/j.immuni.2013.07.012

[3] P. Sharma , S. Goswami , D. Raychaudhuri , et al., “Immune Checkpoint Therapy—Current Perspectives and Future Directions,” Cell 186 (2023): 1652–1669. 37059068 10.1016/j.cell.2023.03.006

[4] K. Nutsch , K. L. Banta , T. D. Wu , et al., “TIGIT and PD‐L1 co‐blockade Promotes Clonal Expansion of Multipotent, Non‐Exhausted Antitumor T Cells by Facilitating co‐stimulation,” Nature Cancer 5 (2024): 1834–1851. 39681653 10.1038/s43018-024-00870-6 PMC11663793

[5] K. O. Dixon , M. Tabaka , M. A. Schramm , et al., “TIM‐3 Restrains Anti‐Tumour Immunity by Regulating Inflammasome Activation,” Nature 595 (2021): 101–106. 34108686 10.1038/s41586-021-03626-9 PMC8627694

[6] Y. Jiang , A. Dai , Y. Huang , et al., “Ligand‐induced Ubiquitination Unleashes LAG3 Immune Checkpoint Function by Hindering Membrane Sequestration of Signaling Motifs,” Cell 188 (2025): 2354–2371.e18.e2318. 40101708 10.1016/j.cell.2025.02.014

[7] A. Ribas and J. D. Wolchok , Cancer Immunotherapy Using Checkpoint Blockade 359 (2018): 1350–1355. 10.1126/science.aar4060 PMC7391259 29567705

[8] Y. Zhang , H. Chen , H. Mo , et al., “Single‐Cell Analyses Reveal Key Immune Cell Subsets Associated With Response to PD‐L1 Blockade in Triple‐negative Breast Cancer,” Cancer Cell 39 (2021): 1578–1593.e1578. 34653365 10.1016/j.ccell.2021.09.010

[9] D. S. Chen and I. Mellman , “Elements of Cancer Immunity and the Cancer–Immune Set Point,” Nature 541 (2017): 321–330. 28102259 10.1038/nature21349

[10] P. S. Hegde and D. S. Chen , “Top 10 Challenges in Cancer Immunotherapy,” Immunity 52 (2020): 17–35. 31940268 10.1016/j.immuni.2019.12.011

[11] P. Bonaventura , T. Shekarian , V. Alcazer , et al., “Cold Tumors: A Therapeutic Challenge for Immunotherapy,” Frontiers in Immunology 10 (2019): 168. 30800125 10.3389/fimmu.2019.00168 PMC6376112

[12] H. E. Vidana Gamage , S. T. Albright , A. J. Smith , et al., “Development of NR0B2 as a Therapeutic Target for the Re‐Education of Tumor Associated Myeloid Cells,” Cancer Letters 597 (2024): 217086. 38944231 10.1016/j.canlet.2024.217086 PMC11890212

[13] Y. Liu , Y. Hu , J. Xue , et al., “Advances in Immunotherapy for Triple‐Negative Breast Cancer,” Molecular Cancer 22 (2023): 145. 37660039 10.1186/s12943-023-01850-7 PMC10474743

[14] P. Schmid , J. Cortes , L. Pusztai , et al., “Pembrolizumab for Early Triple‐Negative Breast Cancer,” New England Journal of Medicine 382 (2020): 810–821. 32101663 10.1056/NEJMoa1910549

[15] L. Chen , H. Li , H. Zhang , et al., “Camrelizumab vs Placebo in Combination With Chemotherapy as Neoadjuvant Treatment in Patients With Early or Locally Advanced Triple‐Negative Breast Cancer,” Jama 333 (2025): 673–681. 39671272 10.1001/jama.2024.23560 PMC11862970

[16] N. Furukawa , V. Stearns , C. A. Santa‐Maria , and A. S. Popel , “The Tumor Microenvironment and Triple‐Negative Breast Cancer Aggressiveness: Shedding Light on Mechanisms and Targeting,” Expert Opinion on Therapeutic Targets 26 (2022): 1041–1056. 36657483 10.1080/14728222.2022.2170779 PMC10189896

[17] D. Hanahan and R. A. Weinberg , “Hallmarks of Cancer: The next Generation,” Cell 144 (2011): 646–674. 21376230 10.1016/j.cell.2011.02.013

[18] H. Sabit , A. Adel , M. M. Abdelfattah , et al., “The Role of Tumor Microenvironment and Immune Cell Crosstalk in Triple‐negative Breast Cancer (TNBC): Emerging Therapeutic Opportunities,” Cancer Letters 628 (2025): 217865. 40516902 10.1016/j.canlet.2025.217865

[19] E. J. Wherry and M. Kurachi , “Molecular and Cellular Insights Into T Cell Exhaustion,” Nature Reviews Immunology 15 (2015): 486–499. 10.1038/nri3862 PMC4889009 26205583

[20] M. Binnewies , E. W. Roberts , K. Kersten , et al., “Understanding the Tumor Immune Microenvironment (TIME) for Effective Therapy,” Nature Medicine 24 (2018): 541–550. 10.1038/s41591-018-0014-x PMC5998822 29686425

[21] C. Trapnell , “Defining Cell Types and States With Single‐Cell Genomics,” Genome Research 25 (2015): 1491–1498. 26430159 10.1101/gr.190595.115 PMC4579334

[22] F. Jia , S. Sun , J. Li , et al., “Neoadjuvant Chemotherapy‐induced Remodeling of Human Hormonal Receptor‐Positive Breast Cancer Revealed by Single‐Cell RNA Sequencing,” Cancer Letters 585 (2024): 216656. 38266804 10.1016/j.canlet.2024.216656

[23] X. Liu , A. Zhao , S. Xiao , et al., “PD ‐1: A Critical Player and Target for Immune Normalization,” Immunology 172 (2024): 181–197. 38269617 10.1111/imm.13755

[24] N. Percie du Sert , V. Hurst , A. Ahluwalia , et al., “The ARRIVE Guidelines 2.0: Updated Guidelines for Reporting Animal Research,” British Journal of Pharmacology 177 (2020): 3617–3624. 32662519 10.1111/bph.15193 PMC7393194

[25] M. Ashburner , C. A. Ball , J. A. Blake , et al., “Gene Ontology: Tool for the Unification of Biology The Gene Ontology Consortium,” Nature Genetics 25 (2000): 25–29. 10802651 10.1038/75556 PMC3037419

[26] M. Kanehisa and S. Goto , “KEGG: Kyoto encyclopedia of Genes and Genomes,” Nucleic Acids Research 28 (2000): 27–30. 10592173 10.1093/nar/28.1.27 PMC102409

[27] L. Garcia‐Alonso , C. H. Holland , M. M. Ibrahim , D. Turei , and J. Saez‐Rodriguez , “Benchmark and Integration of Resources for the Estimation of Human Transcription Factor Activities,” Genome Research 29 (2019): 1363–1375. 31340985 10.1101/gr.240663.118 PMC6673718

[28] B. Győrffy , “Survival Analysis Across the Entire Transcriptome Identifies Biomarkers With the Highest Prognostic Power in Breast Cancer,” Computational and Structural Biotechnology Journal 19 (2021): 4101–4109. 34527184 10.1016/j.csbj.2021.07.014 PMC8339292

[29] J. Xu , H. Gao , M. S. Azhar , et al., “Interleukin Signaling in the Regulation of Natural Killer Cells Biology in Breast Cancer,” Frontiers in Immunology 15 (2024): 1449441. 39380989 10.3389/fimmu.2024.1449441 PMC11459090

[30] D. Dibra , J. J. Cutrera , X. Xia , M. P. Birkenbach , and S. Li , “Expression of WSX1 in Tumors Sensitizes IL‐27 Signaling‐Independent Natural Killer Cell Surveillance,” Cancer Research 69 (2009): 5505–5513. 19549909 10.1158/0008-5472.CAN-08-4311 PMC2706921

[31] D. L. Greiner , R. A. Hesselton , and L. D. Shultz , “SCID Mouse Models of Human Stem Cell Engraftment,” Stem Cells 16 (1998): 166–177. 9617892 10.1002/stem.160166

[32] A. E. Yuzhalin , F. J. Lowery , Y. Saito , et al., “Astrocyte‐induced Cdk5 Expedites Breast Cancer Brain Metastasis by Suppressing MHC‐I Expression to Evade Immune Recognition,” Nature Cell Biology 26 (2024): 1773–1789. 39304713 10.1038/s41556-024-01509-5 PMC11676029

[33] V. Peng , T. Trsan , R. Sudan , et al., “Inositol Phosphatase INPP4B Sustains ILC1s and Intratumoral NK Cells Through an AKT‐Driven Pathway,” Journal of Experimental Medicine 221 (2024): 20230124. 10.1084/jem.20230124 PMC10783437 38197946

[34] H. N. Bell and W. Zou , “Beyond the Barrier: Unraveling the Mechanisms of Immunotherapy Resistance,” Annual Review of Immunology 42 (2024): 521–550. 10.1146/annurev-immunol-101819-024752 PMC11213679 38382538

[35] Z. Yang , X. Liu , J. Zhu , et al., “Inhibiting Intracellular CD28 in Cancer Cells Enhances Antitumor Immunity and Overcomes Anti‐PD‐1 Resistance via Targeting PD‐L1,” Cancer Cell 43 (2025): 86–102.e86. 39672166 10.1016/j.ccell.2024.11.008

[36] L. Xu , K. Saunders , S. P. Huang , et al., “A Comprehensive Single‐cell Breast Tumor Atlas Defines Epithelial and Immune Heterogeneity and Interactions Predicting Anti‐PD‐1 Therapy Response,” Cell Reports Medicine 5 (2024): 101511. 38614094 10.1016/j.xcrm.2024.101511 PMC11148512

[37] J. I. Griffiths , P. A. Cosgrove , E. F. Medina , et al., “Cellular Interactions Within the Immune Microenvironment Underpins Resistance to Cell Cycle Inhibition in Breast Cancers,” Nature Communications 16 (2025): 2132. 10.1038/s41467-025-56279-x PMC11876604 40032842

[38] K. M. Sullivan , X. Jiang , P. Guha , et al., “Blockade of Interleukin 10 Potentiates Antitumour Immune Function in Human Colorectal Cancer Liver Metastases,” Gut 72 (2023): 325–337. 35705369 10.1136/gutjnl-2021-325808 PMC9872249

[39] Y. Chen , M. Anwar , X. Wang , B. Zhang , and B. Ma , “Integrative Transcriptomic and Single‐Cell Analysis Reveals IL27RA as a Key Immune Regulator and Therapeutic Indicator in Breast Cancer,” Discover Oncology 16 (2025): 977. 40450602 10.1007/s12672-025-02811-w PMC12127260

[40] K. Kawase , S. Kawashima , J. Nagasaki , et al., “High Expression of MHC Class I Overcomes Cancer Immunotherapy Resistance Due to IFNγ Signaling Pathway Defects,” Cancer Immunology Research 11 (2023): 895–908. 37062030 10.1158/2326-6066.CIR-22-0815

[41] X. Chen , Q. Lu , H. Zhou , et al., “A Membrane‐associated MHC‐I Inhibitory Axis for Cancer Immune Evasion,” Cell 186 (2023): 3903–3920.e3903. 37557169 10.1016/j.cell.2023.07.016 PMC10961051

[42] A. Hu , J. Zhu , C. Zeng , et al., “IL‐27 Induces CCL5 Production By T Lymphocytes, Which Contributes to Antitumor Activity,” Journal of Immunology 208 (2022): 2239–2245. 10.4049/jimmunol.2100885 PMC9050872 35418466

[43] Y. Mei , Z. Lv , L. Xiong , H. Zhang , N. Yin , and H. Qi , “The Dual Role of IL‐27 in CD4+T Cells,” Molecular Immunology 138 (2021): 172–180. 34438225 10.1016/j.molimm.2021.08.001

[44] N. Mabrouk , T. Tran , I. Sam , et al., “CXCR6 Expressing T Cells: Functions and Role in the Control of Tumors,” Frontiers in Immunology 13 (2022): 1022136. 36311728 10.3389/fimmu.2022.1022136 PMC9597613

[45] R. Muthuswamy , A. R. McGray , S. Battaglia , et al., “CXCR6 by Increasing Retention of Memory CD8 + T Cells in the Ovarian Tumor Microenvironment Promotes Immunosurveillance and Control of Ovarian Cancer,” Journal for ImmunoTherapy of Cancer 9 (2021): 003329. 10.1136/jitc-2021-003329 PMC8491420 34607898

[46] A. N. Wein , S. R. McMaster , S. Takamura , et al., “CXCR6 regulates Localization of Tissue‐resident Memory CD8 T Cells to the Airways,” Journal of Experimental Medicine 216 (2019): 2748–2762. 31558615 10.1084/jem.20181308 PMC6888981

[47] T. A. Heim , O. Ibrahim , Z. Lin , et al., “CXCR6 Promotes Dermal CD8+ T Cell Survival and Transition to Long‐Term Tissue Residence,” The Journal of Immunology (2025). 10.1093/jimmun/vkaf219 PMC12412903 40906897

[48] F. J. Alroqi , L. M. Charbonnier , S. Keles , et al., “DOCK8 Deficiency Presenting as an IPEX‐Like Disorder,” Journal of Clinical Immunology 37 (2017): 811–819. 29058101 10.1007/s10875-017-0451-1 PMC5691358

[49] M. C. Mizesko , P. P. Banerjee , L. Monaco‐Shawver , et al., “Defective Actin Accumulation Impairs human Natural Killer Cell Function in Patients With Dedicator of Cytokinesis 8 Deficiency,” Journal of Allergy and Clinical Immunology 131 (2013): 840–848. 23380217 10.1016/j.jaci.2012.12.1568 PMC3646579

[50] H. Yoshida and C. A. Hunter , “The Immunobiology of Interleukin‐27,” Annual Review of Immunology 33 (2015): 417–443. 10.1146/annurev-immunol-032414-112134 25861977

