# Supplementary material for: Targeting IL27RA Enhances Immunotherapy in Triple‐Negative Breast Cancer by Modulating Tumor Cells and the Tumor Microenvironment
Source: Adv Sci (Weinh). 2026 Jan 4;13(14):e16703. doi: 10.1002/advs.202516703 (PMC12970260; doi:10.1002/advs.202516703)
Supplement: Supplementary file 1 — Supporting File: advs73602‐sup‐0001‐SuppMat.docx. [file ADVS-13-e16703-s001.docx]

**
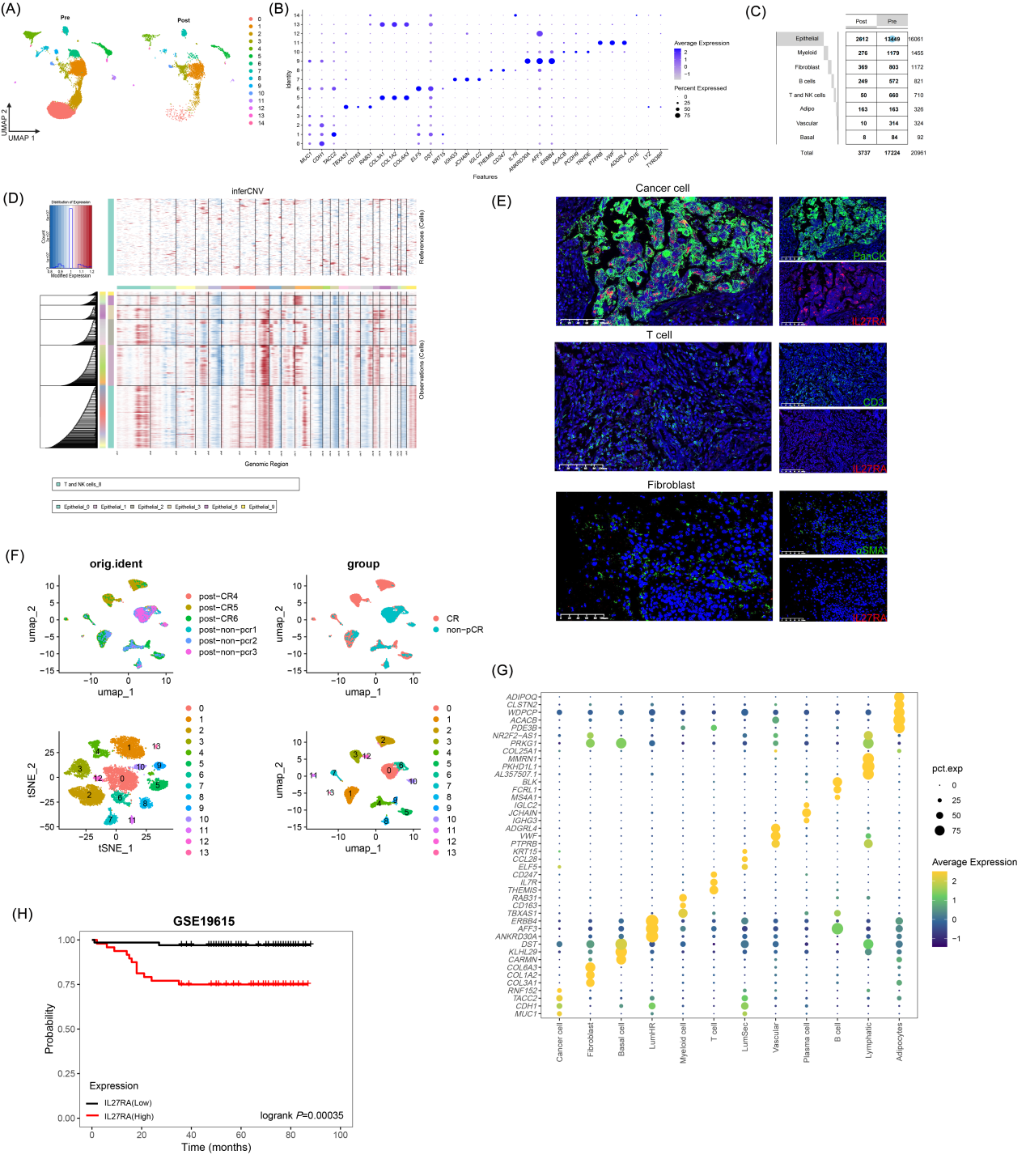
Figure S1. Multidimensional validation of IL27RA as a marker of immunotherapy-resistant epithelial cells in TNBC.**

# (A) UMAP plots showing differences in cell-cluster distributions in pre- and post-treatment samples from TNBC patients who failed to achieve pCR after neoadjuvant therapy. (B) Bubble plot displaying normalized expression of canonical marker genes for each annotated cell cluster. (C) Balloon plot showing cell counts of major cell types in pre- versus post-treatment specimens from the same TNBC patients. (D) InferCNV analysis identifying malignant epithelial subclusters based on copy-number variation patterns.

# (E) Representative immunofluorescence staining shows IL27RA expression in tumor cells, T cells, and fibroblasts. PanCK (green), CD3 (green), and α-SMA (green) were used to identify cancer cells, T lymphocytes, and fibroblasts, respectively. (F) UMAP plots showing the distribution of single cells from TNBC patients with different clinical outcomes after neoadjuvant therapy: distribution by sample (top left), by clinical subgroup (top right), and distribution of annotated clusters visualized by t-SNE (bottom left) and UMAP (bottom right). (G) Bubble plot showing normalized expression of marker genes corresponding to the cell clusters described in panel (E). (H) Kaplan-Meier curves of distant metastasis-free survival (DMFS) in breast cancer patients stratified by *IL27RA* expression (low vs. high) based on the GEO dataset.

# Statistical significance was assessed using the log-rank test for survival analyses in (G). Ns, not significant; **p* < 0.05; ***p* < 0.01; ****p* < 0.001; *****p* < 0.0001.


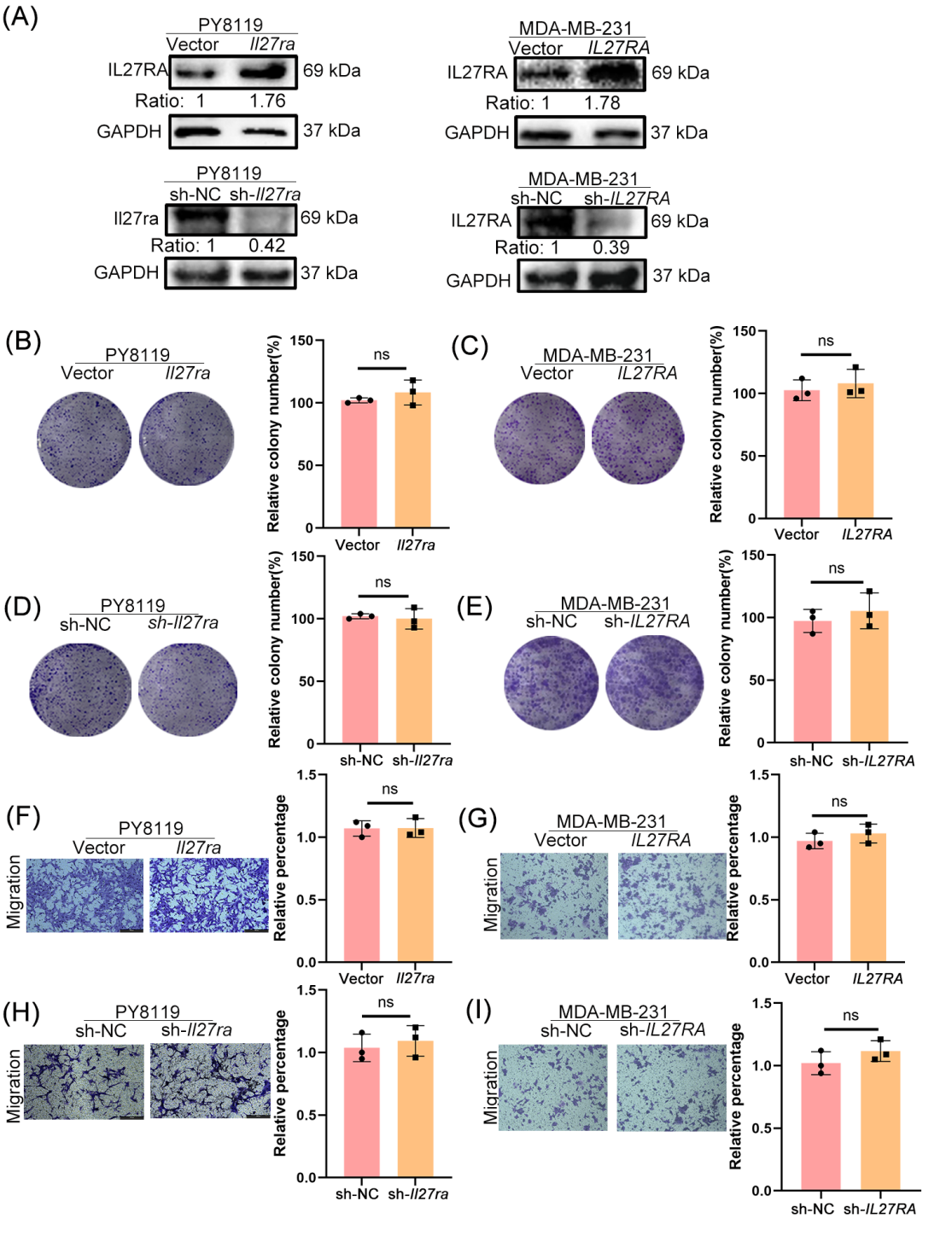


# Figure S2. IL27RA does not independently affect TNBC cell proliferation or migration.

# (A) Western blot validation of IL27RA overexpression (top) and knockdown (bottom) in stable PY8119 (left) and MDA-MB-231 (right) cell lines. (B-D) Colony formation assays assessing the proliferative capacity of *IL27RA*-overexpressing or IL27RA-knockdown TNBC cell lines. (F-I) Transwell migration assays evaluating the migratory ability of the corresponding stable cell lines.

# Data represent three independent biological replicates (n = 3) and are presented as mean±SD. Statistical significance was assessed using two-tailed unpaired Student’s t-tests; ns, not significant.


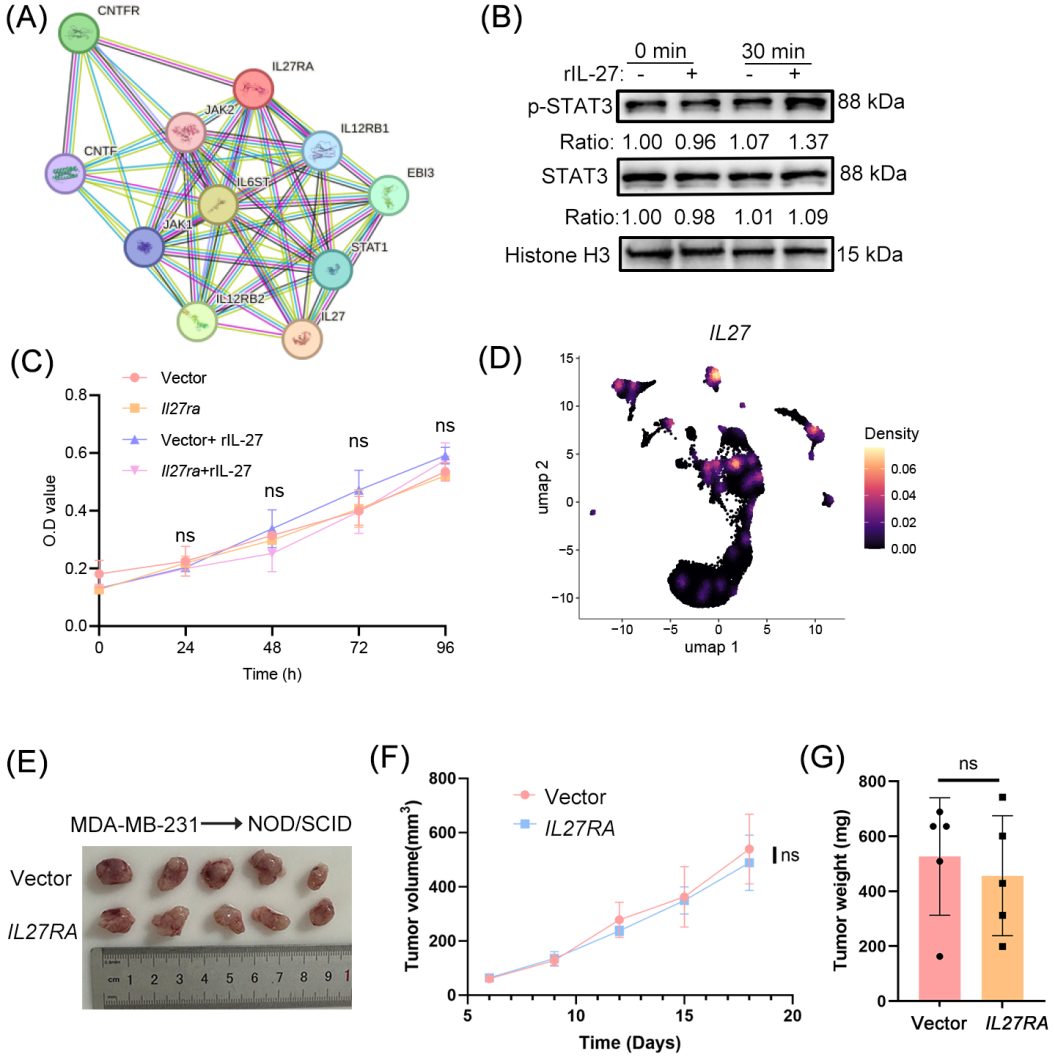


**Figure S3. IL27RA-mediated regulation of TNBC growth is not solely dependent on IL27RA itself or activation of the IL-27 signaling axis.**

# STRING-generated protein-protein interaction network of IL27RA. (B) Western blot analysis of STAT3 phosphorylation at indicated time points following stimulation with recombinant IL-27 (rIL-27). (C) Growth curves of PY8119-*Il27ra* cells treated with rIL-27 for the indicated durations.

# UMAP plot displaying *IL27* expression density across all cell types. (E-G) Quantitative analysis of orthotopic tumor formation in NOD/SCID mice injected with MDA-MB-231 cells overexpressing *IL27RA* (n = 5). Representative tumor images at collection time points (E), longitudinal tumor volume measurements (F), and endpoint tumor weights (G) are shown.

# Data are presented as mean±SD. Statistical significance was assessed by two-way ANOVA with Sidak/Tukey multiple-comparisons tests for growth and tumor-volume analyses (C and F), and by two-tailed unpaired Student’s t-test for endpoint tumor weights (G). Ns, not significant.

**
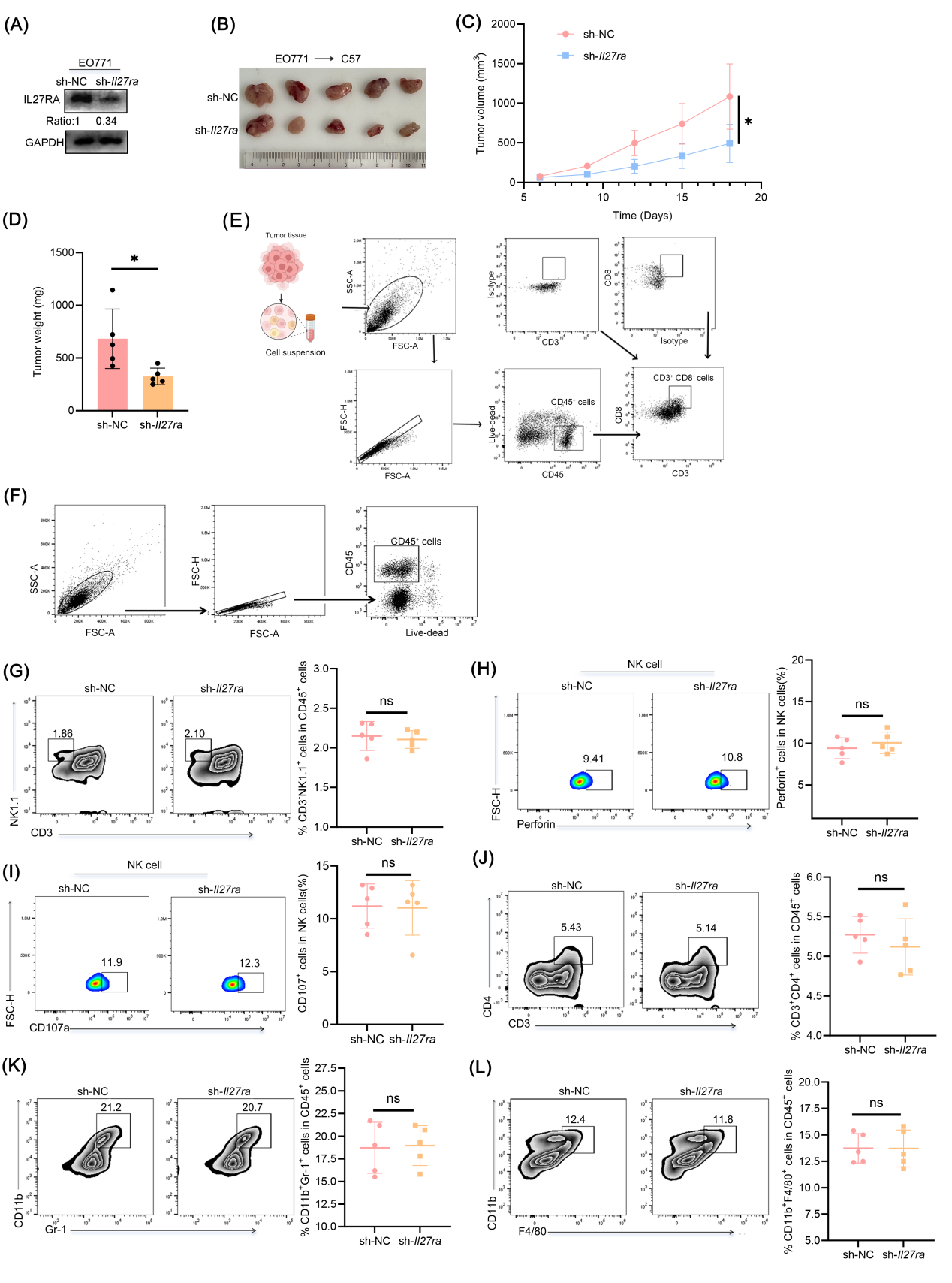
Figure S4. IL27RA knockout-mediated tumor suppression in breast cancer is dependent on CD8⁺ T cells.**

# (A) Western blot validation of the EO771.sh-*Il27ra* stable knockdown cell line. (B-D) Quantitative analysis of orthotopic tumor formation in C57BL/6J mice injected with EO771-sh-*Il27ra* or control cells (n = 5). Representative tumor images at indicated time points (B), longitudinal tumor growth curves (C), and endpoint tumor weights (D) are shown. (E) Flow-cytometry gating strategy for tumor-infiltrating CD8⁺ T cells. (F) Gating strategy for tumor-infiltrating immune cells (CD45⁺). (G-I) Comparison of NK-cell infiltration (G) and NK-cell cytotoxic function—Perforin (H) and CD107a (I)—in tumors formed by *Il27ra*-knockdown versus control cells (n=5). (J-L) Proportions of tumor-infiltrating CD4⁺ T cells (J), MDSCs (K), and macrophages (L) following implantation of *Il27ra*-knockdown versus control EO771 cells (n=5).

# Data are presented as mean ± SD. Statistical significance was determined using two-way ANOVA with Sidak’s multiple-comparisons test for tumor growth curves (C), and two-tailed unpaired Student’s t-tests for endpoint tumor weight (D), NK-cell infiltration and function (G-I), and proportions of CD4⁺ T cells, MDSCs, and macrophages (J-L). Ns, not significant; **p* < 0.05. The schematic diagram was created using https://BioRender.com.


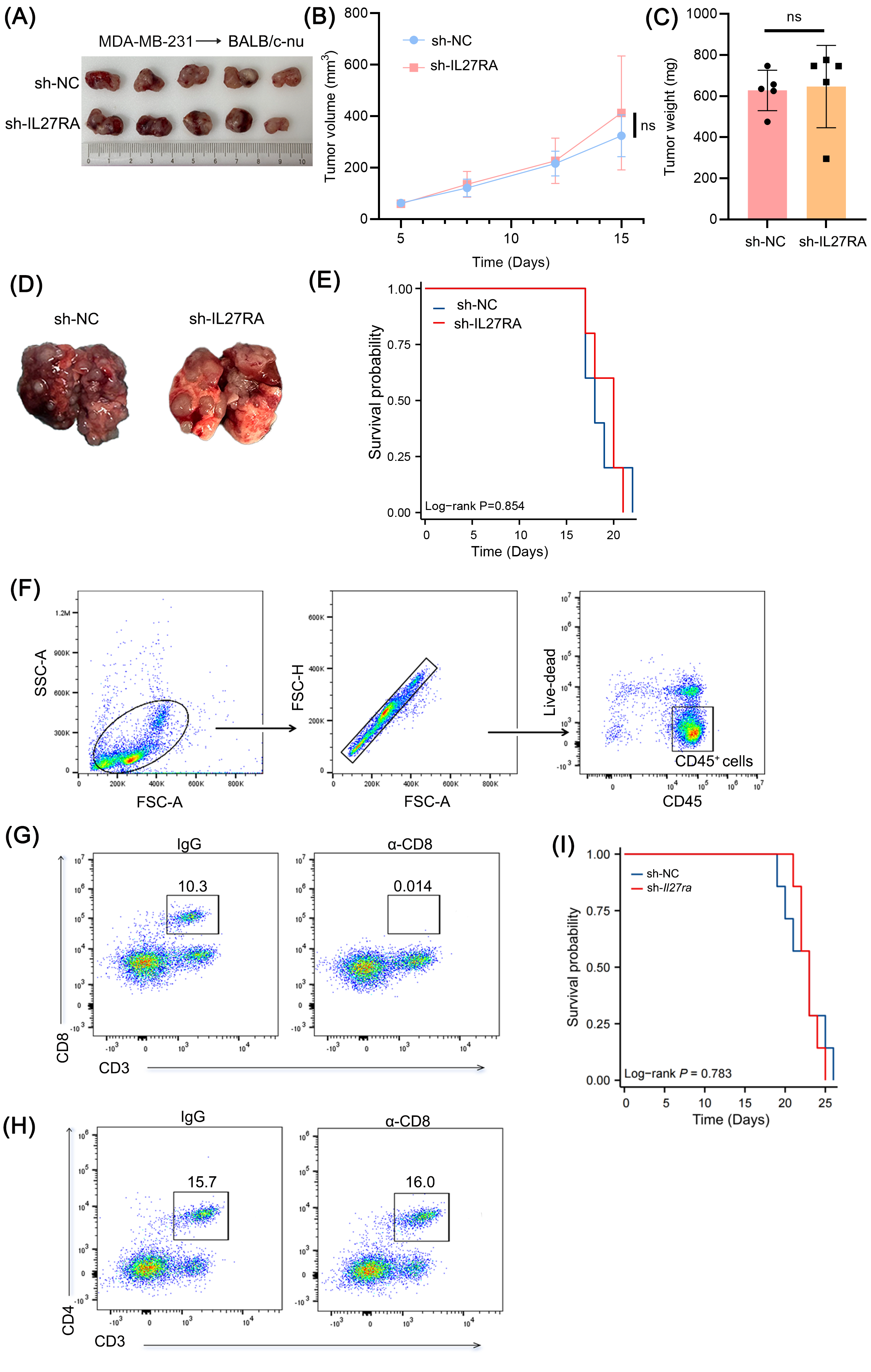


# Figure S5. CD8⁺ T cell depletion abolishes the survival advantage conferred by *IL27RA* knockout.

# (A-C) Quantitative analysis of orthotopic tumor formation in BALB/c-nu mice injected with MDA-MB-231.sh-*IL27RA* or control cells (n = 5). Representative tumor images (A), longitudinal tumor growth curves (B), and endpoint tumor weights (C) are shown. (D-E) Experimental lung metastasis model established via tail-vein injection of the same cell lines (n = 5). Representative lung images (D) and overall survival curves (E) are presented. (F) Flow-cytometry gating strategy for tumor-infiltrating lymphocytes. (G-H) Flow-cytometry analysis showing the proportions of tumor-infiltrating CD8⁺ T cells (G) and CD4⁺ T cells (H) after *in vivo* CD8⁺ T cell neutralization. (I) Survival curves of mice with lung metastases following CD8⁺ T cell depletion (n = 7).

# Data are presented as mean ± SD. Statistical significance was assessed by two-way ANOVA with Sidak’s multiple-comparisons test for tumor growth curves (B), two-tailed unpaired Student’s t-test for endpoint tumor weights (C), and log-rank tests for survival analyses (E, I). Ns, not significant.


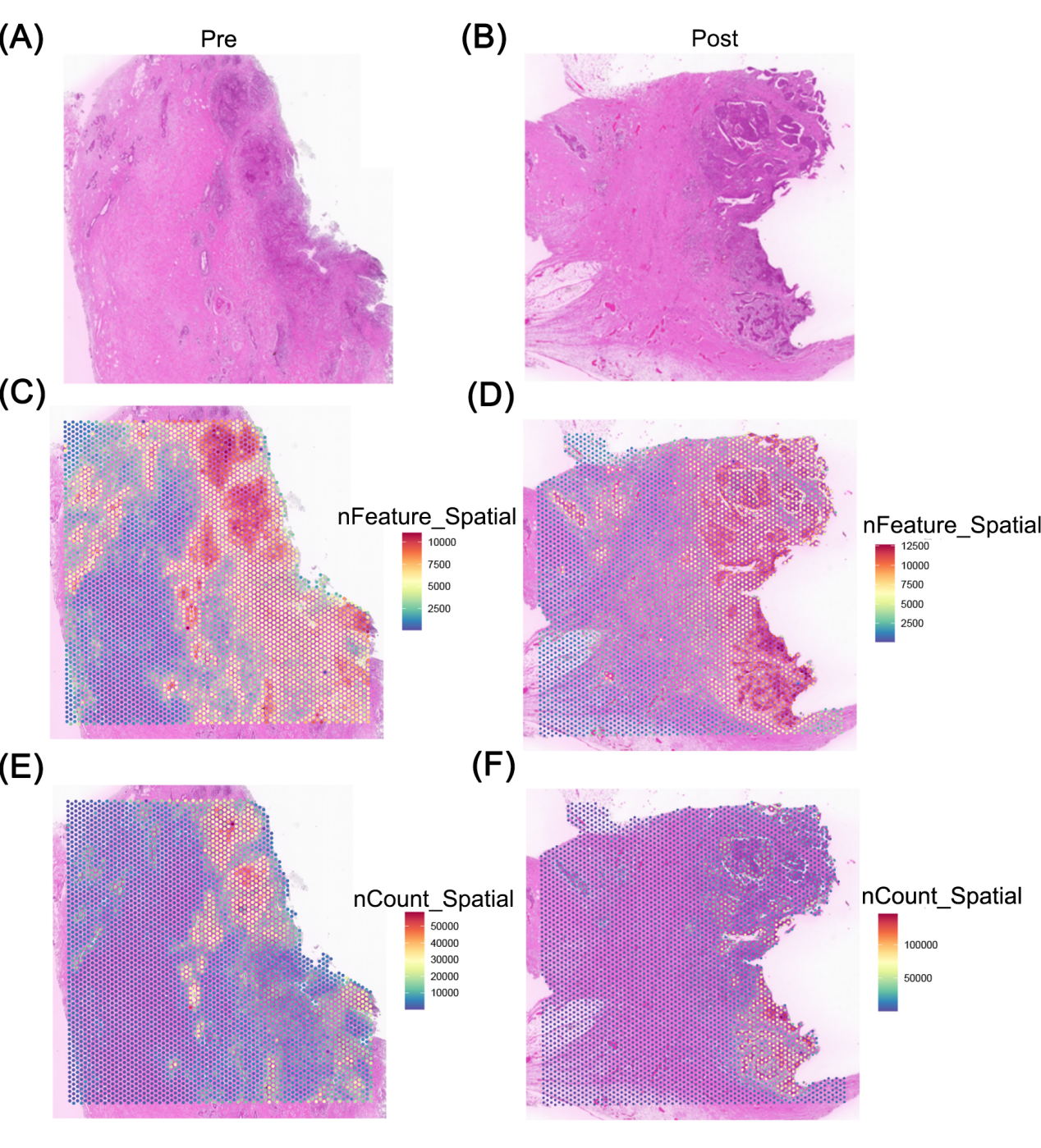
**Figure S6. Overview of spatial transcriptomic profiling of TNBC before and after neoadjuvant therapy.**

# (A and B) H&E staining of tumor tissues collected before (A) and after (B) neoadjuvant therapy. (C and D) Heatmaps showing the number of detected genes per spatial region in pre-treatment (C) and post-treatment (D) tumor sections. (E and F) Heatmaps showing the number of detected UMIs per spatial region in pre-treatment (E) and post-treatment (F) tumor sections.


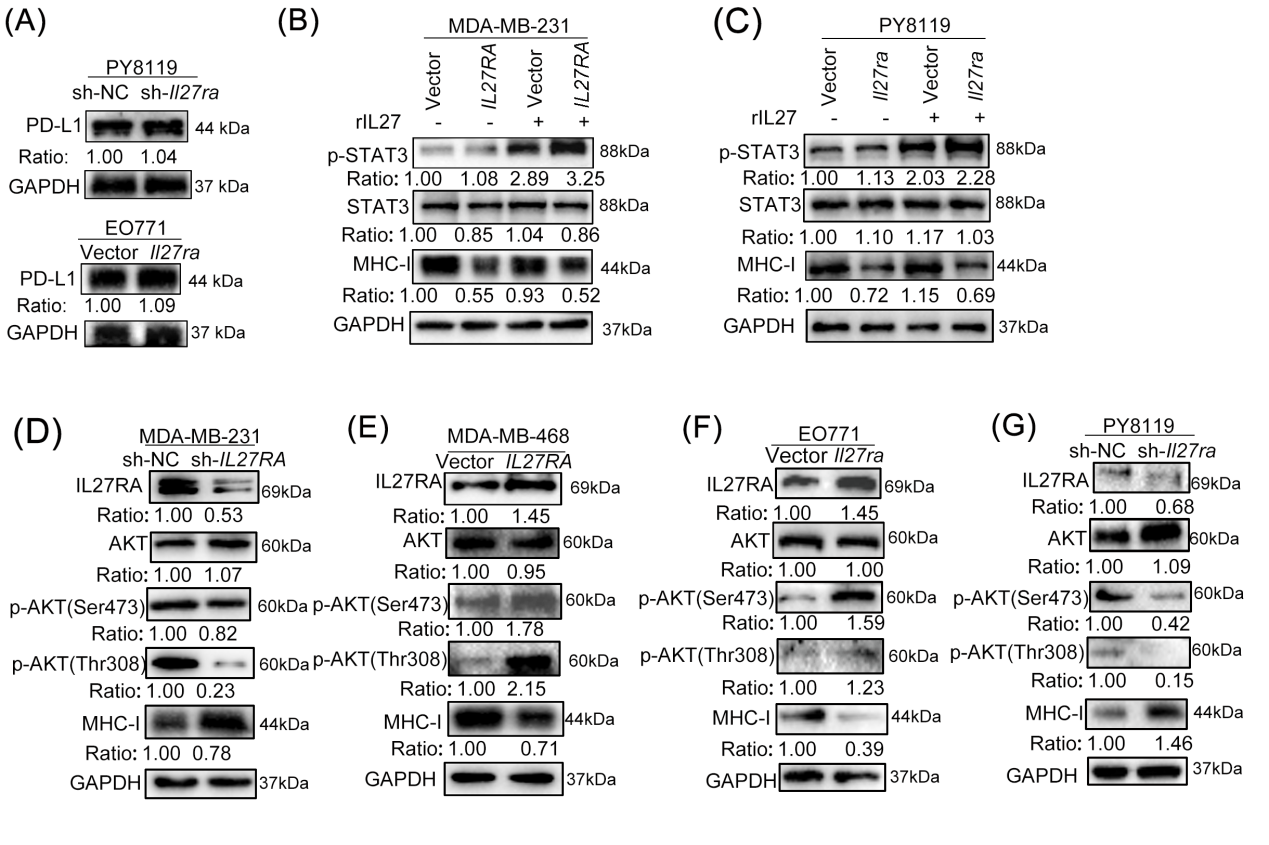


# Figure S7. Immunoblot analysis of downstream signaling molecules in breast cancer cells with stable *IL27RA* knockdown or overexpression.

1. Immunoblot analysis of PD-L1 expression in breast cancer cell lines following *Il27ra* knockdown (top) or *Il27ra* overexpression (bottom). GAPDH served as a loading control (n=3).
   (B and C) Immunoblot analysis of p-STAT3, STAT3, and MHC-I in MDA-MB-231 (B) cells overexpressing *IL27RA* and PY8119 (C) cells overexpressing *Il27ra*, treated with or without recombinant IL-27(n=3).
   (D-G) Immunoblot analysis of IL27RA, total AKT, p-AKT (Ser473), p-AKT (Thr308), and MHC-I in MDA-MB-231 (D) and MDA-MB-468 (E) cells overexpressing *IL27RA*, and EO771 (F) and PY8119 (G) cells overexpressing *Il27ra* (n=3). GAPDH served as a loading control.


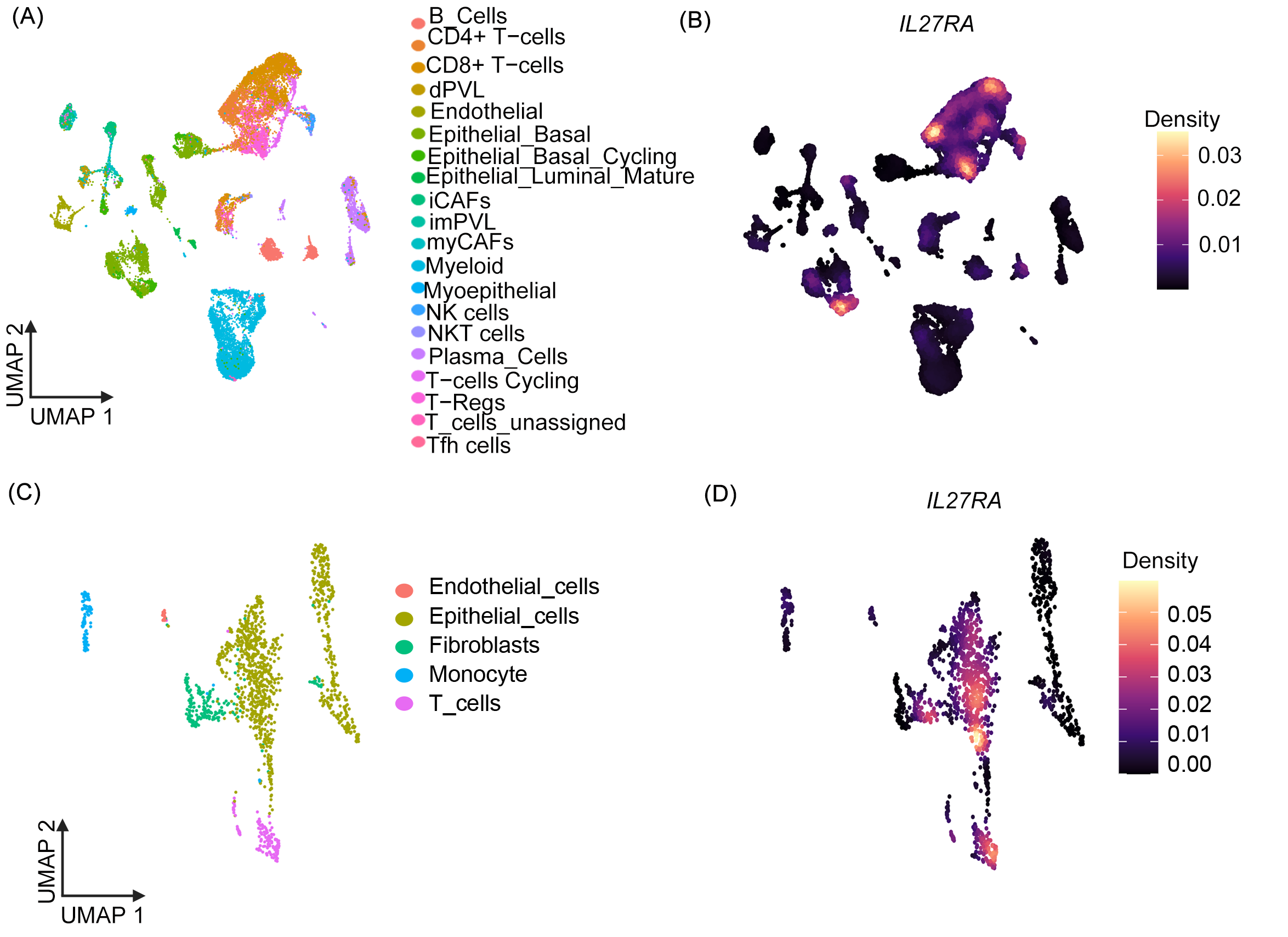


# Figure S8. Analysis of IL27RA expression in TNBC single-cell datasets from independent external databases.

# (A-B) UMAP plots showing major cell clusters (A) and *IL27RA* expression (B) in single-cell RNA-seq data from five TNBC patient samples (European Nucleotide Archive (ENA) database, PRJEB35405). (C-D) UMAP plots showing major cell clusters (C) and *IL27RA* expression (D) in single-cell RNA-seq data from six TNBC patient samples (GEO database, GSE118389).


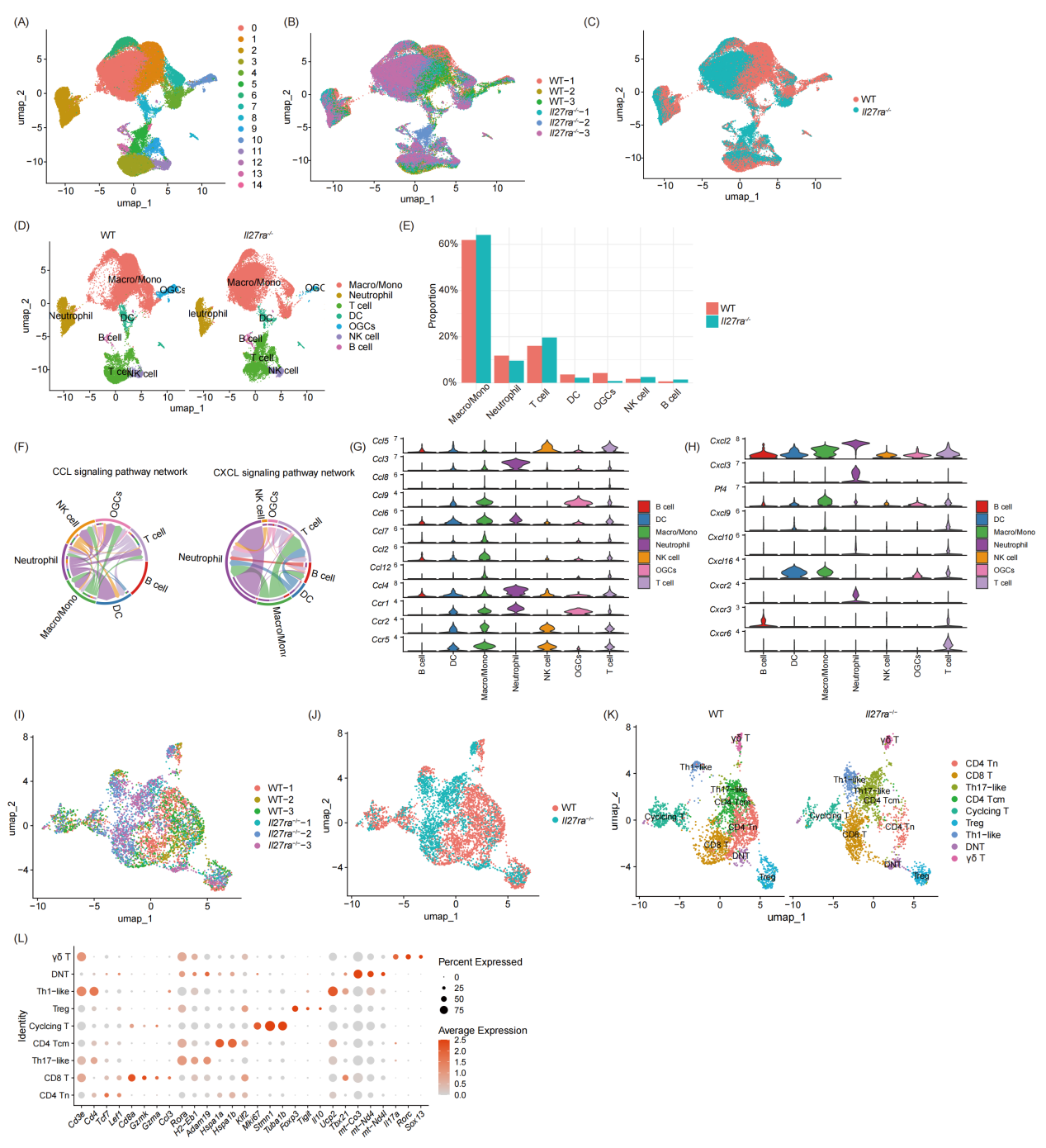


# Figure S9. Single-cell characterization of tumor-infiltrating immune cells (CD45⁺) from WT and *Il27ra⁻/⁻* mice.

# (A-D) UMAP visualizations of tumor-infiltrating CD45⁺ immune cells from WT and *Il27ra⁻/⁻* mice, showing overall clustering (A), distribution by sample (B), distribution by genotype (C), and distribution of annotated immune-cell subsets across groups (D). (E) Bar plot showing the proportions of annotated immune-cell types in WT versus *Il27ra⁻/⁻* tumors. (F) Circos plots illustrating enhanced chemokine-mediated communication among tumor-infiltrating T cells in *Il27ra⁻/⁻* mice, highlighting the CCL axis (left) and CXCL axis (right). (G-H) Violin plots showing relative expression of selected chemokine ligands and receptors in macrophage/monocyte populations interacting with T cells, focusing on the CCL axis (G) and CXCL axis (H). (I-K) UMAP plots showing reclustering of tumor-infiltrating T cells from WT and *Il27ra⁻/⁻* mice, including distribution by sample (I), distribution by genotype (J), and distribution of annotated T cell subsets (K). (L) Bubble plot displaying the expression of selected genes across major T cell subclusters.

**
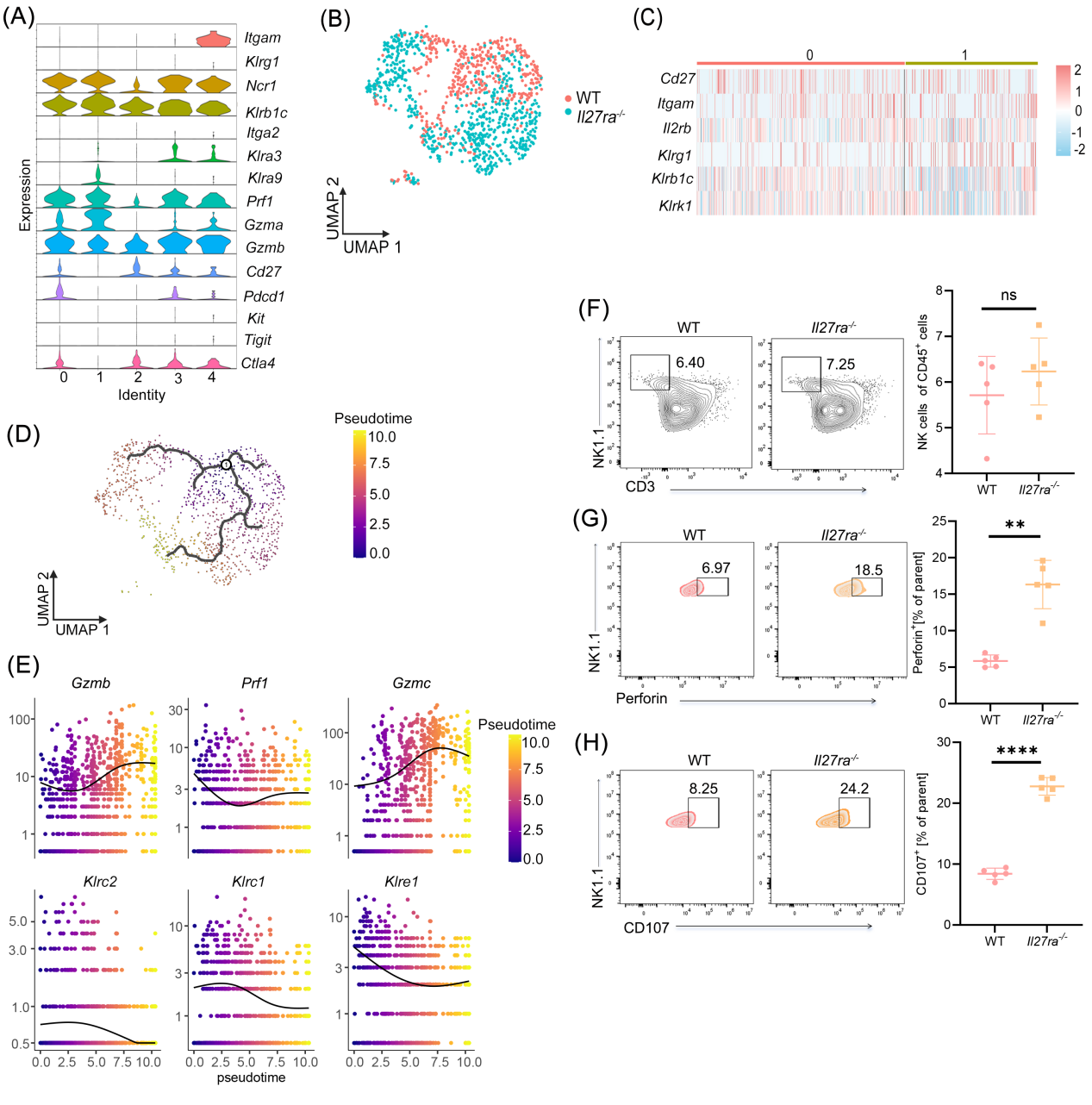
**

# Figure S10. Functional divergence of tumor-infiltrating NK cells in *Il27ra⁻/⁻* versus WT mice.

# (A) Violin plots showing the expression of key functional genes across NK-cell subclusters. (B) UMAP visualization of NK cells from WT and *Il27ra⁻/⁻* tumors, illustrating group-specific distribution patterns. (C) Heatmap displaying the expression of major maturation-associated genes across NK-cell subclusters. (D) Pseudotime trajectory analysis of NK cells revealing inferred differentiation paths. (E) Dynamic changes in cytotoxicity- and activation-related gene expression along the pseudotime trajectory. (F-H) Flow-cytometry analysis of tumor-infiltrating NK cells, showing representative histograms (left) and quantification (right) of NK-cell frequencies (F), Perforin⁺ NK-cell proportions (G), and CD107a⁺ NK-cell proportions (H) in WT versus *Il27ra⁻/⁻* mice (n=5).

# Data are presented as mean ± SD. Statistical significance was determined using two-tailed unpaired Student’s t-tests (F-H). Ns, *p* > 0.05; **p* < 0.05; ***p* < 0.01; *****p* < 0.0001.

Table 1： Clinicopathological Characteristics of Patients for Single-Cell Sequencing

| **Patient ID** | **Response Group** | **Age** | **TNM Stage (Pre-treatment)** | **Miller & Payne Grade** |
| --- | --- | --- | --- | --- |
| P1 | non-pCR | 44 | T1N1M0 | G2 |
| P2 | non-pCR | 47 | T2N2M0 | G2 |
| P3 | non-pCR | 50 | T2N2M0 | G1 |
| P4 | CR | 44 | T3N2M0 | G5 |
| P5 | CR | 52 | T2N1M0 | G5 |
| P6 | CR | 64 | T2N1M0 | G5 |

**Abbreviations List**

APP - antigen-presenting pathway

BC - breast cancer

CCIs - cell-cell interactions

CIs - confidence intervals

DAB - diaminobenzidine

DCs - dendritic cells

DEG - differential expression gene

ECL - enhanced chemiluminescence

EMR - electronic medical record

FFPE - formalin-fixed paraffin-embedded

GEO - Gene Expression Omnibus

GO - Gene Ontology

GZMB - granzyme B

HRP - horseradish peroxidase

HRs - hazard ratios

H&E - hematoxylin-eosin

ICB - immune checkpoint blockade

ICP - inhibiting immune checkpoints

ICIs - immune checkpoint inhibitors

IDC - invasive ductal carcinoma

IFN-γ - interferon-γ

IHC - immunohistochemistry

ILCs - innate lymphoid cells

IL27RA - interleukin 27 receptor alpha

KEGG - Kyoto Encyclopedia of Genes and Genomes

KM - Kaplan-Meier

KO - knockout

LDH - lactate dehydrogenase

MACS - magnetic-activated cell sorting

MHC-I - major histocompatibility complex class I

NK - natural killer

NKT - natural killer T

OS - overall survival

PCA - principal component analysis

pCR - pathological complete response

PD-L1 - programmed cell death ligand-1

PD-1 - programmed cell death protein 1

QC - quality control

RFS - recurrence-free survival

RT-qPCR - real-time quantitative reverse transcription-polymerase chain reaction

scRNA-seq - single-cell RNA sequencing

SD - standard deviation

SPF - specific pathogen-free

ST - spatial transcriptomics

TCGA - The Cancer Genome Atlas

TF - transcription factor

TILs - tumor-infiltrating lymphocytes

TMB - tumor mutation burden

TME - tumor microenvironment

TNBC - triple-negative breast cancer

UMAP - uniform manifold approximation and projection

UMI - unique molecular identifier

WB - western blotting

WT - wild type

Table S1. List of primer sequences

| Gene | Directions | Sequences |
| --- | --- | --- |
| IL27RA | Forward | TCCTTCCAGACACCGCCTTC |
|  | Reverse | TCACGACCTCCAACCCAGAAC |
| GAPDH | Forward | GGAGCGAGATCCCTCCAAAAT |
|  | Reverse | GGCTGTTGTCATACTTCTCATGG |

Table S2. IL27RA（Il27ra）knockdown/overexpression primer design

| Gene | Directions | Sequences (5′-3′) |
| --- | --- | --- |
| hIL27RA-sh | F | CCGGGCCACTGGCTACAAAGTGTATCTCGAGATACACTTTGTAGCCAGTGGCTTTTTG |
|  | R | AATTCAAAAAGCCACTGGCTACAAAGTGTATCTCGAGATACACTTTGTAGCCAGTGGC |
| mIl27ra-sh | F | CCGGCCATTCTTAGATCCTGAAGATCTCGAGATCTTCAGGATCTAAGAATGGTTTTTG |
|  | R | AATTCAAAAACCATTCTTAGATCCTGAAGATCTCGAGATCTTCAGGATCTAAGAATGG |
| hIL27RA-OE | F | CATAGAAGATTCTAGAGCTAGCGAATTCGCCACCATGCGGGGAGGCAGGG |
|  | R | CGTCATGGTCTTTGTAGTCCTCGAGGGCCAGAACCTGTGGCC |
| mIl27ra-OE | F | GAAGATTCTAGAGCTAGCGAATTCGCCACCATGAACCGGCTCCGGGTTG |
|  | R | CATGGTCTTTGTAGTCGGATCCGACTAGAAGGCCCAGCTCCTC |

Table S3. Antibody panel for flow cytometry​

| Target Antigen | Host Species | Fluorochrome | Product Number | Vendor | RRID |
| --- | --- | --- | --- | --- | --- |
| CD45 | Mouse | PE | 147712 | Biolegend | AB_2563598 |
| CD45 | Mouse | APC-Cy7 | 103116 | Biolegend | AB_312981 |
| CD3 | Mouse | PE-Cy7 | 100220 | Biolegend | AB_1732057 |
| CD3 | Mouse | AF700 | 100216 | Biolegend | AB_493697 |
| CD8 | Mouse | FITC | 100706 | Biolegend | AB_312745 |
| CD4 | Mouse | Percp-cy5.5 | 116012 | Biolegend | AB_2563023 |
| CD4 | Mouse | PE | 100408 | Biolegend | AB_312693 |
| CD11b | Mouse | FITC | 101206 | Biolegend | AB_312789 |
| CD11b | Mouse | APC | 101212 | Biolegend | AB_312795 |
| Ly-6G/Ly-6C(Gr-1) | Mouse | PE | 108407 | Biolegend | AB_313372 |
| F4/80 | Mouse | FITC | 111602 | Biolegend | AB_2927831 |
| NK1.1 | Mouse | BV421 | 108731 | Biolegend | AB_10895916 |
| Perforin | Mouse | PE | 154306 | Biolegend | AB_2721639 |
| Perforin | Mouse | APC | 154303 | Biolegend | AB_2721462 |
| GZMB | Mouse | APC | 372203 | Biolegend | AB_2687027 |
| IFNG | Mouse | PE | 505807 | Biolegend | AB_315401 |
| Zombie Aqua Fixable Viability Kit |  | BV510 | 423101 | Biolegend | Not applicable |

Table S4. Genotyping Primer Sequences​

| Type | Sequences (5'→3') |
| --- | --- |
| Wild type Forward | CAA GAC CTT GTG TGC AGG TG |
| Wild type Reverse | GTC ACC ATC TTG AGC CCA GT |
| Mutant Forward | CTT GGG TGG AGA GGC TAT TC |
| Mutant Reverse | AGG TGA GAT GAC AGG AGA TC |

Table S5 Epithelial-cell DEGs after treatment in the non-pCR group

| No. | Gene_Symbol | log2FoldChange | pvalue | Type |
| --- | --- | --- | --- | --- |
| 1 | LINC01087 | -7.340656956 | 0.0166783400480185 | down |
| 2 | AGTR1 | -5.878994756 | 0.00019844836484431 | down |
| 3 | RORB-AS1 | 5.64976129241377 | 0.00584221626591068 | up |
| 4 | AC138811.2 | 5.448789186 | 0.0098870219037839 | up |
| 5 | ISY1 | 5.448789186 | 0.0098870219037839 | up |
| 6 | IGHV3-15 | -5.3413484 | 0.00261365516067707 | down |
| 7 | AC137932.2 | 5.21392475976366 | 0.017615972802305 | up |
| 8 | LINC02225 | 5.21365530644653 | 0.00116432882870342 | up |
| 9 | AC007598.2 | 5.20230136492001 | 0.0193140258737564 | up |
| 10 | PI15 | -5.049134731 | 0.0188733670598888 | down |
| 11 | ISL1 | 4.96941110140052 | 0.00296488507308476 | up |
| 12 | MOGAT3 | 4.93032048487394 | 0.00585113351899536 | up |
| 13 | AC093583.1 | 4.77854374767116 | 0.00918319386457383 | up |
| 14 | IGLV1-40 | -4.728323267 | 0.000802428711768959 | down |
| 15 | AL611929.1 | 4.70418646299541 | 0.000668846555998562 | up |
| 16 | RNU6-1078P | 4.59494277864321 | 0.0144567249734593 | up |
| 17 | TAT | -4.458268223 | 0.00346929077980115 | down |
| 18 | U3.31 | 4.39484604748762 | 0.00345190493095773 | up |
| 19 | AC226101.1 | 4.33201044174317 | 0.0282486369620697 | up |
| 20 | U3.46 | 4.32848941617245 | 0.000566997471583897 | up |
| 21 | RNU6-1227P | 4.28172121 | 0.0171699570294461 | up |
| 22 | RNU6-99P | 4.28091271343773 | 0.0147670715518912 | up |
| 23 | GPR6 | 4.2724774739007 | 0.0143692387705869 | up |
| 24 | IGLV4-69 | -4.234749464 | 0.00729079616689999 | down |
| 25 | AL121652.1 | -4.227582776 | 0.021450023652113 | down |
| 26 | RAPGEF6 | -4.127673247 | 0.0154406981032322 | down |
| 27 | AC009511.2 | 4.09816524050764 | 0.00880589086443966 | up |
| 28 | LINC01563 | -4.054029763 | 0.00337206860346683 | down |
| 29 | GSX1 | 4.03176055601811 | 0.000386417156960621 | up |
| 30 | AC131274.1 | 4.02486240382624 | 0.0234158450729159 | up |
| 31 | LINC01963 | -3.979239077 | 0.0267495534744013 | down |
| 32 | RNU5E-9P | 3.95455445705404 | 0.00443407290489192 | up |
| 33 | RNU6-367P | 3.94956497072428 | 0.0353848551086399 | up |
| 34 | AP005436.3 | 3.9469117724947 | 0.0260113557197447 | up |
| 35 | LHX5-AS1 | -3.911951139 | 0.0276901293408974 | down |
| 36 | AC073283.3 | -3.897773925 | 0.0137428046498588 | down |
| 37 | AC010307.3 | 3.86398911397905 | 0.024959513634911 | up |
| 38 | AC079341.1 | 3.84866219864832 | 0.00153579060842532 | up |
| 39 | C11orf71 | -3.844195357 | 0.0169481993088757 | down |
| 40 | SNORD3B-1 | 3.83845382958029 | 0.0132413035109945 | up |
| 41 | LINC00672 | -3.834946799 | 0.0350867953019919 | down |
| 42 | HIF1A-AS3 | -3.834355608 | 0.000381670073544883 | down |
| 43 | AC009831.1 | 3.82204478380572 | 0.00508334412456222 | up |
| 44 | AC093591.2 | -3.790487437 | 0.0194252579628689 | down |
| 45 | AC009563.1 | 3.78948804030964 | 0.0104654314452431 | up |
| 46 | MAGEC1 | -3.786137919 | 0.0433095649883708 | down |
| 47 | AMER3 | 3.76489027003696 | 0.00035349932235275 | up |
| 48 | U3.22 | 3.75709881480957 | 0.000314721613778291 | up |
| 49 | AC090125.2 | -3.727720207 | 0.0415549244983208 | down |
| 50 | EREG | -3.715586393 | 0.0121440897093539 | down |
| 51 | GPR62 | 3.70839089391884 | 0.000377884013135619 | up |
| 52 | RNU6-1338P | 3.70561728220863 | 0.0150879483961638 | up |
| 53 | S100A7 | 3.7020004722017 | 0.0084524996117091 | up |
| 54 | CLEC4E | 3.69765154284072 | 0.00959701813656503 | up |
| 55 | AC109446.3 | 3.69064587506863 | 0.0329493422854388 | up |
| 56 | FCGR3A | -3.675012349 | 0.0475309670835457 | down |
| 57 | AL606970.2 | 3.66679993037756 | 0.0154166655656323 | up |
| 58 | AC006480.2 | -3.654925362 | 0.0208704356354858 | down |
| 59 | SNORD3J | 3.65120668667693 | 0.0469719636060541 | up |
| 60 | RN7SKP125 | 3.64236154831282 | 0.0116863753878866 | up |
| 61 | RNU4-84P | 3.63538768398833 | 0.0189388808202729 | up |
| 62 | AC092295.2 | -3.614530001 | 0.0487438607624084 | down |
| 63 | RNU4-90P | 3.614249814 | 0.00635767225051454 | up |
| 64 | IGKV1-5 | -3.613738675 | 0.0335321363348025 | down |
| 65 | AL008720.1 | 3.60324821137223 | 0.0164109231855021 | up |
| 66 | CTRB1 | 3.58853269357404 | 0.00665979800497182 | up |
| 67 | HMGCS2 | -3.580020611 | 0.0427867293216271 | down |
| 68 | AL020994.3 | 3.57027767566776 | 0.0477510029963848 | up |
| 69 | RNU5B-3P | 3.57027767566776 | 0.0477510029963848 | up |
| 70 | IGF2-AS | 3.56445003047002 | 0.0238627298318034 | up |
| 71 | RNU5D-1 | 3.55544537800274 | 0.00159987557180919 | up |
| 72 | EBI3 | 3.54872864853916 | 0.0493374654714133 | up |
| 73 | DNLZ | 3.52258545693834 | 0.0045125066927451 | up |
| 74 | SNORA22C | 3.51956594316337 | 0.00146939867400545 | up |
| 75 | RNU6-1127P | 3.49268353546348 | 0.028784416867777 | up |
| 76 | ABRA | 3.4701504499478 | 0.0261897489264299 | up |
| 77 | SLC7A2 | -3.449011678 | 0.00154382731147394 | down |
| 78 | AL359715.3 | -3.440790277 | 0.0157491470176875 | down |
| 79 | AL356737.2 | 3.44044006667553 | 0.00160406042703742 | up |
| 80 | RNU5E-4P | 3.4378796023996 | 0.00135690007657263 | up |
| 81 | ACSM4 | 3.43712970900027 | 0.0215369130269817 | up |
| 82 | GASK1B | -3.416975459 | 0.00295773237965115 | down |
| 83 | LINC01465 | 3.41145017660032 | 0.0460804246453167 | up |
| 84 | LINC01460 | 3.40936618469261 | 0.00494566001135134 | up |
| 85 | AC009052.1 | -3.398068006 | 0.0353163223148805 | down |
| 86 | ZMYM4-AS1 | 3.38476611454484 | 0.0321147028876549 | up |
| 87 | AL021368.2 | -3.37138017 | 0.00704418053746437 | down |
| 88 | FOXA1 | -3.353281786 | 0.00593777198118473 | down |
| 89 | MYOC | 3.33910739742713 | 0.0114053878810514 | up |
| 90 | OBP2B | 3.33536554106595 | 0.0120444911818632 | up |
| 91 | KRT79 | 3.325692583 | 0.0351986570522979 | up |
| 92 | AC008453.2 | 3.31493428224695 | 0.0427244932732129 | up |
| 93 | AL161431.1 | 3.31171790470284 | 0.00907742624946818 | up |
| 94 | GOLGA6L4 | -3.307845678 | 0.0431028675265851 | down |
| 95 | C1orf232 | 3.30685303871057 | 0.0262453568743885 | up |
| 96 | SALL1 | 3.30543197807828 | 0.0412461739586433 | up |
| 97 | PGR | -3.291913176 | 0.00325886475794548 | down |
| 98 | MYO16-AS1 | 3.29022441473015 | 0.00339560437803113 | up |
| 99 | AC110760.1 | -3.286596058 | 0.0223639388529653 | down |
| 100 | AL138820.1 | -3.278797251 | 0.0498483506010827 | down |
| 101 | NR4A3 | 3.2786793867675 | 0.000491405438010499 | up |
| 102 | AL606970.3 | 3.25031987103785 | 0.00910763345096122 | up |
| 103 | NGB | 3.23407028093443 | 0.0463415160427613 | up |
| 104 | AC021092.1 | 3.22938328811482 | 0.0162357911606263 | up |
| 105 | TNK2-AS1 | 3.22806090204702 | 0.00474726719244009 | up |
| 106 | ELMO1-AS1 | 3.22500501925732 | 0.0293416739208063 | up |
| 107 | RNU6-546P | 3.22367103125923 | 0.0242066300525791 | up |
| 108 | RNU4-6P | 3.22158842415836 | 0.044874883994395 | up |
| 109 | ACTA2 | -3.218633704 | 0.00116892797571527 | down |
| 110 | FAM198B-AS1 | -3.218599097 | 0.00390661942547216 | down |
| 111 | AC062032.1 | 3.206472021 | 0.0228468441167739 | up |
| 112 | APLN | 3.19885210166503 | 0.047820877480856 | up |
| 113 | AC018558.1 | 3.19324906024822 | 0.0336071756204392 | up |
| 114 | AC080129.1 | 3.17851008741493 | 0.0318468935202053 | up |
| 115 | PKDREJ | 3.17820815368523 | 0.0300872651706425 | up |
| 116 | AC241644.2 | 3.17021545790646 | 0.0462632953044493 | up |
| 117 | SNHG5 | 3.14461899442341 | 0.000128648664210664 | up |
| 118 | XBP1 | -3.14241568 | 0.000437063770390309 | down |
| 119 | SNORD81 | 3.14070486379442 | 0.000279567343029385 | up |
| 120 | TMPRSS4 | 3.13946128280283 | 0.00202771188608973 | up |
| 121 | AC010601.1 | 3.12908839056985 | 0.00113440602202665 | up |
| 122 | RNU5A-8P | 3.1252571059634 | 0.00153391551273073 | up |
| 123 | IL2RB | 3.11977136303523 | 0.000310391877560873 | up |
| 124 | AC007463.2 | 3.11701823768171 | 0.0464072191737349 | up |
| 125 | RNU4-70P | 3.11688400449075 | 0.00440587322858393 | up |
| 126 | RNU5F-4P | 3.11625040178136 | 0.00126002626647201 | up |
| 127 | SLC25A4 | -3.103212212 | 0.0228602845853682 | down |
| 128 | BARHL2 | 3.10154550152603 | 0.033373544063706 | up |
| 129 | AC244100.3 | 3.09572514767019 | 0.0346238960782631 | up |
| 130 | LINC02739 | 3.09025616698016 | 0.00082942275517742 | up |
| 131 | NEK10 | -3.089075975 | 0.00148232041023447 | down |
| 132 | ADGRA1-AS1 | 3.0821200879522 | 0.0262260147426436 | up |
| 133 | MIR663AHG | 3.06376627450049 | 0.000583357003107543 | up |
| 134 | SNORA22 | 3.05988481725232 | 0.00198571151359837 | up |
| 135 | AC002480.2 | 3.05847379962052 | 0.032673470672513 | up |
| 136 | AC011676.3 | 3.04130134376035 | 0.00415594599858629 | up |
| 137 | AC002401.1 | 3.035075195 | 0.00987438558425235 | up |
| 138 | RNU5F-1 | 3.03507465190217 | 0.00246634931718127 | up |
| 139 | H19 | 3.03228603001714 | 0.017951287161935 | up |
| 140 | SNORD3A | 3.0244612695806 | 0.00124935804062938 | up |
| 141 | UBXN10 | 3.01438534633511 | 0.0400120452833252 | up |
| 142 | CXCL10 | 2.99708488403051 | 0.00704578775441835 | up |
| 143 | RNU4-15P | 2.98434233321827 | 0.00396043715795385 | up |
| 144 | SNORD32B | 2.98391524325618 | 0.0253243388490284 | up |
| 145 | RNU4-4P | 2.98021359281958 | 0.023511473016394 | up |
| 146 | AC108134.2 | 2.97565021045532 | 0.00799686053134285 | up |
| 147 | AC092364.1 | 2.97225413059975 | 0.0212740672521435 | up |
| 148 | Z82185.1 | 2.97120693842289 | 0.0326810891754878 | up |
| 149 | AL022316.1 | 2.96252732219115 | 0.00206470835829271 | up |
| 150 | ELAVL3 | 2.961365139 | 0.00954197054055244 | up |
| 151 | LINC02214 | 2.94507718230011 | 0.0442086619311748 | up |
| 152 | AC079089.1 | 2.94058956789875 | 0.00333804123014946 | up |
| 153 | TMEM26 | -2.940141403 | 0.0166735819963958 | down |
| 154 | SLC22A12 | 2.93595809032307 | 0.0390566134590537 | up |
| 155 | ALG10B | -2.934586905 | 0.0203995288743589 | down |
| 156 | RAI1-AS1 | 2.93083182261961 | 0.0350095819844209 | up |
| 157 | MYH1 | 2.91953648854562 | 0.0435352354247442 | up |
| 158 | RN7SKP168 | 2.91777376663493 | 0.034052048958332 | up |
| 159 | KLHDC9 | 2.91457966769114 | 0.0463958407613187 | up |
| 160 | PCAT18 | -2.913929247 | 0.0425489160348496 | down |
| 161 | AL445588.1 | 2.91195360578338 | 0.00654334207439494 | up |
| 162 | GRTP1-AS1 | 2.91186246886598 | 0.0121913979297075 | up |
| 163 | SNORD3H | 2.91033160906394 | 0.0197580768217251 | up |
| 164 | AC102953.2 | 2.90949062100524 | 0.0209441713429931 | up |
| 165 | CDC14A | -2.906997547 | 0.00787820359529984 | down |
| 166 | RASSF8-AS1 | 2.90533097889034 | 0.0124118285554817 | up |
| 167 | WFDC11 | 2.90431937875652 | 0.00708241657391533 | up |
| 168 | LINC02791 | 2.90103014088814 | 0.0231407718615938 | up |
| 169 | LINC02700 | -2.894006693 | 0.027512764968775 | down |
| 170 | MIR4454 | 2.88052225055683 | 0.0277340732328518 | up |
| 171 | LIPK | 2.87956669225707 | 0.0156857834036612 | up |
| 172 | AC007406.5 | -2.871899405 | 0.00712660752775546 | down |
| 173 | HAS1 | 2.87036440524109 | 0.0474763660135774 | up |
| 174 | ALDH3A1 | 2.86769447102142 | 0.00470927183790588 | up |
| 175 | RNU6-959P | 2.86695000299415 | 0.0333415801938671 | up |
| 176 | RNU1-96P | 2.85563340771715 | 0.0211601230601499 | up |
| 177 | LYL1 | 2.85071697329728 | 0.0402148870529869 | up |
| 178 | AC093766.1 | 2.84924577912073 | 0.0397023978758456 | up |
| 179 | PROC | 2.84593127368145 | 0.023485138801217 | up |
| 180 | AC005772.1 | 2.83664055286282 | 0.0280202706540753 | up |
| 181 | AP003555.2 | -2.836347526 | 0.0167860249052427 | down |
| 182 | CARD9 | 2.83630614540736 | 0.00460612100185902 | up |
| 183 | AC242842.3 | -2.834461964 | 0.00688746750278608 | down |
| 184 | IL27RA | 2.83244355588016 | 0.00829398483730946 | up |
| 185 | AL589666.1 | -2.828411375 | 0.000899440618066765 | down |
| 186 | ANTXRL | 2.82720749807395 | 0.00206519169102614 | up |
| 187 | RNU1-80P | 2.82674305823647 | 0.0413873110539435 | up |
| 188 | TTR | 2.81976199393266 | 0.0154851799860533 | up |
| 189 | AC016910.1 | 2.81791500991443 | 0.0377349127686388 | up |
| 190 | LXN | -2.81595038 | 0.000759358793453008 | down |
| 191 | KLK2 | -2.812875695 | 0.0484212514632621 | down |
| 192 | AP000842.2 | 2.7965660624801 | 0.0192942956821582 | up |
| 193 | RNU6-842P | 2.79566557453631 | 0.0184899942404937 | up |
| 194 | LINC02265 | 2.79232437354928 | 0.0467992342629865 | up |
| 195 | LINC00972 | 2.79217642857757 | 0.00851895053295509 | up |
| 196 | PTN | -2.779914426 | 0.0113601661836461 | down |
| 197 | AC109441.1 | 2.77766757006941 | 0.0450203678561452 | up |
| 198 | TBXT | 2.7763185388878 | 0.016121138915604 | up |
| 199 | RNU6-31P | 2.77349124430489 | 0.0123471955693511 | up |
| 200 | AC062028.1 | 2.77058167326829 | 0.0033620029415925 | up |
| 201 | TNF | 2.7682806973377 | 0.0370958115636838 | up |
| 202 | AC013640.1 | 2.76109644810657 | 0.00801660430963048 | up |
| 203 | AC012485.3 | 2.76109076526378 | 0.00787958663953849 | up |
| 204 | TTPA | -2.74442145 | 0.00415041281119239 | down |
| 205 | AL845331.2 | -2.742708697 | 0.0207509670605193 | down |
| 206 | MUCL1 | -2.734370359 | 0.0159222612036651 | down |
| 207 | CD177 | 2.73320240821282 | 0.0167696445192494 | up |
| 208 | AC098679.5 | -2.731771462 | 0.0411561341550512 | down |
| 209 | AC091163.1 | 2.731288167 | 0.035585576334351 | up |
| 210 | IL20RA | -2.725107873 | 0.010434399035217 | down |
| 211 | MEMO1 | -2.723495807 | 0.0195405960369176 | down |
| 212 | MANCR | 2.71913084630839 | 0.02871077114941 | up |
| 213 | RNU6-227P | 2.71563188771674 | 0.0396944360121679 | up |
| 214 | AC125618.1 | -2.715558799 | 0.0238311552942914 | down |
| 215 | U1.13 | 2.71259493995856 | 0.00729369929923569 | up |
| 216 | RNU6-620P | 2.7079152370971 | 0.00664323335740599 | up |
| 217 | U4.6 | 2.70707529486941 | 0.0468302790576321 | up |
| 218 | AC144573.1 | 2.70651519084045 | 0.0453404838401538 | up |
| 219 | TMEM145 | 2.70199641132277 | 0.0382851872956788 | up |
| 220 | NALT1 | 2.69632979454052 | 0.0329009983244912 | up |
| 221 | CCL28 | -2.687582596 | 0.000606232355657883 | down |
| 222 | DCUN1D2-AS | 2.68679326347307 | 0.0299166817572384 | up |
| 223 | RHOH | -2.681471821 | 0.0111059894341605 | down |
| 224 | AL591845.1 | -2.681291985 | 0.00473737733012082 | down |
| 225 | AC073957.3 | 2.68031260881992 | 0.0489830492170722 | up |
| 226 | EXOC3L1 | 2.67392358733194 | 0.0490045068770542 | up |
| 227 | RNVU1-3 | 2.66255408210063 | 0.0126147959021747 | up |
| 228 | AP000766.1 | -2.662249659 | 0.012737147538797 | down |
| 229 | RN7SKP214 | 2.66073819707766 | 0.0374155016776344 | up |
| 230 | AC020909.3 | 2.65862610709036 | 0.0376142069867261 | up |
| 231 | CD200R1L-AS1 | -2.6545607 | 0.0369088263103194 | down |
| 232 | AC093627.1 | 2.65100916555239 | 0.0241819460478512 | up |
| 233 | AC037459.1 | 2.64681292784716 | 0.0209476741755079 | up |
| 234 | AC254562.2 | 2.6401880436032 | 0.0114989041407432 | up |
| 235 | ZNF385B | -2.637766275 | 0.00844650824933978 | down |
| 236 | AC008770.4 | 2.63009038602654 | 0.0111597071239153 | up |
| 237 | AC122707.1 | 2.6298162464874 | 0.00973023250350002 | up |
| 238 | KLK11 | 2.62819038464772 | 0.0148207321106296 | up |
| 239 | AL080275.1 | 2.62699617594533 | 0.0285023649199605 | up |
| 240 | BMP6 | 2.62588042070423 | 0.00674446237440528 | up |
| 241 | SPARCL1 | -2.618006188 | 0.0198281829634618 | down |
| 242 | AL021308.1 | -2.612814802 | 0.0426998021250413 | down |
| 243 | ETV2 | 2.61266213668155 | 0.0489431740674208 | up |
| 244 | RNU5B-2P | 2.6103851416729 | 0.0225869521443212 | up |
| 245 | U3.11 | 2.60646624196444 | 0.0499682970876098 | up |
| 246 | MSLN | 2.59720382267626 | 0.0114755638819341 | up |
| 247 | OPRD1 | 2.59411407280582 | 0.00380025662170928 | up |
| 248 | KYNU | -2.593505902 | 0.00394267756747395 | down |
| 249 | LINC01664 | 2.59080307197735 | 0.0358608244438909 | up |
| 250 | AC124798.3 | 2.587916765 | 0.0498043405819723 | up |
| 251 | RNU6-453P | 2.57317333164385 | 0.0410931940501751 | up |
| 252 | JCHAIN | -2.572905409 | 0.01133069281065 | down |
| 253 | HCAR1 | -2.572111162 | 0.0497256186770755 | down |
| 254 | SIDT1 | -2.571325014 | 0.0139143657270727 | down |
| 255 | AC022101.1 | -2.569854718 | 0.0370260300234064 | down |
| 256 | AC010737.1 | 2.56586190366034 | 0.0226943940946735 | up |
| 257 | MXRA8 | 2.56035045281617 | 0.00909144499070411 | up |
| 258 | CCDC194 | 2.55349241844601 | 0.0329859014964137 | up |
| 259 | ATP1B2 | 2.55107581140384 | 0.0210834080560081 | up |
| 260 | LINC02523 | 2.54680810779068 | 0.0226646173043695 | up |
| 261 | AC010086.3 | 2.53192146681947 | 0.049170417950792 | up |
| 262 | AC119150.1 | 2.52492257513489 | 0.017113656079192 | up |
| 263 | DACT2 | 2.52371345826207 | 0.0355494866910154 | up |
| 264 | AC091132.2 | 2.52192778979946 | 0.0181509975039966 | up |
| 265 | LINC01744 | 2.52095307195392 | 0.0492547849664106 | up |
| 266 | AC107027.1 | 2.5147557390535 | 0.0213373688497765 | up |
| 267 | STC2 | -2.512631116 | 0.00517932252438849 | down |
| 268 | ZIC5 | 2.51124533165144 | 0.0139386391189897 | up |
| 269 | GNG4 | 2.50723732439449 | 0.00464049215930116 | up |
| 270 | MAGEB10 | 2.50697441508767 | 0.0184543677685767 | up |
| 271 | SPDEF | -2.505969947 | 0.0193546009366691 | down |
| 272 | AL158151.3 | 2.50078335854708 | 0.0317656666174475 | up |
| 273 | VNN1 | 2.48707597605237 | 0.0308158237375979 | up |
| 274 | DNASE1L2 | 2.48702775967974 | 0.0161360922428956 | up |
| 275 | P3H3 | 2.4803042792471 | 0.0251429111571877 | up |
| 276 | RRAGA | -2.462569123 | 0.0309325587915516 | down |
| 277 | AC016730.1 | 2.45328270383781 | 0.0435173777010074 | up |
| 278 | RNU6-784P | 2.45304505626719 | 0.0223299754646541 | up |
| 279 | FSD1 | 2.45037302021964 | 0.028774791367587 | up |
| 280 | SCNN1D | 2.44661333444971 | 0.00429703069229852 | up |
| 281 | LIF | 2.4463757944557 | 0.0116326489302281 | up |
| 282 | RNY4P25 | 2.44498657163232 | 0.0331772901520167 | up |
| 283 | LRRC15 | 2.4443533179563 | 0.0103631804202358 | up |
| 284 | AL139379.1 | 2.43925542736501 | 0.0189539944690185 | up |
| 285 | AL137782.1 | -2.436769633 | 0.00956662460350907 | down |
| 286 | RN7SKP150 | 2.43191236895919 | 0.0270025102718582 | up |
| 287 | UBE2A | -2.430033396 | 0.00405773811061847 | down |
| 288 | RBPJL | 2.42837515317043 | 0.0485176522360255 | up |
| 289 | PLPPR3 | 2.42724650830339 | 0.0236367567200984 | up |
| 290 | IL2RA | 2.42106915240054 | 0.0144546594984531 | up |
| 291 | TMEM101 | -2.420979196 | 0.0202967875592817 | down |
| 292 | AC067751.1 | 2.41950948008467 | 0.0064405860217532 | up |
| 293 | RNU4-1 | 2.41889878152907 | 0.00221602570753625 | up |
| 294 | SNORD81.1 | 2.41644750808312 | 0.0187958150129099 | up |
| 295 | CACNG7 | 2.41370321611008 | 0.0282541828294214 | up |
| 296 | AL121790.1 | -2.406418493 | 0.0248544496433137 | down |
| 297 | RNU1-88P | 2.40377007932776 | 0.0113577018640586 | up |
| 298 | LINC02144 | 2.40051726309542 | 0.0191321862504089 | up |
| 299 | TBC1D9 | -2.400195655 | 0.0023110438955196 | down |
| 300 | CPA6 | -2.389065699 | 0.0320937986354008 | down |
| 301 | SPDYA | -2.386227082 | 0.00796530876091996 | down |
| 302 | DIPK1C | 2.38513142634848 | 0.043449048284998 | up |
| 303 | LINC01133 | 2.38143081706463 | 0.0115014095207149 | up |
| 304 | SAG | 2.38089056850541 | 0.0215959144831637 | up |
| 305 | KRT73-AS1 | 2.37442114046793 | 0.0369195947558401 | up |
| 306 | AC027117.2 | -2.371547078 | 0.0114980361396493 | down |
| 307 | DDX11 | 2.36649561706075 | 0.00494751369682266 | up |
| 308 | CEBPA | 2.36432403737984 | 0.0155283238112234 | up |
| 309 | AC100801.1 | -2.362972689 | 0.0433257580769231 | down |
| 310 | HES4 | 2.36094696065024 | 0.0493124554966182 | up |
| 311 | DCN | -2.358495306 | 0.0344736024861724 | down |
| 312 | TMEM144 | -2.357664433 | 0.00324084442766573 | down |
| 313 | NOL4 | 2.35583923145163 | 0.0242521496126226 | up |
| 314 | RNU6-602P | 2.35101115586108 | 0.0211028333966531 | up |
| 315 | DCDC2 | -2.33803898 | 0.00846479735345624 | down |
| 316 | ITGB6 | -2.331058269 | 0.00211139397705363 | down |
| 317 | CYP4F22 | -2.322968664 | 0.0466692780312553 | down |
| 318 | AC012404.1 | 2.32294750810833 | 0.0289135140098752 | up |
| 319 | PDE12 | -2.322611325 | 0.0228226771725245 | down |
| 320 | FAM104B | -2.319905412 | 0.0312265977406539 | down |
| 321 | RNU6-893P | 2.3129761133703 | 0.0200865385367585 | up |
| 322 | RNU4-17P | 2.31234692067519 | 0.00835616039606804 | up |
| 323 | NTN4 | -2.307816162 | 0.00502338202257643 | down |
| 324 | SNHG7 | 2.30753714965856 | 0.0044114841138374 | up |
| 325 | AC025183.2 | 2.30635650819249 | 0.0193764181329785 | up |
| 326 | ADGRD2 | -2.303279022 | 0.0475623958468539 | down |
| 327 | THSD4 | -2.301631321 | 0.00306139525987182 | down |
| 328 | PPP1R14A | 2.28383701199842 | 0.0378558641742875 | up |
| 329 | AP003778.1 | 2.27855483176535 | 0.0231632266909913 | up |
| 330 | AC092335.1 | 2.27223989565713 | 0.0203140490549213 | up |
| 331 | WDR90 | 2.26990238902431 | 0.00180349584803875 | up |
| 332 | CA2 | -2.269739933 | 0.0398731432258039 | down |
| 333 | STEAP4 | -2.265850955 | 0.0256856867487372 | down |
| 334 | RHBDL1 | 2.26112882064312 | 0.0380417429719158 | up |
| 335 | RNU6-702P | 2.25857064765537 | 0.0181609167938968 | up |
| 336 | TF | -2.257876534 | 0.0283450017596224 | down |
| 337 | OTUD6B-AS1 | -2.255541718 | 0.0139257137243409 | down |
| 338 | RNU4-42P | 2.25370074688815 | 0.0157110526869297 | up |
| 339 | VASH2 | 2.25104400332963 | 0.00959342458985916 | up |
| 340 | ZNF304 | -2.250620215 | 0.0338088376424319 | down |
| 341 | EIF3E | 2.2458200523206 | 0.0104708869732965 | up |
| 342 | TERT | 2.24522132096444 | 0.0139420187417048 | up |
| 343 | AL645608.7 | 2.2435830562942 | 0.0035449058222622 | up |
| 344 | FAM110C | -2.239285805 | 0.0374852033241157 | down |
| 345 | AC087612.1 | 2.23811581235848 | 0.0454306838907359 | up |
| 346 | C14orf177 | 2.23614812758821 | 0.0348212332096385 | up |
| 347 | OTP | 2.22993069003997 | 0.037209921115482 | up |
| 348 | RNU6ATAC | 2.22338752541372 | 0.00312227492705308 | up |
| 349 | RNU1-151P | 2.22227923319681 | 0.013894663375823 | up |
| 350 | SRI | -2.217355446 | 0.00475350103190053 | down |
| 351 | U1.6 | 2.216435132 | 0.0238762259618753 | up |
| 352 | TMC5 | -2.212942236 | 0.01805926282559 | down |
| 353 | IL6ST | -2.212526339 | 0.00500005018226893 | down |
| 354 | AC240565.1 | -2.207369581 | 0.0280109727663664 | down |
| 355 | IFI27 | 2.20536064116283 | 0.0185979831129434 | up |
| 356 | AC092683.1 | 2.19879269337867 | 0.00438596007139435 | up |
| 357 | MMP7 | -2.195958799 | 0.00724609047210002 | down |
| 358 | AL160271.2 | 2.19162210278752 | 0.0393192813715455 | up |
| 359 | LYPD6B | -2.190984384 | 0.0145719467524689 | down |
| 360 | GLI1 | 2.18814021225658 | 0.0499630239775633 | up |
| 361 | AP000696.1 | 2.17863272871289 | 0.0485931805778742 | up |
| 362 | HOMER3-AS1 | 2.1754215588515 | 0.0483059000133534 | up |
| 363 | FAM199X | -2.173335767 | 0.00678286124873721 | down |
| 364 | GCNA | -2.169177973 | 0.0243656315562415 | down |
| 365 | MLLT3 | -2.168649456 | 0.0130503333732353 | down |
| 366 | MTUS1 | -2.157128748 | 0.00825210253306344 | down |
| 367 | SUSD5 | 2.15625399620534 | 0.00858233175497519 | up |
| 368 | CYP2D6 | 2.15515824972781 | 0.0156220177132433 | up |
| 369 | LTF | -2.152532286 | 0.00528712898756778 | down |
| 370 | RNU1-22P | 2.1518181574957 | 0.0404345463208905 | up |
| 371 | OSTM1-AS1 | 2.15075029310794 | 0.0466110729703382 | up |
| 372 | AC125232.2 | -2.147556283 | 0.014587008783969 | down |
| 373 | HLA-DQB2 | 2.14699465651857 | 0.0336565312580117 | up |
| 374 | C10orf105 | 2.14517353585369 | 0.0460211448171535 | up |
| 375 | RNU1-148P | 2.14253071750497 | 0.0166906846840922 | up |
| 376 | FFAR4 | 2.14094948667881 | 0.0489854498747565 | up |
| 377 | TONSL | 2.14028280851672 | 0.00755868294884398 | up |
| 378 | AC091806.1 | 2.1390277352199 | 0.0285654750330628 | up |
| 379 | MICALL2 | 2.13735248533019 | 0.00743972141626738 | up |
| 380 | AL157702.2 | 2.13712418869239 | 0.0326766991056496 | up |
| 381 | AC104806.2 | -2.135197407 | 0.00310011423454409 | down |
| 382 | RNU1-98P | 2.13386890327353 | 0.0162177692310276 | up |
| 383 | LINC00504 | -2.130240272 | 0.0141051086373805 | down |
| 384 | LINC02145 | -2.129129723 | 0.0374311793011593 | down |
| 385 | RNU6-589P | 2.12135496202701 | 0.0434581975321006 | up |
| 386 | AL078590.2 | 2.11710332210559 | 0.010112644214584 | up |
| 387 | NMUR2 | 2.10809865925664 | 0.0391694229702313 | up |
| 388 | AP001993.1 | 2.10653774448789 | 0.0109694654078932 | up |
| 389 | HACD4 | -2.104990633 | 0.0396884892408603 | down |
| 390 | PBX3 | -2.099986115 | 0.00699379245413179 | down |
| 391 | PTOV1-AS2 | 2.09941032363303 | 0.0162806250445185 | up |
| 392 | AL357558.2 | 2.09716045005175 | 0.0352662205715154 | up |
| 393 | VASH1 | 2.09600642672323 | 0.0414952972673117 | up |
| 394 | AC011530.1 | 2.09233588427065 | 0.0334074283926788 | up |
| 395 | RNU1-7P | 2.09174897864083 | 0.0326451332801565 | up |
| 396 | B4GALNT3 | 2.08485889961571 | 0.0136196502722211 | up |
| 397 | LMNTD1 | 2.08475917528851 | 0.0223715292879554 | up |
| 398 | SNORD13 | 2.08246239 | 0.0110231943857106 | up |
| 399 | OLFML3 | 2.07408517219258 | 0.0478063767003931 | up |
| 400 | DBET | 2.07395382974389 | 0.0155836645517835 | up |
| 401 | NEURL1-AS1 | 2.06835368589508 | 0.0468928737293181 | up |
| 402 | RAP2A | -2.067467327 | 0.00510197986842147 | down |
| 403 | LINC01923 | 2.06684745045699 | 0.0419445994092214 | up |
| 404 | PDE1C | -2.066452799 | 0.00622496032587212 | down |
| 405 | GALNTL5 | 2.06640190881879 | 0.0293446912502043 | up |
| 406 | RXYLT1 | -2.056298827 | 0.0100775887078574 | down |
| 407 | NRIP1 | -2.053578509 | 0.0073481356944837 | down |
| 408 | SLC26A5 | 2.04107758987764 | 0.0358236647713338 | up |
| 409 | CDT1 | 2.03755784198868 | 0.0188959075861424 | up |
| 410 | SLC22A31 | 2.03727579243315 | 0.0409490011096435 | up |
| 411 | SYT6 | 2.03695016323338 | 0.0169583717206389 | up |
| 412 | CTNND2 | -2.03471783 | 0.0138674245083727 | down |
| 413 | LINC01609 | 2.03138505634805 | 0.00915727513576038 | up |
| 414 | AC093525.6 | 2.02775037705213 | 0.0312146345028859 | up |
| 415 | EAF2 | -2.022427106 | 0.0224289494193108 | down |
| 416 | LINC01597 | 2.0217736479835 | 0.0384289806023144 | up |
| 417 | CCNH | -2.020352392 | 0.00079864627817383 | down |
| 418 | PNKP | 2.01989651798565 | 0.0170307759926556 | up |
| 419 | GAD2 | 2.01790658002172 | 0.0358468556469034 | up |
| 420 | CIITA | 2.01787195661391 | 0.0245636033365978 | up |
| 421 | ARHGEF19 | 2.01251064269315 | 0.0362819702328825 | up |
| 422 | ONECUT2 | 2.0121506666204 | 0.0178846341772811 | up |
| 423 | ITPR1 | -2.010576943 | 0.0199046545137546 | down |
| 424 | RNVU1-15 | 2.00615353029904 | 0.0145584460145085 | up |
| 425 | NR4A1 | 2.00274146832458 | 0.0187715510810988 | up |
| 426 | AC010168.2 | -1.995432982 | 0.0185448232364105 | down |
| 427 | RAB30-DT | -1.993728216 | 0.00450391007748336 | down |
| 428 | MRPL47 | -1.992905887 | 0.0245123089613032 | down |
| 429 | RNU6ATAC40P | 1.9917868228693 | 0.0467851056821345 | up |
| 430 | TELO2 | 1.98992604326528 | 0.00818063262753563 | up |
| 431 | KCNMB4 | 1.98882990621757 | 0.0228944161553651 | up |
| 432 | ANKMY2 | -1.984909335 | 0.00289037565422 | down |
| 433 | FAM189A2 | -1.981234377 | 0.00879036508189321 | down |
| 434 | AC108749.1 | 1.97949928451751 | 0.0186622827701351 | up |
| 435 | RN7SKP216 | 1.97254496239824 | 0.0466515188971243 | up |
| 436 | MMGT1 | -1.971261012 | 0.0403797997830063 | down |
| 437 | LINC01962 | 1.96793493871595 | 0.0360212447040882 | up |
| 438 | GNPDA2 | -1.967928687 | 0.0113707140895126 | down |
| 439 | AC044810.3 | -1.967920897 | 0.0283897836113999 | down |
| 440 | AL132780.1 | 1.96744331982125 | 0.0181004534597707 | up |
| 441 | SAPCD2 | 1.96232502998085 | 0.0214971910117438 | up |
| 442 | AC116534.1 | 1.96212411907804 | 0.0185323943990572 | up |
| 443 | MTRNR2L11 | -1.95936938 | 0.0312828198467263 | down |
| 444 | RHPN1 | 1.95825474706444 | 0.00437299341030879 | up |
| 445 | RNVU1-7 | 1.95022346909397 | 0.0311923930742827 | up |
| 446 | FBXL13 | 1.95011348663003 | 0.0210912618388939 | up |
| 447 | RDH10 | -1.947965941 | 0.0374883816924192 | down |
| 448 | ASB18 | 1.94170206813132 | 0.0364577290389996 | up |
| 449 | LINC02306 | -1.94132033 | 0.0403006075461421 | down |
| 450 | MIR548XHG | 1.94034290550141 | 0.0268394620480609 | up |
| 451 | FGF7 | -1.938877131 | 0.0142306072856486 | down |
| 452 | HGF | 1.93844755055675 | 0.0326169798010014 | up |
| 453 | LINC02840 | 1.93559493413066 | 0.0204979841296896 | up |
| 454 | ALG13 | -1.93301794 | 0.00487212432933585 | down |
| 455 | KLHL17 | 1.93164445538155 | 0.00915291325732096 | up |
| 456 | TSHZ2 | -1.929694386 | 0.031168721954993 | down |
| 457 | ESPN | 1.92384361171736 | 0.0155408074366231 | up |
| 458 | GABRP | -1.921131747 | 0.0173027109812568 | down |
| 459 | NDUFS7 | 1.92048217210148 | 0.00873502317261843 | up |
| 460 | ABCA6 | -1.918621335 | 0.0459294655934387 | down |
| 461 | IRS1 | -1.917546892 | 0.00744619394484331 | down |
| 462 | RNU4-10P | 1.91394730365832 | 0.0268376799734885 | up |
| 463 | RNU4-7P | 1.91055576706743 | 0.0212699707801179 | up |
| 464 | RNU4-76P | 1.90778413245019 | 0.029736609932471 | up |
| 465 | AL450267.2 | 1.90729412894451 | 0.0310171887966253 | up |
| 466 | TENT5B | 1.90702117718113 | 0.0476224166863492 | up |
| 467 | FGF5 | 1.90481737299871 | 0.0291128181406248 | up |
| 468 | AC099066.2 | 1.90208088352629 | 0.0173836817321473 | up |
| 469 | AC010976.1 | 1.90203241829201 | 0.0361282838643608 | up |
| 470 | GTF2H5 | -1.901690332 | 0.0238191702911566 | down |
| 471 | MAP3K20-AS1 | 1.90125031495887 | 0.033448078956708 | up |
| 472 | AC109927.1 | -1.896722192 | 0.0315189342979633 | down |
| 473 | FAM214A | -1.889708061 | 0.00423238068827245 | down |
| 474 | RNU4-85P | 1.88944252827856 | 0.0312198898294983 | up |
| 475 | CEL | 1.88582930021104 | 0.0270997912672174 | up |
| 476 | AL390719.2 | 1.88188979398541 | 0.0203436848895379 | up |
| 477 | GUCY1A1 | -1.875674662 | 0.00953598105219292 | down |
| 478 | CHTF18 | 1.87554170410117 | 0.0172412944601068 | up |
| 479 | GLB1L3 | 1.87364000352088 | 0.0236321731473103 | up |
| 480 | AC019117.1 | 1.8704758116449 | 0.0437592656765129 | up |
| 481 | RNU1-1 | 1.86913942585364 | 0.0225076453764797 | up |
| 482 | CNGB3 | 1.86698677568702 | 0.03329067620847 | up |
| 483 | RNU4-73P | 1.86178202252234 | 0.0308354851626727 | up |
| 484 | SSBP2 | -1.858914577 | 0.0108768482840562 | down |
| 485 | FMO2 | -1.855909721 | 0.0464587369482442 | down |
| 486 | HFM1 | 1.85330341256202 | 0.00325871294199065 | up |
| 487 | NPHP3-ACAD11 | -1.848266732 | 0.0410084892338978 | down |
| 488 | ZMAT3 | -1.843876119 | 0.0183749232804519 | down |
| 489 | CEMIP | 1.84342866586984 | 0.0103616044622335 | up |
| 490 | GMPR | 1.84213212268062 | 0.0283482817235804 | up |
| 491 | SNRNP48 | -1.841421491 | 0.025846341192099 | down |
| 492 | TRMT1 | 1.84120013555202 | 0.0311253121420765 | up |
| 493 | TRMT1L | -1.839786627 | 0.0123870211887885 | down |
| 494 | ASH1L-AS1 | 1.83766528154636 | 0.0414379120765074 | up |
| 495 | AJM1 | 1.83759512111894 | 0.0184095359988077 | up |
| 496 | ABHD17B | -1.832399202 | 0.0123412130339657 | down |
| 497 | AC114956.3 | -1.829168717 | 0.0444224546040028 | down |
| 498 | TMEM240 | 1.82719321935837 | 0.0316831603110542 | up |
| 499 | MYBPHL | 1.82677408516308 | 0.0422422774002706 | up |
| 500 | AC015660.1 | 1.8251340998008 | 0.0482027704101261 | up |
| 501 | ZMAT1 | -1.824820999 | 0.0339504718105193 | down |
| 502 | BBIP1 | -1.824745016 | 0.0197416895069606 | down |
| 503 | EPS8 | -1.816326992 | 0.00492715278801675 | down |
| 504 | MAPK15 | 1.81561260951342 | 0.0337627489348884 | up |
| 505 | FAM98A | -1.815610943 | 0.0276124167037627 | down |
| 506 | SVOP | 1.81448187896619 | 0.0407532402859108 | up |
| 507 | GALNT15 | 1.81307064284462 | 0.038739225422055 | up |
| 508 | HR | 1.81238527569508 | 0.0256567269430657 | up |
| 509 | SYTL4 | -1.809621021 | 0.0213242288800879 | down |
| 510 | RECQL4 | 1.80791631602307 | 0.0219880047738019 | up |
| 511 | NRCAM | 1.80345386186015 | 0.0308701060112731 | up |
| 512 | KLF16 | 1.80231720344632 | 0.0258023072117297 | up |
| 513 | CEMIP2 | -1.801526767 | 0.0227680044612526 | down |
| 514 | RNU1-136P | 1.79756325188757 | 0.0456336521355369 | up |
| 515 | SRD5A3 | -1.791088163 | 0.0271985285285152 | down |
| 516 | NUBP2 | 1.78671398902799 | 0.0421340476369462 | up |
| 517 | NLGN4X | -1.786130571 | 0.0321249262504489 | down |
| 518 | AC093157.1 | -1.784812914 | 0.00946223728052615 | down |
| 519 | RNF126 | 1.78428449367146 | 0.0113572977890453 | up |
| 520 | GPRC5D-AS1 | -1.781470594 | 0.0346735116958759 | down |
| 521 | PSG8 | 1.77905241208391 | 0.0488069378766386 | up |
| 522 | AC012459.1 | 1.77774508522353 | 0.0495326161128543 | up |
| 523 | SEC14L2 | 1.77724642056671 | 0.0293559241852379 | up |
| 524 | FAM8A1 | -1.776693336 | 0.026213603267086 | down |
| 525 | SLC39A6 | -1.775742838 | 0.0114490263852076 | down |
| 526 | CPEB2 | -1.775452867 | 0.0129883232211728 | down |
| 527 | LINC00342 | 1.77274307613871 | 0.00408251863694194 | up |
| 528 | RPUSD1 | 1.77168972922627 | 0.0408864158275732 | up |
| 529 | AC016042.1 | 1.77146959790183 | 0.040157906972366 | up |
| 530 | DUOXA2 | 1.77066558063919 | 0.0456726912421051 | up |
| 531 | NEO1 | -1.767860243 | 0.0342933467190934 | down |
| 532 | U1.10 | 1.76769993169172 | 0.0237104856099969 | up |
| 533 | COL4A1 | 1.76655346430164 | 0.0313727412106445 | up |
| 534 | NAV1 | 1.76307230441626 | 0.0334427772162959 | up |
| 535 | PIGQ | 1.76151706238385 | 0.00969658718051645 | up |
| 536 | RNU4-66P | 1.7578898760128 | 0.0482503333936063 | up |
| 537 | TM7SF2 | 1.75749292724969 | 0.0283758362026708 | up |
| 538 | CRNDE | -1.756554273 | 0.0127826546902883 | down |
| 539 | ITGAX | 1.75632409816578 | 0.0354459292534253 | up |
| 540 | LINC02325 | 1.75371096618287 | 0.0268177464099127 | up |
| 541 | NDUFB5 | -1.750666452 | 0.0429689071745872 | down |
| 542 | RNU4-45P | 1.74959082731987 | 0.0458224770381823 | up |
| 543 | MTX2 | -1.748706597 | 0.0120281408416495 | down |
| 544 | RYBP | -1.746847852 | 0.0151018390994952 | down |
| 545 | RPL15 | -1.746487082 | 0.013594734906635 | down |
| 546 | KCTD2 | 1.74505933965163 | 0.0236477580862815 | up |
| 547 | AP5Z1 | 1.74273401874866 | 0.0257636836417636 | up |
| 548 | RNU1-18P | 1.73969061424433 | 0.0335729552595145 | up |
| 549 | RFX8 | 1.73888605800536 | 0.0351236605840346 | up |
| 550 | CDYL2 | -1.736684278 | 0.0232423900747382 | down |
| 551 | AC002985.1 | 1.73408096991595 | 0.0455961603542548 | up |
| 552 | XRCC3 | 1.73080590667152 | 0.0225334395567893 | up |
| 553 | RNU4-8P | 1.72681032384173 | 0.0349518022826792 | up |
| 554 | DDX51 | 1.72603258908084 | 0.0386387146097046 | up |
| 555 | WDR97 | 1.72403145729212 | 0.0231104269138463 | up |
| 556 | GRM5 | 1.72367979571728 | 0.0431488182088772 | up |
| 557 | SLC25A46 | -1.722470698 | 0.0347398321883289 | down |
| 558 | TRPS1 | -1.71891923 | 0.0292661734552348 | down |
| 559 | KRBOX4 | -1.718663215 | 0.0459498341021468 | down |
| 560 | MCF2L | 1.71811408264054 | 0.0341726883892787 | up |
| 561 | KLF8 | -1.714258418 | 0.0365829467541718 | down |
| 562 | PRKAA2 | -1.713301882 | 0.0175912225487639 | down |
| 563 | NDUFV2-AS1 | -1.706667831 | 0.0328726887991947 | down |
| 564 | AC004158.1 | 1.70520820828071 | 0.0435733929326389 | up |
| 565 | GAS5 | 1.70379091881438 | 0.0290956321448245 | up |
| 566 | TMEM267 | -1.700739278 | 0.0205523772489734 | down |
| 567 | SH3GLB1 | -1.692685992 | 0.0127132378915922 | down |
| 568 | MROH6 | 1.68925426471109 | 0.0234014270550524 | up |
| 569 | GSTCD | -1.687952049 | 0.0126135588691574 | down |
| 570 | ARL15 | -1.687284185 | 0.0112122853242424 | down |
| 571 | MTRNR2L6 | -1.686679901 | 0.0342976365860797 | down |
| 572 | SELENOF | -1.685438621 | 0.0150227454795575 | down |
| 573 | AC127526.2 | -1.683514055 | 0.0368599942670652 | down |
| 574 | AC018467.1 | 1.68313611137079 | 0.0393637635232037 | up |
| 575 | TMEM201 | 1.68263179591468 | 0.0116199796847195 | up |
| 576 | RNU4-2 | 1.68184297143668 | 0.0207633678928849 | up |
| 577 | CUTC | -1.680630421 | 0.0348773818704532 | down |
| 578 | PNRC2 | -1.678752248 | 0.0313359423659975 | down |
| 579 | GALNT7 | -1.676512614 | 0.0146985752027547 | down |
| 580 | TSEN15 | -1.675843513 | 0.0243361742787101 | down |
| 581 | FOXN2 | -1.672551473 | 0.0420765920887338 | down |
| 582 | SLCO4A1 | 1.67245538956902 | 0.0472470709024406 | up |
| 583 | ADAM8 | 1.6709116889072 | 0.0442262400622147 | up |
| 584 | RHOT2 | 1.66906617335467 | 0.0225318777406226 | up |
| 585 | TENT2 | -1.665345561 | 0.00553848193751161 | down |
| 586 | PIK3R1 | -1.662886942 | 0.0126928400265365 | down |
| 587 | PBX4 | 1.66230638288461 | 0.0409537257252413 | up |
| 588 | VWA5A | -1.658781402 | 0.0425129354832644 | down |
| 589 | HELZ2 | 1.65743828318733 | 0.0372068653566573 | up |
| 590 | TSC22D2 | -1.655970919 | 0.0249649989779923 | down |
| 591 | SEMA6C | 1.65586541013038 | 0.0341609663255901 | up |
| 592 | CHMP2B | -1.654799052 | 0.0307325207469702 | down |
| 593 | R3HCC1L | -1.654651334 | 0.00858325585786992 | down |
| 594 | AC114763.1 | -1.651003781 | 0.0202293029186564 | down |
| 595 | TMEM161B | -1.647746371 | 0.0122437519079662 | down |
| 596 | SLC25A36 | -1.642905802 | 0.0126757947832027 | down |
| 597 | ARMCX5-GPRASP2 | -1.641196457 | 0.0156430981818608 | down |
| 598 | INTS1 | 1.64112742248759 | 0.0432205356750478 | up |
| 599 | FEM1C | -1.638406581 | 0.021198749177317 | down |
| 600 | ORC4 | -1.637479256 | 0.0123619568305423 | down |
| 601 | TROAP | 1.63561819129355 | 0.0323718284984247 | up |
| 602 | CLOCK | -1.635138886 | 0.0178829523559272 | down |
| 603 | AC015522.1 | 1.63505281350202 | 0.0441652923107749 | up |
| 604 | LDHA | -1.634610041 | 0.0146423823599116 | down |
| 605 | CCSER1 | -1.634548055 | 0.016848612801175 | down |
| 606 | RMDN2-AS1 | 1.63281546088452 | 0.0489258166510025 | up |
| 607 | AC138969.1 | 1.63260369262317 | 0.0240630866183508 | up |
| 608 | AC093525.7 | 1.62709035617018 | 0.0413164170361369 | up |
| 609 | PCNP | -1.625421617 | 0.02886820834794 | down |
| 610 | KBTBD3 | -1.624535443 | 0.0419295218726406 | down |
| 611 | WASL | -1.621928066 | 0.0159947121756359 | down |
| 612 | SPTBN5 | 1.62047909841515 | 0.0328157545281144 | up |
| 613 | C2orf27A | -1.619839581 | 0.0172906580983001 | down |
| 614 | TMEM59 | -1.619238436 | 0.0114373956775613 | down |
| 615 | ARL6IP5 | -1.618793834 | 0.018410282400273 | down |
| 616 | ALCAM | -1.618329914 | 0.0209493057215483 | down |
| 617 | INPP5E | 1.61756758057136 | 0.0392525328299433 | up |
| 618 | PTP4A2 | -1.616610364 | 0.0225605701881039 | down |
| 619 | AC008514.1 | -1.612177328 | 0.0354601247195985 | down |
| 620 | PGRMC2 | -1.611940529 | 0.0443361779399638 | down |
| 621 | ABLIM2 | 1.61122203479779 | 0.0229873207237615 | up |
| 622 | EPB41L5 | -1.598127679 | 0.0250561967117575 | down |
| 623 | SKAP1 | -1.596519186 | 0.0165866981602138 | down |
| 624 | TBC1D8B | -1.595896463 | 0.0467122971895473 | down |
| 625 | MRTO4 | 1.59578735498326 | 0.0434744718382718 | up |
| 626 | CAPN15 | 1.59187987813056 | 0.0122778407665644 | up |
| 627 | SESN1 | -1.589710572 | 0.014081492297217 | down |
| 628 | MRPL13 | -1.585575214 | 0.0404299387595028 | down |
| 629 | SNX2 | -1.581052536 | 0.0178137485646478 | down |
| 630 | IKZF2 | -1.578102898 | 0.0121317288171465 | down |
| 631 | PRR7 | 1.57701127680384 | 0.0442655161821191 | up |
| 632 | LINC00641 | -1.575187579 | 0.0289523480904103 | down |
| 633 | ACVR2A | -1.572923205 | 0.0202444128489411 | down |
| 634 | ARFIP1 | -1.56769685 | 0.0182930165336347 | down |
| 635 | SESTD1 | -1.56649959 | 0.0497130694089696 | down |
| 636 | SNAPC4 | 1.56544981379969 | 0.0329274254711091 | up |
| 637 | LINC01885 | 1.56415999786466 | 0.0490441417562122 | up |
| 638 | TMEM106B | -1.562220833 | 0.0290771708203127 | down |
| 639 | DVL1 | 1.55995266979939 | 0.0365348259749654 | up |
| 640 | CPEB4 | -1.554159485 | 0.0119231877201675 | down |
| 641 | AL157886.1 | 1.55392601293563 | 0.0278465827188017 | up |
| 642 | FBXL6 | 1.55289106778865 | 0.0321212835362685 | up |
| 643 | DENND1B | -1.552133004 | 0.0350956222057497 | down |
| 644 | NAALADL2 | -1.548017723 | 0.0121803608383762 | down |
| 645 | APOOL | -1.544068779 | 0.0425247911298317 | down |
| 646 | LIMA1 | -1.53952441 | 0.0416252462042222 | down |
| 647 | TRMT13 | -1.538396325 | 0.0273405808756991 | down |
| 648 | AC118549.1 | -1.53757368 | 0.012010720369367 | down |
| 649 | RAB3IP | -1.536871365 | 0.0465831112949934 | down |
| 650 | AC010618.3 | 1.53520907567752 | 0.0488523700129908 | up |
| 651 | MTRNR2L10 | -1.534620968 | 0.0450377670454552 | down |
| 652 | BOP1 | 1.53443141703656 | 0.0248556636046704 | up |
| 653 | DCAF16 | -1.53419473 | 0.0265770376043242 | down |
| 654 | KIFAP3 | -1.5325115 | 0.0119327012534549 | down |
| 655 | EEF1D | 1.53059395561114 | 0.0239052601599869 | up |
| 656 | FBXL3 | -1.528951273 | 0.0363644518369283 | down |
| 657 | AL359762.3 | -1.526289207 | 0.0207285415943681 | down |
| 658 | UCHL5 | -1.525802178 | 0.0411628082554163 | down |
| 659 | IMMP1L | -1.52094399 | 0.0139762090665938 | down |
| 660 | FMO4 | -1.518686683 | 0.0418011979683587 | down |
| 661 | MCRIP2 | 1.51831452203686 | 0.0260458354373426 | up |
| 662 | SEMA6A | -1.513874064 | 0.0334251140972527 | down |
| 663 | MRPS30-DT | -1.513410643 | 0.0423136356196737 | down |
| 664 | AC005261.1 | -1.512240917 | 0.040148240204509 | down |
| 665 | MORF4L2 | -1.51079997 | 0.0355418046304815 | down |
| 666 | SEC22A | -1.507209705 | 0.0342061471972229 | down |
| 667 | MYO6 | -1.506229084 | 0.012695550984207 | down |
| 668 | PDK1 | -1.505082994 | 0.0419748962888354 | down |
| 669 | MTRNR2L3 | -1.502782008 | 0.0365234818424653 | down |
| 670 | MCCC2 | -1.499866688 | 0.036500093378891 | down |
| 671 | SEC22B | -1.49799384 | 0.0431540589143702 | down |
| 672 | TMEM135 | -1.497641962 | 0.0252377204052326 | down |
| 673 | FAM126A | -1.496679016 | 0.0119263812443146 | down |
| 674 | MCM5 | 1.49510663421445 | 0.0379873953977771 | up |
| 675 | SCN2A | -1.490170647 | 0.0315355432982716 | down |
| 676 | KRCC1 | -1.489964828 | 0.0381211627422247 | down |
| 677 | CNOT2 | -1.489830761 | 0.0132373647407825 | down |
| 678 | FBXW7 | -1.489041274 | 0.0195614298526829 | down |
| 679 | COL4A5 | -1.487829395 | 0.0184568456482023 | down |
| 680 | LINC01315 | 1.48647536771463 | 0.041248232522267 | up |
| 681 | COBLL1 | -1.486222534 | 0.0237514905361237 | down |
| 682 | RORA | -1.480014882 | 0.0410671645251244 | down |
| 683 | TMEM167A | -1.476963574 | 0.0345479945591497 | down |
| 684 | ANO8 | 1.4745491607625 | 0.0339460027344438 | up |
| 685 | DGKQ | 1.47389507410834 | 0.049791145383208 | up |
| 686 | PLEKHA5 | -1.471455462 | 0.0126404391920751 | down |
| 687 | YAE1 | -1.469963466 | 0.0495198362497984 | down |
| 688 | CHM | -1.469718276 | 0.0242955689050633 | down |
| 689 | NDUFA5 | -1.469518168 | 0.0407662903755292 | down |
| 690 | SHOC2 | -1.463559947 | 0.0190872963380168 | down |
| 691 | RNF6 | -1.461256935 | 0.0313723287282288 | down |
| 692 | FBXO30 | -1.457542232 | 0.0488236008735345 | down |
| 693 | AC092164.1 | -1.457021232 | 0.0491034324406085 | down |
| 694 | LINC01010 | 1.45067607837591 | 0.0397359762083116 | up |
| 695 | SREK1IP1 | -1.450664851 | 0.0378675249966411 | down |
| 696 | MIER1 | -1.449408839 | 0.0226774314030191 | down |
| 697 | BBX | -1.449101091 | 0.0130157900735971 | down |
| 698 | COX20 | -1.448989484 | 0.0375674384265113 | down |
| 699 | MEGF9 | -1.448344516 | 0.0328596717419782 | down |
| 700 | PRKACB | -1.447684173 | 0.0423838332851749 | down |
| 701 | C21orf58 | 1.44718388753073 | 0.0466005261815377 | up |
| 702 | HIF1A | -1.44717212 | 0.0282593912454835 | down |
| 703 | DMWD | 1.44614466211915 | 0.0480122441581424 | up |
| 704 | TTC19 | -1.442135434 | 0.0375721413801886 | down |
| 705 | TFG | -1.441880663 | 0.0378210627877936 | down |
| 706 | SLC10A7 | -1.438382786 | 0.0308186916123445 | down |
| 707 | GCA | -1.438229195 | 0.0439251920772701 | down |
| 708 | STXBP3 | -1.43570393 | 0.0411443229395995 | down |
| 709 | AP1AR | -1.435394265 | 0.0355018705523312 | down |
| 710 | MTHFD2L | -1.434412738 | 0.0171603758001653 | down |
| 711 | ISG15 | 1.43101297229236 | 0.035619042268572 | up |
| 712 | BRSK2 | 1.4271841818613 | 0.037460147052389 | up |
| 713 | BTBD3 | -1.426437386 | 0.0328068237753012 | down |
| 714 | SCRIB | 1.42564275476185 | 0.032327207313668 | up |
| 715 | AC005225.2 | 1.42473972178091 | 0.0312387614719516 | up |
| 716 | SMIM14 | -1.417786755 | 0.0377028202049047 | down |
| 717 | SPOPL | -1.414848285 | 0.026722486221505 | down |
| 718 | SLAIN2 | -1.409489257 | 0.0322966539213245 | down |
| 719 | ACTR10 | -1.403954034 | 0.04908039115294 | down |
| 720 | NR3C1 | -1.399057113 | 0.0258902390832276 | down |
| 721 | AC007038.1 | -1.395182436 | 0.0446809805871151 | down |
| 722 | CRTC1 | 1.39478853623602 | 0.0432590724533731 | up |
| 723 | CYB5B | -1.394571742 | 0.0484153696024869 | down |
| 724 | MRPL3 | -1.39091164 | 0.0306991810374186 | down |
| 725 | EDEM3 | -1.389218525 | 0.0343509800035881 | down |
| 726 | DCP2 | -1.386728674 | 0.0341006022714406 | down |
| 727 | ARL6IP6 | -1.386488367 | 0.044737879700299 | down |
| 728 | DDX10 | -1.378419092 | 0.048555944816705 | down |
| 729 | FAR1 | -1.378153278 | 0.0278679740227958 | down |
| 730 | EME2 | 1.37593016992092 | 0.0386917392080684 | up |
| 731 | TSPAN13 | -1.373538254 | 0.0371793299972612 | down |
| 732 | ZNF280C | -1.371353538 | 0.0465503899126238 | down |
| 733 | COL5A2 | -1.365373435 | 0.037653441011962 | down |
| 734 | ZNF69 | 1.36430651193084 | 0.0436091940902847 | up |
| 735 | CACNA1B | 1.36323903212298 | 0.0481301438164915 | up |
| 736 | DOT1L | 1.35991891955567 | 0.0318719147068952 | up |
| 737 | ZNF598 | 1.35508518079261 | 0.0329567364827407 | up |
| 738 | SH3BGRL | -1.350872027 | 0.0392697663306618 | down |
| 739 | HSF1 | 1.34832389815319 | 0.0433785196399269 | up |
| 740 | FZR1 | 1.34719909267065 | 0.0309503057990148 | up |
| 741 | TNPO1 | -1.345999661 | 0.0228254442918945 | down |
| 742 | FRS2 | -1.344551838 | 0.0336143806348055 | down |
| 743 | UBALD1 | 1.34344129492945 | 0.044130896072062 | up |
| 744 | AEBP2 | -1.343119906 | 0.046423633428432 | down |
| 745 | PDCD4 | -1.338249348 | 0.0353286957793545 | down |
| 746 | PCGF5 | -1.335350665 | 0.0342883464985294 | down |
| 747 | MAP3K1 | -1.333593942 | 0.0461977187929807 | down |
| 748 | TAOK3 | -1.331326626 | 0.0418712261876428 | down |
| 749 | R3HDM1 | -1.328315053 | 0.033097985904303 | down |
| 750 | FCHO2 | -1.321776091 | 0.0326155218400692 | down |
| 751 | ASPSCR1 | 1.32026488480925 | 0.0385776472712398 | up |
| 752 | SERINC1 | -1.318278732 | 0.044047854683952 | down |
| 753 | TTC8 | -1.315181878 | 0.0474322773859429 | down |
| 754 | CGGBP1 | -1.311362168 | 0.0359974884207908 | down |
| 755 | SH3RF1 | -1.31055313 | 0.0268238674474636 | down |
| 756 | PDLIM7 | 1.31037148381182 | 0.0388107279058296 | up |
| 757 | KLHL2 | -1.30994921 | 0.0316004526578205 | down |
| 758 | TANK | -1.309181817 | 0.0269119949815965 | down |
| 759 | PKD1 | 1.30571288947885 | 0.030923666172556 | up |
| 760 | MIGA1 | -1.302820922 | 0.0451699946835266 | down |
| 761 | CLINT1 | -1.299674222 | 0.0426544909853627 | down |
| 762 | PMEPA1 | 1.29944553101779 | 0.04830126654838 | up |
| 763 | NSF | -1.297887849 | 0.0490423131441581 | down |
| 764 | SLC35F5 | -1.296373269 | 0.0411786852402791 | down |
| 765 | TRMU | 1.29567343918404 | 0.04208854125184 | up |
| 766 | HS2ST1 | -1.294951512 | 0.040702977318912 | down |
| 767 | SBF1 | 1.29255203214981 | 0.0400403690316512 | up |
| 768 | PTAR1 | -1.276915353 | 0.0436919124039355 | down |
| 769 | LEMD3 | -1.265323674 | 0.0461741030247457 | down |
| 770 | KIF13B | -1.263623639 | 0.0446818169400435 | down |
| 771 | SRFBP1 | -1.256582002 | 0.0489935205165103 | down |
| 772 | NDUFAF2 | -1.252617029 | 0.0484790989604346 | down |
| 773 | TBCA | -1.250921948 | 0.042011182877321 | down |
| 774 | FAM172A | -1.24958605 | 0.036313734088229 | down |
| 775 | RAPGEF2 | -1.24342873 | 0.0362570155339521 | down |
| 776 | POLK | -1.236148486 | 0.0412263696704766 | down |
| 777 | LNPEP | -1.23220327 | 0.0472009498216711 | down |
| 778 | PANK3 | -1.225356626 | 0.0452899228184186 | down |
| 779 | RIC8B | -1.203032277 | 0.0464162825438092 | down |
| 780 | CCDC91 | -1.174883064 | 0.0467096853936326 | down |
